# Platelet lysate for expansion or osteogenic differentiation of bone marrow mesenchymal stem cells for 3D tissue constructs

**DOI:** 10.1016/j.reth.2023.07.011

**Published:** 2023-08-11

**Authors:** Luis Oliveros Anerillas, Mikael Wiberg, Paul J. Kingham, Peyman Kelk

**Affiliations:** a Department of Integrative Medical Biology, Umeå University, 901 87 Umeå, Sweden; b Department of Surgical & Perioperative Sciences, Section for Hand and Plastic Surgery, Umeå University, 901 87 Umeå, Sweden

**Keywords:** Foetal bovine serum, Human platelet lysate, Mesenchymal stem cells, Osteogenesis, 3D

## Abstract

**Background:**

The use of mesenchymal stem cells (MSCs) for the development of tissue-engineered constructs has advanced in recent years. However, future clinically approved products require following good manufacturing practice (GMP) guidelines. This includes using alternatives to xenogeneic-derived cell culture supplements to avoid rejection of the transplants. Consequently, human platelet lysate (PLT) has been adopted as an affordable and effective alternative to foetal bovine serum (FBS) in traditional 2D cultures. However, little is known about its effect in more advanced 3D culture systems.

**Methods:**

We evaluated bone marrow MSCs (BMSCs) proliferation and CD marker expression in cells expanded in FBS or PLT-supplemented media. Differentiation capacity of the BMSCs expanded in the presence of the different supplements was evaluated in 3D type I collagen hydrogels. Furthermore, the effects of the supplements on the process of differentiation were analyzed by using qPCR and histological staining.

**Results:**

Cell proliferation was greater in PLT-supplemented media versus FBS. BMSCs expanded in PLT showed similar osteogenic differentiation capacity in 3D compared with FBS expanded cells. In contrast, when cells were 3D differentiated in PLT they showed lower osteogenesis versus the traditional FBS protocol. This was also the case for adipogenic differentiation, in which FBS supplementation was superior to PLT.

**Conclusions:**

PLT is a superior alternative to FBS for the expansion of MSCs without compromising their subsequent differentiation capacity in 3D. However, differentiation in PLT is impaired. Thus, PLT can be used to reduce the time required to expand the necessary cell numbers for development of 3D tissue engineered MSC constructs.

## 1 Introduction

Mesenchymal stem cells (MSCs) are postnatal multipotent somatic cells, found in multiple tissues throughout the body where their main function is physiological cell replenishment [1,2]. In addition, they also possess important cell regulatory and immunomodulatory properties that make them suitable for transplantation and tissue engineering purposes [3,4]. However, the clinical use of MSCs requires following good manufacturing practice (GMP) guidelines, which include the substitution of commonly used xenogeneic sera with GMP-approved cell culture supplements [5,6].

According to the International Society for Cellular Therapy, MSCs are characterized by plastic adherence, expression of specific cell surface markers (CD73, CD90 and CD105) and the absence of hematopoietic antigens (CD45, CD34, CD14, CD19 and HLA-DR), together with the capacity to differentiate into osteoblasts, adipocytes, and chondrocytes. MSCs were discovered by Friedenstein in 1970 and have since been the subject of extensive research in the field of tissue engineering [7,8]. MSCs can be harvested from a plethora of tissues [9], among which bone marrow MSCs (BMSCs) are the most studied. MSCs have been proven to modulate the host innate and adaptive immune systems via cell-to-cell interactions or cytokine secretion. In addition, they are immune-evasive [10,11]. They have also been shown to secrete extracellular vesicles into the extracellular space to regulate and maintain tissue homeostasis through mechanisms of paracrine signaling [12,13]. MSCs can act as potent pro- and anti-inflammatory, angiogenic, and self-renewal-inducing cells, which makes them suitable to be used in regenerative medicine/advanced therapy applications [[14], [15], [16], [17]]. These new therapies offer the potential to overcome currently untreatable diseases such as graft-versus-host disease (GvHD), diabetes type I, spinal cord injury, and also to create transplantation material when reconstruction is needed [16,18].

MSCs can be embedded into hydrogels to create 3D constructs that can simulate living tissue by promoting 3D cell-to-cell interactions, solubility gradients for nutrients and cytokines, and a realistic cell-to-extracellular matrix (ECM) interaction [19,20]. These 3D systems are used to study cell behavior, the processes involved in tissue genesis, and the design of advanced transplantation models [[21], [22], [23], [24]]. 3D transplantation models have many advantages compared with their 2D- and liquid-form counterparts since their shape, composition and structure can be more precisely engineered and thus mimic healthy tissues both *in vitro* and *in vivo* [22]. For clinical application, products need to be produced under GMP guidelines and should be free from animal-derived cell culture supplements which may evoke immune reaction and the transmission of prions or other zoonotic infections after transplantation [25,26]. Currently, the most commonly used supplement is foetal bovine serum (FBS), which contains growth factors, hormones, adhesion proteins and other essential factors for cell proliferation and maintenance. The use of such a xenogeneic supplement is thus only appropriate for *in vitro* research and not for clinical applications. Moreover, despite its costly manufacturing requirements, FBS presents high batch-to-batch variation and an undefined molecular composition [27].

Although multiple commercially available xeno-free cell culture media and supplements are available, human platelet lysate (PLT) is one of the popular alternatives to xenogeneic supplements [[27], [28], [29]]. PLT is produced by multiple freeze-thawing cycles, sonication or thrombin exposure to lyse platelets from an isolate, generating a solution rich in bioactive molecules. Interestingly, PLT can be produced from expired transfusion material from blood banks that cannot longer be used for transfusion, being of similar quality to lysates produced from fresh platelet samples [30]. This makes PLT a relatively sustainable resource for research applications [5,[27], [28], [29],^31^,^32^].

Previously we compared the growth and differentiation quality of BMSCs in both 2D and 3D environments in the form of on-plastic cell culture and collagen hydrogels respectively. We showed that a 3D environment is more relevant for the study and development of histological models and transplantation constructs [33]. To date, there are few examples of xeno-free media supplements being tested on clinically relevant 3D cultures [34,35]. Therefore, in this study we investigated PLT as a xeno-free cell growth supplement for the replacement of FBS in our 3D-cell cultures. We analyzed BMSC multipotency, cell surface marker expression, and differentiation quality using FBS and PLT as supplements in both the expansion and differentiation stages.

## 2 Materials and methods

### 2.1 Tissue harvest, cell isolation and culture

Bone marrow was collected from the iliac crest or ulnar bone from four human donors as previously described [33] (See Supplementary Table 1). The samples were washed with Minimum Essential Medium-α (α-MEM; Invitrogen, Waltham, MA, USA) containing 10% (v/v) foetal bovine serum (FBS; Sigma Aldrich, St. Louis, MO, USA) and 1% (v/v) penicillin–streptomycin (Gibco, Thermo Fisher Scientific, Waltman, MA, USA) followed by centrifugation at 300×*g* for 5 min. The cell suspension was then filtered through a 70-μm pore diameter nylon mesh (BD Falcon, Franklin Lakes, New Jersey, USA), subsequently plated in 75 cm^2^ tissue culture flasks (Nunc) and incubated at 37 °C with 5% CO_2_. Non-adherent cells and debris were removed after 24 h of culture while the attached cells were cultured for 2–3 weeks with medium changes every 48 h. Cultures were passaged when the cells reached 80–90% confluence. For storage conditions, cells were frozen at −80 °C in 90% (v/v) FBS with 10% (v/v) dimethyl sulfoxide solution prior to being moved to liquid nitrogen. Thawing was done at 37 °C prior to cell seeding at a cell density of 1 × 10^4^ cells/25 cm^2^ flask, initially in α-MEM supplemented with 20% (v/v) FBS and 10 ng/ml of basic fibroblast growth factor (bFGF; PeproTech, London, UK). Cell seeding density was at 5000 cells/cm^2^.

hFOB 1.19- CRL-11372™ (ATCC®, Manassas, VA, USA) cells, an immortalized osteoblastic cell line, were expanded and cultured following the manufacturer's instructions until they were selectively expanded in either FBS- or PLT-supplemented media (α-MEM containing either 10% (v/v) FBS or 5% (v/v) PLT plus 1% (v/v) penicillin-streptomycin.

### 2.2 Cell proliferation and colony-forming unit assay

BMSCs were first cultured from each individual donor in triplicates at passage 1 on a 6-well plate in α-MEM supplemented with either 20% (v/v) FBS or PLT (PLTGold®, Sigma Aldrich) at 2%, 5% or 10% concentrations. The growth medium was changed every other day and the cells were trypzinized and counted once any of the cultures had reached 80–90% confluence. The populations were counted with a haemocytometer and re-seeded in a new 6-well plate with 10^4^ cells/well. Cell cultures were expanded for 35 days. Population doublings (PDs) were calculated using the following equation:

PD=[durationxlog(2)]/[log(Finalcellnumber)−log(Initialcellnumber)]

In addition, 300 cells from all four donors at passage 1 were seeded into a 25-cm^2^ flask and cultured for 2 weeks without medium change followed by fixation and staining with a 0.1% (w/v) toluidine blue, 2% (w/v) paraformaldehyde solution for 1 h. The flasks were rinsed with distilled water to remove the dye excess. The number of colonies with ≥50 cells were measured.

### 2.3 Cell characterization by flow cytometry

BMSCs after 1 week of expansion were examined for the expression of MSC-associated CD markers (CD73, CD90 and CD105) and stained with a negative marker antibody cocktail (CD11b, CD19, CD34, CD45 and HLA-DR). According to the manufacturer's protocol (BD Biosciences, Allschwil, Switzerland), BMSCs were incubated with phycoerythrin (PE)-conjugated antibodies [CD73 (1:25), CD90 (1:33) or CD105 (1:25)] and negative marker cocktail [CD11b, CD19, CD34, CD45 and HLA-DR (1:25)]. PE mouse IgG1κ isotype control (BD Pharmingen) was used as control for all positive CD markers. PE human MSC (hMSC) isotype control, negative cocktail (BD Stemflow) was used as an isotype negative control. The antibody list is shown in Table 1. A total of 5 × 10^4^ cells for each donor were collected per antibody and 10^4^ cells were analyzed after SSC/FFC-gating using an Accuri™C6 Plus flow cytometer (BD Bioscience). Cell cultures were expanded in either FBS- or PLT-supplemented media for 5 weeks before they were collected and treated as described above.

**Table 1:** Antibodies used for flow cytometry.

Antibody	Brand	Clone number
CD73	BD Pharmingen™	Mouse IgG1, κ AD2
CD90	BD Pharmingen™	Mouse BALB/c IgG1, κ 5E10
CD105	BD Pharmingen™	Mouse BALB/c IgG1, κ, 266
CD146	BD Pharmingen™	Mouse IgG1, κ, P1H12
PE mouse IgG1, κ isotype control	BD Pharmingen™	Mouse IgG1, κ MOPC-21
PE hMSC negative cocktail	BD Stemflow™	Mouse IgG1, κ CD34 (Clone 581) CD11 b PE (Clone: ICRE44) CD19 PE (Clone: HIB19) CD45 PE (Clone: HI30) HLA-DR PE (Clone G46–6)
PE hMSC negative isotype control cocktail	BD Stemflow™	Mouse IgG1, κ, PE (Clone × 40)

### 2.4 3D cell culture

BMSCs were trypsinized, counted and mixed with a neutralized solution of type I collagen (∼4 mg/ml, CELLINK, Gothenburg, Sweden) on ice; followed by their casting onto 48-well plates. The plates were incubated for 30 min at 37 °C to assure proper collagen gelation. The typical number of cells in each gel was set to 3 × 10^5^ as described previously [33]. Cell culture medium was added and after 1 day of incubation, the gels were moved from a 48-well plate to a 24-well plate to maximize exposure to medium. The neutral pH of the collagen mix was verified and/or adjusted with 1 N NaOH and 0.1 M HCl prior to the addition of the cells and the casting.

To make the pellet models used in the chondrogenic experiments, 5 × 10^5^ BMSCs were centrifuged at 200×*g* for 5 min and were left undisturbed in cell culture medium for 24 h prior to the differentiation.

### 2.5 2D and 3D cell differentiation

An initial phase of expansion in either 10% FBS or 5% PLT for a week was performed prior to some differentiation experiments. The differentiation process started 24 h after the gels were cast or the 2D cultures plated. The samples were then treated with differentiation media:

- Osteogenic differentiation - Dulbecco's Modified Eagle Medium (DMEM; low glucose + Glutamax) with 10% (v/v) FBS or 5% (v/v) PLT, 1% (v/v) penicillin–streptomycin and 100 nM dexamethasone, 10 mM β-glycerophosphate and 0.2 mM 2-phosphate-l-ascorbic acid. Alternatively, the complete mix of differentiating factors was applied (StemPro® Osteogenesis Supplement; Gibco).
- Adipogenic differentiation - DMEM (low glucose + Glutamax) with 10% (v/v) FBS or 5% (v/v) PLT, 1% (v/v) penicillin–streptomycin, 1 μM dexamethasone, 10 μg/ml insulin, 0.5 mM 3-isobutyl-1-methylxanthine and 100 μM indomethacin.
- Chondrogenic differentiation - serum-free (or containing 5% PLT) α-MEM supplemented with 40 μg/ml l-proline, 100 μg/ml sodium pyruvate, 100 μM dexamethasone, 1% (v/v) Insulin-Transferrin-Selenium solution, 50 μg/ml ascorbate 2-phosphate and 10 ng/ml transforming growth factor-β3.

Both differentiation and control media were changed every other day and samples collected after 1, 3 and 5 weeks in osteogenesis experiments, 3–4 weeks in adipogenesis experiments and 4–5 weeks in chondrogenesis experiments.

### 2.6 2D and 3D cell harvest and processing

The 2D and 3D-culture samples for each differentiation and expansion condition were processed at equal time points. For every experimental group, RNA was extracted to be used in qRT-PCR analyses to measure the expression levels of typical differentiation markers of the osteogenic and adipogenic lineage. In addition, samples were either stained with a lineage-specific dye or fixed in paraformaldehyde, cryosectioned (12 μm sections) and used for immunohistochemistry and thereafter in optical and fluorescent microscopy.

### 2.7 Histochemistry

Histochemical stainings to confirm osteogenic, adipogenic and chondrogenic differentiation were performed on cell culture well-plates for 2D cultures and in cryosectioned samples mounted on glass slides for 3D cultures.

Osteogenesis was analyzed by Osteoimage® (Lonza, Walkerville Inc., Australia) fluorescent staining kit following the manufacturer's protocols modified for small specimens; and by pre-filtered 1% Alizarin red (ThermoFisher Scientific) solution, which was added for 3 min to previously washed sections (with PBS), fixed with 60% (v/v) isopropanol and rehydrated with deionized water. The dye excess was washed with distilled water before mounting the sections with PERTEX® on glass slides for imaging. Osteoimage® sections were mounted with DAPI Prolong mounting medium (Life Technologies, Carlsbad, CA, USA).

Adipogenesis was verified with Oil red O dye. Sections were washed with PBS and fixed with 60% (v/v) isopropanol, followed by incubation with Oil red O for 10 min at room temperature. Thereafter, a final wash was performed with 60% (v/v) isopropanol and samples rehydrated with PBS.

Chondrogenic differentiation was assessed by Masson's trichrome staining (Sigma-Aldrich) by following the manufacturer's protocol and using Weigert's Iron Haematoxylin (Sigma) to counter-stain the cells. In addition, toluidine staining was also used to determine successful chondrogenic differentiation by staining with 1% toluidine blue (Merck, Darmstadt, Germany) after washing the slides.

### 2.8 Immunochemistry

The slides were washed twice with buffer (0.01 M PBS, 0.1% NaN_3_ and 0.1% (w/v) BSA) for 30 min and blocked with 5% (v/v) goat normal serum (diluted in buffer). Excess buffer was removed and the slides incubated overnight at 4 °C with primary antibody (Table 2) dissolved in buffer, followed by 4 washes of 15 min with buffer and a secondary block with 5% (v/v) goat normal serum in buffer. The slides were incubated in the dark for 1 h with the secondary antibody (Table 2) diluted into buffer and washed with buffer. Sections were mounted with DAPI Prolong mounting medium.

**Table 2:** Antibodies used for immunohistochemistry.

Type	Antibody	Reference	Dilution
Primary	Mouse monoclonal, anti-OCN	Novus Biologicals, H00000632-M01	1:100
Primary	Mouse monoclonal, anti-OPN	Novus Biologicals, NB110-89062	1:100
Primary	Mouse monoclonal, anti-ALPL	R&D Systems, MAB1448	1:100
Secondary	Alexa FluorTM 488 Goat anti mouse	ThermoFisher, A11029	1:1000

### 2.9 Quantitative RT-PCR

Total RNA was extracted from 2D and 3D BMSC cultures using RNeasy mini kits according to the manufacturer's instructions (Qiagen®, Hilden, Germany). RNA concentration was measured with a Nanodrop-2000c spectrophotometer (ThermoFisher Scientific). For each sample, 250 ng of complementary DNA (cDNA) was synthesized using iScript cDNA synthesis kit (Bio-Rad, CA, USA). Five ng of cDNA were used per reaction along with SsoFast™EvaGreen® supermix (Bio-Rad) in a CFX96 Optical Cycler and analyzed using the CFX96 manager sofware (Bio-Rad). Ribosomal protein L13a (RPL13a) gene was used as a housekeeping gene. The data were calculated and reported as relative gene expressions according to the ΔΔC(t) principle. The list of primers used are shown in Table 3.

**Table 3:** Primers used for quantitative RT-PCR analysis.

Primer	Forward (5′-3′)	Reverse (5′-3′)	Annealing temperature (°C)
OPN	GCCGACCAAGGAAAACTCACT	GGCACAGGTGATGCCTAGGA	64.7
OCN	AGCAAAGGTGCAGCCTTTGT	GCGCCTGGGTCTCTTCACT	63.2
AP2	GGTGGTGGAATGCGTCATG	CAACGTCCCTTGGCTTATGC	64.1
ALPL	GGAACTCCTGACCCTTGACC	TCCTGTTCAGCTCGTACTGC	65.2
GLUT4	AGCAGCTCTCTGGCATCAAT	CTACCCCTGCTGTCTCGAAG	61
PPARγ	CCGAGAAGGAGAAGCTGTTG	TCGGATATGAGAACCCCATC	60.8
RPL13a	AAGTACCAGGCAGT GACAG	CCTGTTTCCGTAG CCTCATG	58
COL14A1	TCCGAGGAATGGTATAACCGG	TGGACCAGGAACACTGACAGG	64.1
COL10A1	GCCCAGCAGGAGCAAAGG	GGACCGGGACTTCCTGGAT	64.95

### 2.10 NanoString® nCounter®

NanoString™ nCounter®, a molecular barcode-based hybridization reaction analysis system, was used to analyze 2D and 3D culture BMSCs with or without osteogenic stimulation. A metabolic gene array against 748 metabolic genes was used (for summary see Supplementary Table 2). A total of 12 RNA samples (N = 12) were analyzed from three donors. All data processing and analysis was done with nSolver® software and presented as volcano plots as a gene expression ratio.

### 2.11 Alizarin red quantification

BMSC from donor 2 and hFOB 1.19 cells, previously expanded in FBS or PLT, were plated on an 8-well culture slide and osteogenically differentiated for 3 weeks. The individual wells were stained with alizarin red as described earlier and the slides scanned with an Epson Scanner. The images were cropped with Adobe Photoshop (San José, CA, USA) to remove the labels and the images converted to black and white format. White over black levels were quantified by Fiji ImageJ software and the numerical data analyzed with Prism.

### 2.12 Statistical analysis

qRT-PCR data and histological images were analyzed with unpaired Student's *t*-test between the individual sample groups (GraphPad Prism®, GraphPad Software Inc., San Diego, CA, USA). Metabolic gene expression with nSolver® software was subjected to Benjamini-Hochberg false discovery rate correction. Statistical significance was set as ∗ p < 0.05, ∗∗p < 0.01, ∗∗∗p < 0.001.

## 3 Results

### 3.1 PLT increases BMSC proliferation in 2D cultures

**Fig. 1:**
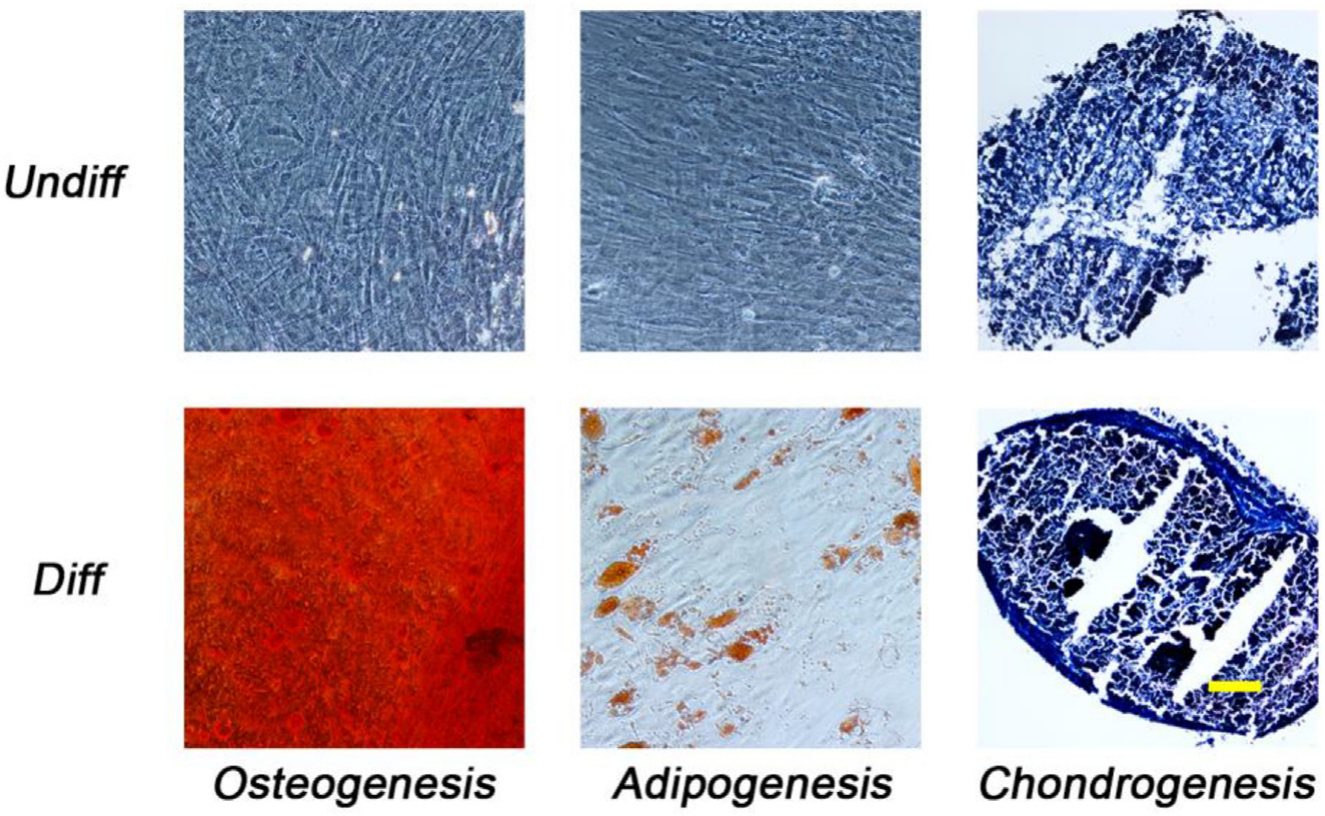
Verification of BMSC differentiation potential. Undifferentiated (Undiff) and differentiated (Diff) BMSCs were stained with the appropriate lineage-specific dye: Osteogenesis (Alizarin Red), adipogenesis (Oil Red O) and chondrogenesis (Masson's Trichrome). Osteogenesis and chondrogenesis groups were differentiated for 5 weeks while adipogenic samples were for 4 weeks. The images show one representative donor. Scale bar = 100 μm.

**Fig. 2:**
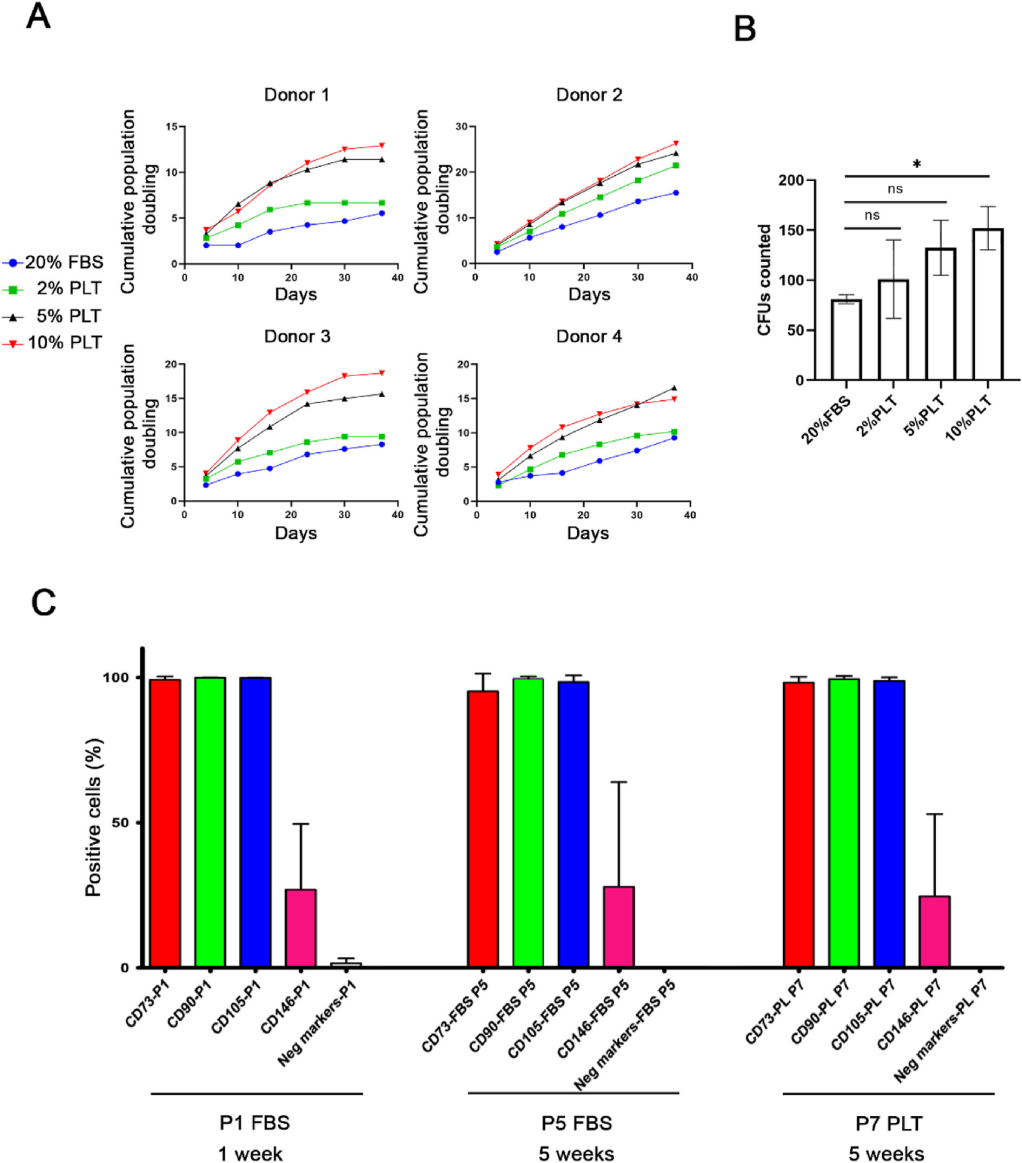
Characterization of MSCs in either FBS or PLT growth conditions. **(A)** Cumulative population doubling of individual donor's cells in culture media supplemented with 2%, 5%, 10% PLT or 20% FBS (n = 4). **(B)** Number of colony forming units (CFUs) after two weeks of culture in either 20% FBS or 2%, 5%, or 10% PLT supplemented culture medium (n = 3). **(C)** Flow cytometry analyses of MSC-associated CD markers (CD73, CD90, CD105, and CD146) and expression of negative cell surface markers (CD11b, CD19, CD34, CD45 and HLA-DR) after growth in FBS or PLT. The significance levels are indicated as ∗ (*p*-value <0.05); ns (non-significant).

BMSCs were initially differentiated in 2D for 4 weeks (adipogenesis) and 5 weeks (osteogenesis and chondrogenesis) to verify their multipotency (Fig. 1). In addition, FBS- and PLT-expanded BMSCs were examined in 2D for adipogenic differentiation quality in both FBS- and PLT-containing media for 3 weeks. The results suggest that adipogenesis is impaired if supplemented with PLT (Supplementary Fig. 1A). A similar experiment was performed for chondrogenesis, in previously expanded BMSCs, in either FBS or PLT. However, chondrogenesis requires the lack of serum in the differentiation media, so FBS-expanded cultures were differentiated in absence of any sera, while PLT-expanded ones were differentiated in the presence of PLT for 4 weeks. The results indicate that the differentiation process is preferred in the absence of serum (Supplementary Fig. 1B). BMSCs from all four donors were then cultured in either 20% FBS or 5% PLT for 5 weeks and passaged individually once each flask reached 80–90% cell confluence. Proliferation experiments showed that cumulative population doublings were higher for all 4 donors cultured under various concentrations of PLT (2–10%) compared with the 20% FBS groups (Fig. 2A). These results were mirrored by analyses of colony-forming unit fibroblastic (CFUf) potential, which showed PLT-dose dependent increases versus the FBS-cultured cells (Fig. 2B). Flow cytometry analyses showed no significant difference in MSC-associated cell surface markers between BMSCs cultured in FBS or PLT after 5 weeks of culture, at which point the FBS-expanded cells were at passage 5 while PLT-cultured cells reached passage 7 (Fig. 2C).

### 3.2 Heme oxygenase 1 gene (*HMOX1*) is differentially expressed in FBS- versus PLT-expanded cells

**Fig. 3:**
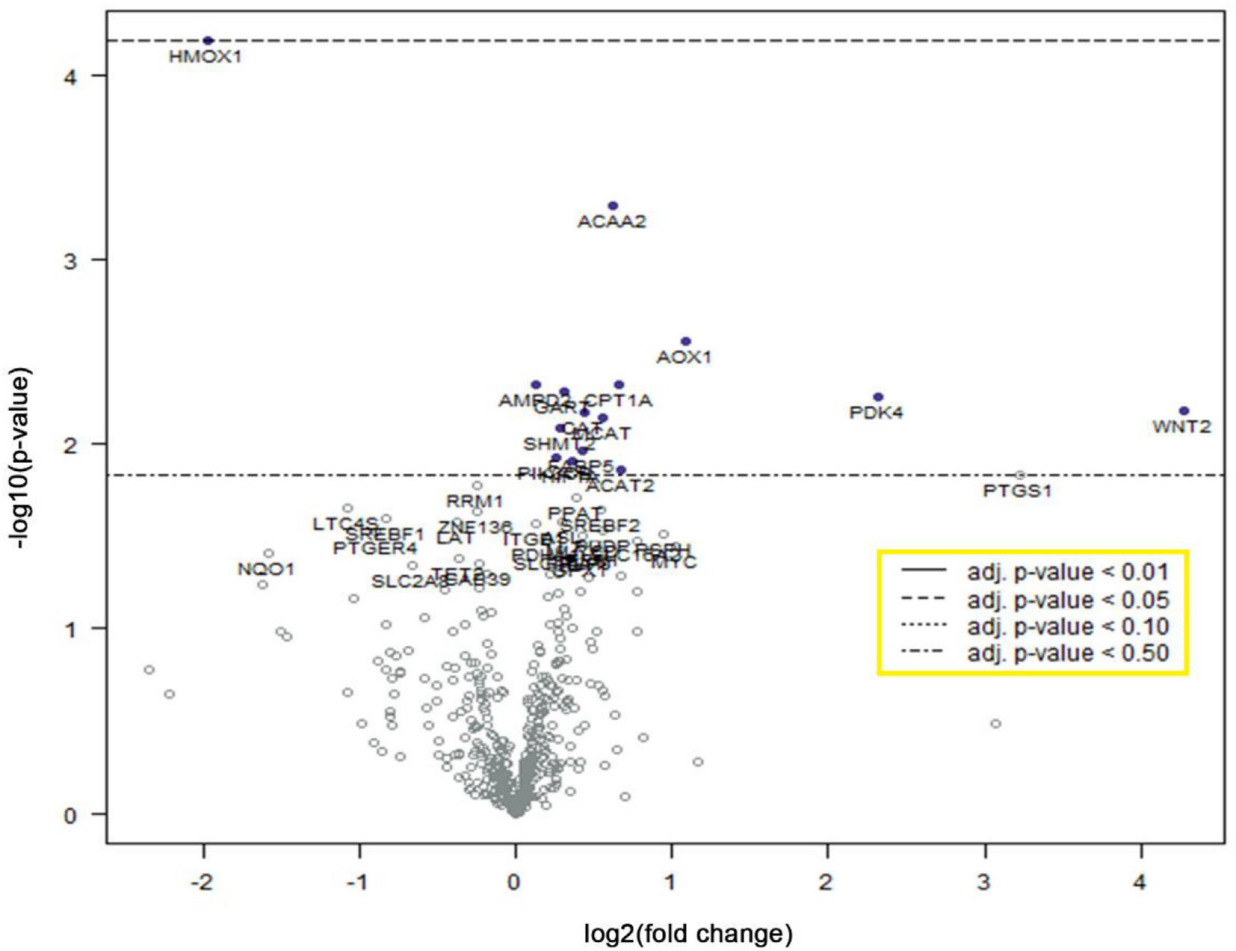
**Differential gene expression between FBS- and PLT-cultured samples in baseline (undifferentiated) conditions.** Multivariate analysis of the 748 metabolic genes (NanoString® kit), after 3 weeks of culture, displayed as a volcano plot (created with nSolver® software) with the media supplement selected as covariate. The plot shows each gene's –log_10_ (*p*-value) and log_2_ fold change associated to PLT culture condition compared with FBS. Highly statistically significant genes fall at the top of the plot above the indicated *p*-value lines, and highly differentially expressed genes fall to either side (right = upregulated, left = downregulated). A single down-regulated gene, *HMOX1*, was considered significant (*p-*value <0.05).

We explored the possibility that FBS and PLT would activate different metabolic pathways by screening for a total of 748 metabolic genes using a nanoString® kit. The results were analyzed with nSolver® software and plotted by differential expression and the associated *p*-value (Fig. 3). A single gene, *HMOX1*, was significantly (p < 0.05) differentially expressed, showing a 4 fold down regulation in the PLT versus FBS expanded cells.

### 3.3 Osteogenic differentiation in 2D is similar in FBS- and PLT-expanded cells

**Fig. 4:**
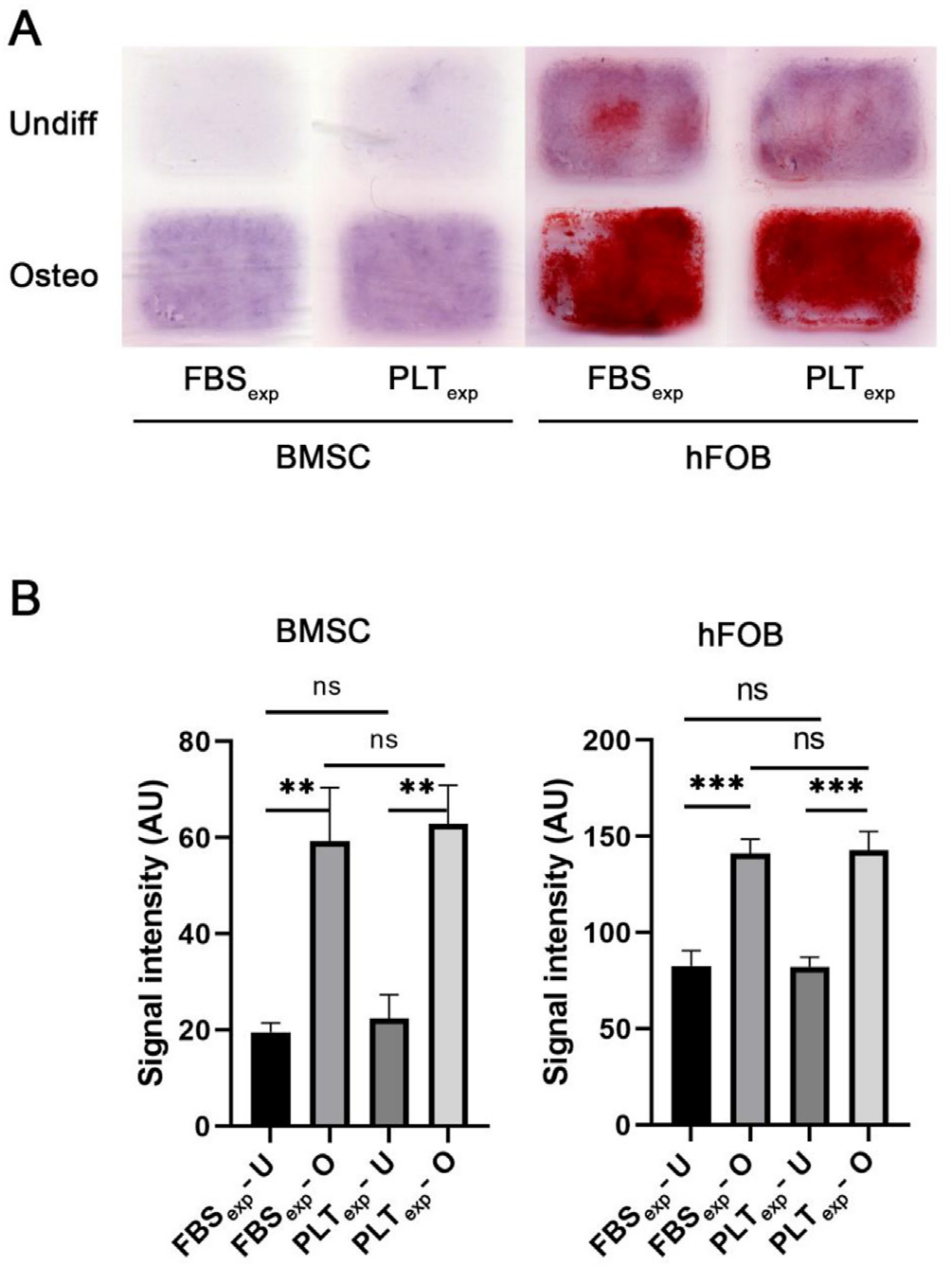
Osteogenic differentiation of FBS- or PLT-expanded (labelled as FBS_exp_ or PLT_exp_) BMSCs and osteoblastic hFOB 1.19 cell line. **(A)** Alizarin red staining of BMSCs and hFOB 1.19 cells either undifferentiated (undiff) or differentiated (Osteo) for 3-weeks. The cells were previously expanded in either FBS or PLT. The figure shows representative images. **(B)** Quantification of stained signal intensity in arbitrary units (AU; n = 3). The graph shows the mean ± SD of the sample groups. ns = non-significant differences between the respective undifferentiated (U) and differentiated (O) groups.

BMSCs were cultured in parallel with an immortalized osteoblast cell line, hFOB 1.19, expanded in either FBS or PLT and then differentiated for 3 weeks in FBS. The resulting cultures were stained with alizarin red to highlight the accumulation of Ca^2+^ (Fig. 4A). The osteogenic hFOB 1.19 cells showed high mineralization levels with some indication of auto-differentiation. Similarly, BMSCs showed a clear difference in the osteogenic group compared with the control, though it was comparatively much lower than hFOB 1.19 cells. The intensity of the staining was quantified and showed no significant differences between FBS- and PLT-expanded groups (Fig. 4B). Taken together, these results indicate that the expansion of BMSCs and hFOB 1.19 cells in either FBS or PLT does not affect the process of osteogenic differentiation.

### 3.4 Osteogenic differentiation of BMSCs in type I collagen hydrogels is similar between FBS- and PLT-expanded cells

**Fig. 5:**
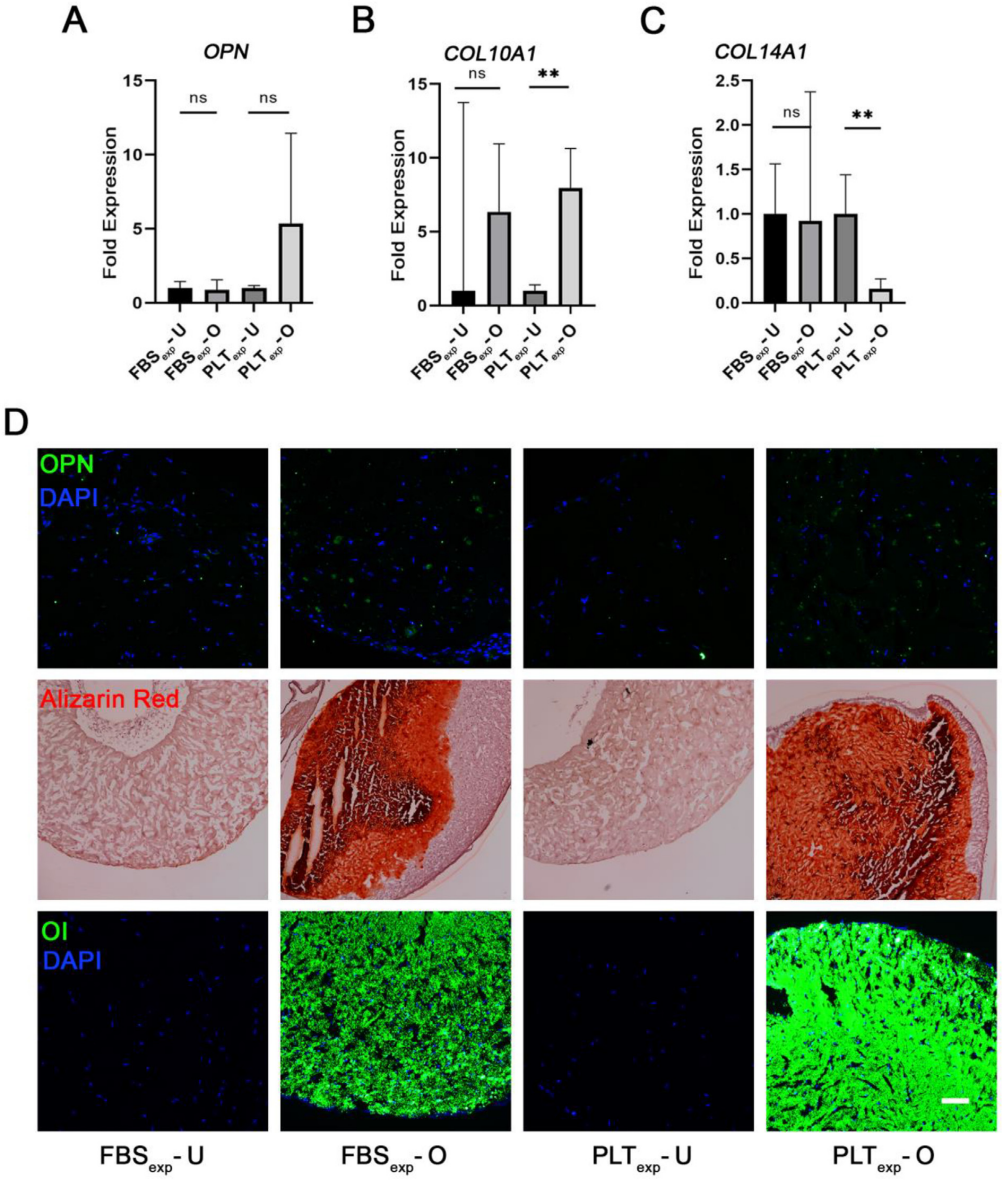
Osteogenic differentiation for 5 weeks in FBS after expansion in either FBS or PLT (FBS_exp_ or PLT_exp_). **(A**–**C)** Quantitative RT-PCR analyses of relevant osteogenesis-related genes (*OPN, COL10A1, COL14A1*) in undifferentiated (U) or osteogenic (O) conditions after differentiation in FBS-supplemented medium for 5 weeks. BMSC cultures were expanded in FBS or PLT for 1–2 weeks prior to the differentiation. **(D)** Immunohistochemical/histochemical staining of gel sections against osteopontin protein (OPN); Ca^2+^ ions with alizarin red, and hydroxyapatite with Osteoimage reagent kit. Statistical significance indicated as ns (non-significant) and ∗∗ (p-value <0.01); n = 4. Scale bar = 100 μm.

BMSCs were expanded in FBS or PLT prior to 5-week osteogenic differentiation in FBS in collagen gels. Osteogenic-related genes were analyzed with qRT-PCR. No significant differences in *OPN* gene expression were observed (Fig. 5A). Significant *COL10A1* up-regulation and *COL14A1* down-regulation were detected in the PLT-expanded group exposed to osteogenic differentiation (Fig. 5B and C). Immunohistochemical staining for OPN, histochemical staining with alizarin red against Ca^2+^ ions, and fluorescent staining of hydroxyapatite with OsteoImage® (Lonza) showed similar levels of osteogenesis in the FBS and PLT expanded cells (Fig. 5D).

### 3.5 Osteogenic differentiation is less effective in medium containing PLT

**Fig. 6:**
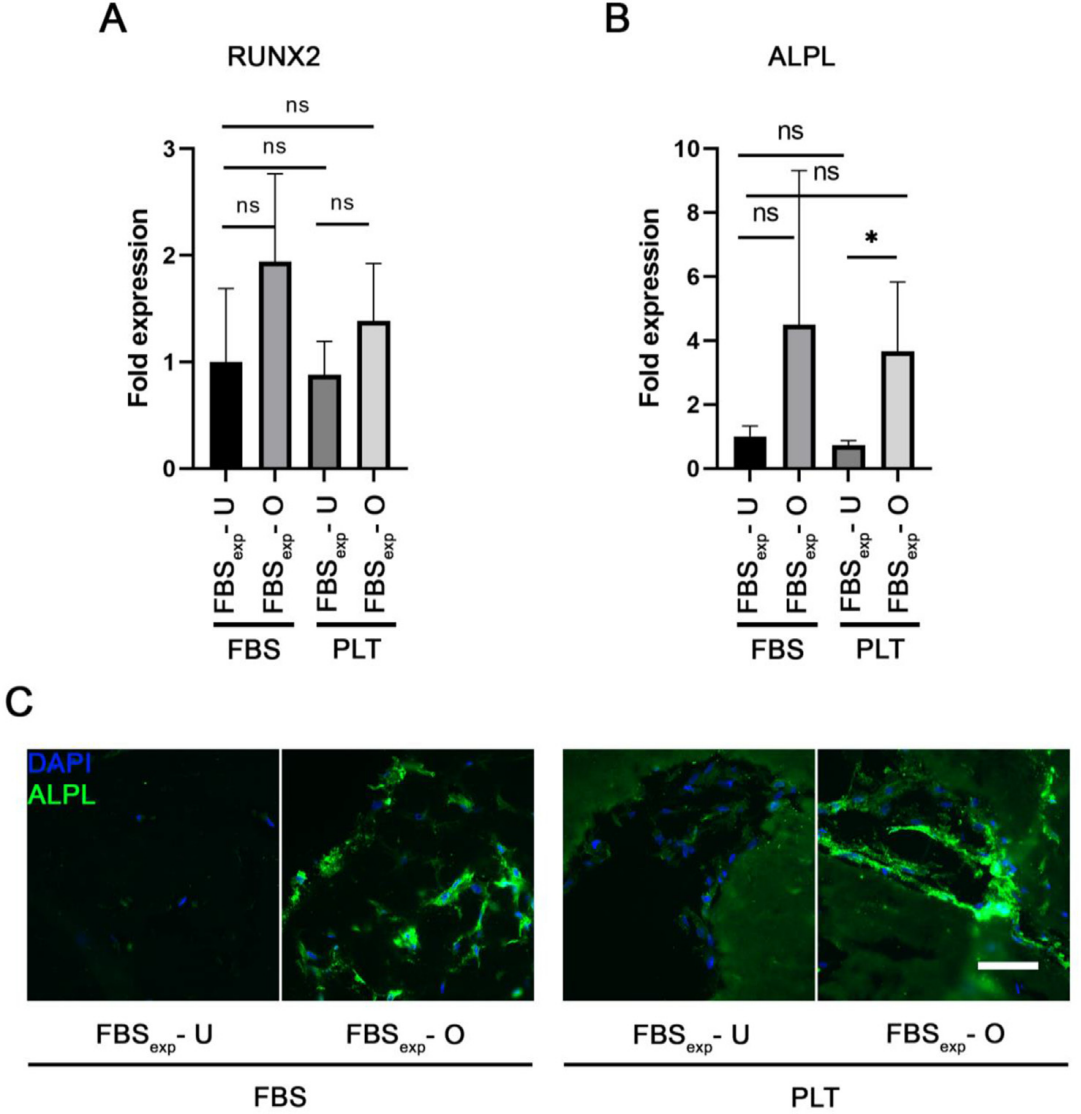
Osteogenic differentiation for 3 weeks in FBS- or PLT-supplemented media. All sample groups were previously expanded in FBS (FBS_exp_). **(A & B)** Quantitative RT-PCR analysis of relevant osteogenesis related genes (*RUNX2, ALPL*) in undifferentiated (U) or osteogenic (O) conditions in FBS or PLT. **(C)** Immunohistochemical staining of gel sections against ALPL protein and nuclear DAPI in a 3D type I collagen gel from a representative donor after 3 weeks in various conditions. n = 3–4. Scale bar = 100 μm.

**Fig. 7:**
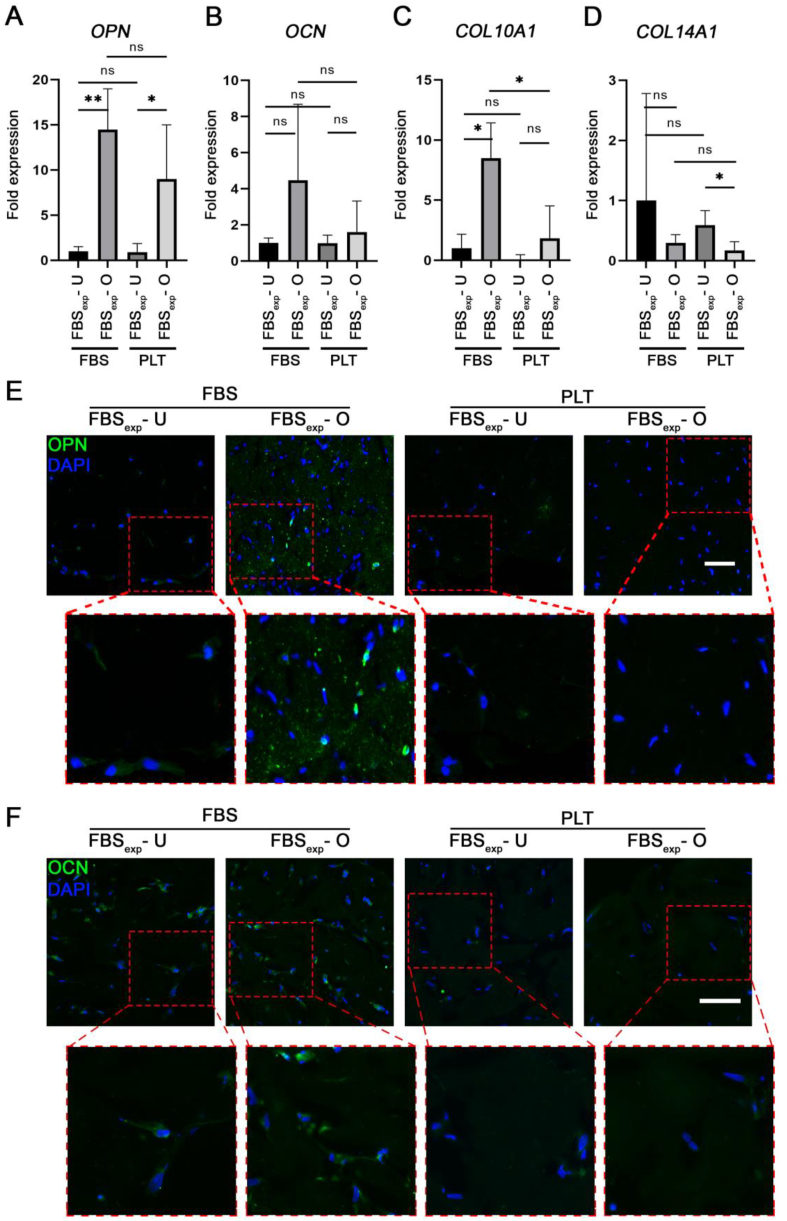
Osteogenic differentiation for 5 weeks in FBS or PLT. All sample groups were previously expanded in FBS (FBS_exp_). **(A**–**D)** Quantitative RT-PCR analyses of relevant osteogenesis-related genes (*OPN, OCN, COL10A1, COL14A1*) in undifferentiated (U) or osteogenic (O) conditions after differentiation in either FBS- or PLT-supplemented medium for 5 weeks. Immunohistochemical staining of gel sections against **(E)** osteopontin (OPN) and **(F)** osteocalcin (OCN). Red insets highlight the respective magnified area. Statistical significance indicated as ns (non-significant) and ∗∗ (p-value <0.01); n = 4. Scale bar = 100 μm.

Next, we examined the osteogenic differentiation in medium containing PLT. Firstly, BMSCs in type I collagen gels were differentiated for three weeks. The samples were analyzed by qRT-PCR expression for middle-stage osteogenic genes*, RUNX2* and *ALPL* for which no significant differences were observed between the groups (Fig. 6A and B). Clear staining against ALPL protein was observed in immunostained sections (Fig. 6C), which was similar between both groups. Next, we investigated if there were differences in 3D osteogenic differentiation after 5 weeks. The qRT-PCR analyses showed a significant 14.5 fold mean increase of *OPN* in FBS and 9-fold increase in PLT (Fig. 7A). However, there was no significant difference in gene expression for *OCN* (Fig. 7B). In addition, *COL10A1* expression significantly increased 8.5 fold in differentiation medium containing FBS (Fig. 7C), while *COL14A1* was significantly down-regulated in the medium containing PLT (Fig. 7D). Overall, the gene analyses showed a trend that osteogenic genes were expressed at lower levels in the PLT differentiated samples. Immunostaining showed higher expression of OPN protein in FBS versus PLT-differentiated samples (Fig. 7E). OCN protein was only detected in the FBS-differentiated group (Fig. 7E). Collectively these results suggest that the differentiation in medium containing FBS was more efficient.

### 3.6 Adipogenic differentiation is more efficient in FBS versus PLT

**Fig. 8:**
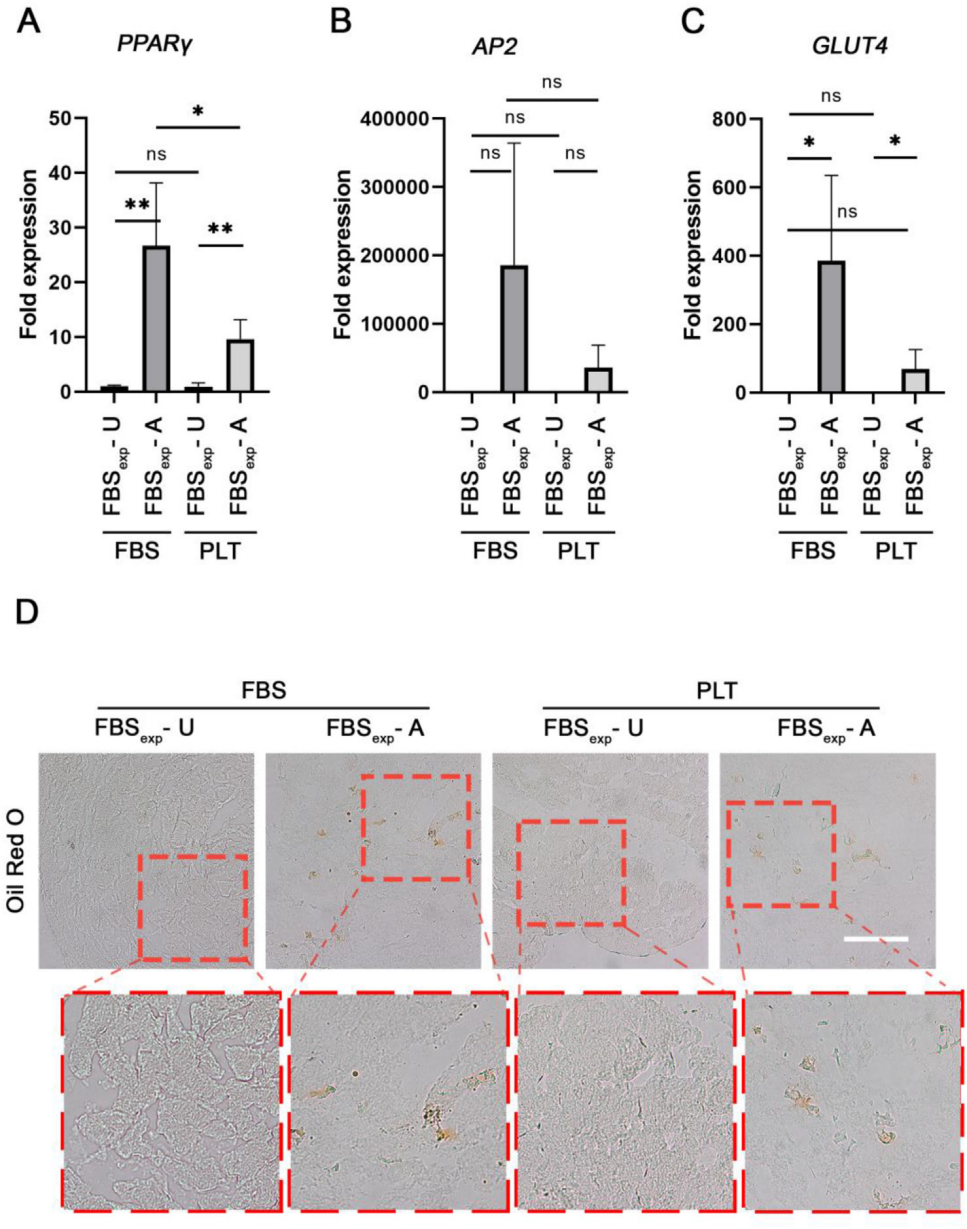
Adipogenic differentiation in FBS- or PLT-supplemented media. All sample groups were previously expanded in FBS (FBS_exp_). **(A**–**C)** Quantitative RT-PCR analysis of relevant adipogenic related genes (*PPARγ*, *AP2*, *GLUT4*) in unstimulated (U) or adipogenic (A) conditions in either FBS or PLT. **(D)** Oil-red O staining of gel sections from a representative donor are shown. Red insets highlight the respective magnified area. Statistical significance indicated as ns (non-significant) and ∗∗ (p-value <0.01); n = 4. Scale bar = 100 μm.

Analysis of gene expression showed that *PPARγ* was significantly higher in adipogenic cultures in FBS supplemented medium versus PLT medium (Fig. 8A). *AP2* and *GLUT4* expression levels were also higher in cells cultured in adipogenic medium supplemented with FBS (Fig. 8B and C). These results were consistent with the histological observations. No adipocytes were observed in undifferentiated gels, but there were more in FBS- versus PLT-differentiated gels (Fig. 8D).

## 4 Discussion

MSCs possess unique characteristics that make them suitable for transplantation and the design of advanced therapies. However, due to various ethical, safety, and scientific concerns, cellular and acellular clinical products must adhere to GMP guidelines [5,6]. A critical part of GMP guidelines highlights the possibility of viral, prionic, or bacterial infections due to zoonosis from xenogeneic cell culture supplements. For this reason, research has focused on trying to find alternatives to FBS, the most commonly used cell culture supplement. Suitable alternatives can be collected and processed from human sources such as autologous human serum, human plasma-derived supplements, umbilical cord serum/plasma, or platelet lysate [36]; the latter is being increasingly used in the field of tissue engineering [29]. This is of special interest for the development of 3D transplantation constructs in the form of hydrogels or cellular scaffolds containing autologous cells that could be safely expanded *in vitro* prior to their embedding into biologically relevant scaffolds materials such as collagen, hyaluronic acid, and other components of the extracellular matrix [35].

Our results indicate a clear increase in growth rate of BMSCs when expanded in PLT compared with FBS. This has also been widely observed in the literature [29]. Surprisingly, even our lowest PLT concentration (2%) was able to outperform a 20% concentration of FBS in terms of cell proliferation. This indicates that PLT has a very high potential use for the expansion of MSCs *in vitro*, while also being a more cost-efficient supplement than FBS*.* These observations are in line with previously reported studies that indicate that PLT contains high levels of growth factors such as BMPs, VEGF, TGF-β, BDNF, PDGF, IGF, EGF, and FGF. In addition, it also presents high concentration of plasma proteins such as albumin, protease inhibitors, immunoglobulins, minerals and glucose [37,38]. All these factors make PLT an excellent candidate to promote cellular proliferation *in* vitro. However, PLT composition will always depend on the available donor material, the method used for its processing, and regulations that mandate how PLT quality is tested and approved for commercialization [39]. Despite the presence of high concentrations of BMPs in PLT, it is possible that the osteogenic potential of these proteins is not enough to compete against the pro-proliferative molecules also present in PLT.

Interestingly, when we compared the gene expression between FBS- and PLT-supplemented BMSCs (in undifferentiated conditions) we found a linear 4-fold downregulation of a single gene, heme oxygenase 1, *HMOX1* (Fig. 3). This gene is involved in the cleavage of heme into biliverdin and its downregulation has been correlated with the overexpression of the pro-osteogenic bone morphogenetic protein 2 (BMP2) [40]. *HMOX1* regulates the generation of osteoblasts during bone fracture repair and has been reported to accelerate osteogenesis after its overexpression [41,42]. Similarly, BMP9-overexpression upregulates *HMOX1* gene expression in C3H10T1/2 cells [43]. *HMOX1*′s overexpression has also been reported to decrease adipogenesis in mice and pigs [44,45]. Regardless, the involvement of *HMOX1* in osteogenic and adipogenic differentiation has not reached a consensus yet. Some studies suggest a stimulatory role while others support an inhibitory function [46,47] of *HMOX1* on both osteogenic and adipogenic differentiation pathways.

When we compared osteogenic differentiation in PLT- and FBS-expanded BMSCs in 2D and 3D constructs (Fig. 4, Fig. 5 respectively), we found similar differentiation levels between both groups. This suggests that PLT is a good substitute of FBS as an expansion supplement that does not reduce differentiation potential in the long-term. Since there are few reports showing the effect of PLT on differentiation in 3D we were particularly interested to compare FBS and PLT culture supplements in our type I collagen hydrogels containing BMSCs (Fig. 6, Fig. 7). Our results indicated similar levels of differentiation markers after 3 weeks however at 5 weeks gene expression and staining data indicated a more effective osteogenesis in the FBS-differentiated group compared with PLT. Previous studies have documented that PLT promotes osteogenesis and adipogenesis when used as culture supplement during the differentiation procedure [35,[48], [49], [50]], although a larger number of authors report similar levels of osteogenesis potential when PLT is compared with FBS [29,51,52]. As far as we are aware, this has not been explored in a 3D collagen model, however, studies in other types of gels show differing effects of PLT on the differentiation [35,53]. Interestingly, a recently published article showed that PLT improved the proliferation of pediatric human adipose-derived stem cell (ADSCs) and auricular cartilage stem/progenitor cells in both 2D and 3D (pellet) culturing systems, while impairing chondrogenic differentiation [54].

## 5 Conclusions

Taken together, our results show that PLT is an excellent cell culture supplement for increased cell proliferation of BMSCs without compromising their osteogenic differentiation capacity. Several studies are consistent with our data and conclude that PLT is useful for clinically relevant expansion of MSCs [37,55]. However, in our 3D model if PLT is added as supplement during differentiation of BMSCs, the osteogenesis in PLT is less efficient than FBS. Our results therefore suggest that PLT drives metabolism towards a proliferative phenotype, thus reducing the differentiation potential compared with FBS.

## Ethical approval and institutional review board statement

The local ethics committee for research at Umeå University (Dnr 2013-276-31 M and 03–425) approved collection, processing, culture, storage and usage of all clinical isolates in this study. All methods were performed by following the relevant guidelines and regulations of the local ethics committee. Informed consent was obtained from all cell donors according to the guidelines of the Declaration of Helsinki.

## Author contributions

PK conceived and designed the study. LOA performed the experiments. LOA and PK analyzed and interpreted the data. PK, MW and PJK contributed to research infrastructure. LOA and PK wrote the paper. Draft revision done by PJK and MW. Project supervision by PK. All authors have given approval to the final version of the manuscript.

## Funding

This project has been funded by grants from: the research fund of County Council of Västerbotten (Project Number: 7003459 and 7003589) and the 10.13039/501100010794Faculty of Medicine, Umeå University.

## Declaration of competing interest

The authors declare no conflict of interest. The study sponsors were not involved in the study design, the collection, analysis and interpretation of data, then writing of the manuscript or the decision to submit the manuscript for publication.
