# Supplementary material for: Platelet lysate for expansion or osteogenic differentiation of bone marrow mesenchymal stem cells for 3D tissue constructs
Source: Regen Ther. 2023 Aug 11;24:298–310. doi: 10.1016/j.reth.2023.07.011 (PMC10425714; doi:10.1016/j.reth.2023.07.011)
Supplement: Multimedia component 1 [file mmc1.docx]

**Supplementary material: Platelet lysate for expansion or osteogenic differentiation of bone marrow mesenchymal stem cells for 3D tissue constructs**

Running title: platelet lysate supplementation for osteogenic 3D constructs

Luis Oliveros Anerillas MSc^1*^; Mikael Wiberg MD, PhD^1,2^; Paul J. Kingham PhD^1^; and Peyman Kelk DDS, PhD^1*^

Affiliations:

1 Department of Integrative Medical Biology, Umeå University, 901 87 Umeå, Sweden

2 Department of Surgical & Perioperative Sciences, Section for Hand and Plastic Surgery, Umeå University, 901 87 Umeå, Sweden

Address for correspondence:

Dr. Peyman Kelk, DDS, PhD.

Department of Integrative Medical Biology, Umeå University, 901 87 Umeå, Sweden

Email: [peyman.kelk@umu.se](mailto:peyman.kelk@umu.se)

Tel: +46 90 786 63 81

**Supplementary Table 1.** Donor information

| **Donor Number** | **Age** | **Sex** |
| --- | --- | --- |
| 1 | 65 | Female |
| 2 | 19 | Male |
| 3 | 38 | Male |
| 4 | 17 | Female |

**Supplementary Table 2.** NanoString® metabolic panel

| Official Symbol | Accession | Target Sequence |
| --- | --- | --- |
| A2M | NM_000014.4 | TCCATCTCAATCCCTGTGAAGTCAGACATTGCTCCTGTCGCTCGGTTGCTCATCTATGCTGTTTTACCTACCGGGGACGTGATTGGGGATTCTGCAAAAT |
| AADAT | NM_016228.3 | ATAGCTCAGCTCCTAGCCCTTACTTGAGAGCATCCTTCTCTTCAGCTTCTCCAGAACAGATGGATGTGGCCTTCCAGGTATTAGCACAACTTATAAAAGA |
| AANAT | NM_001088.2 | GATCCGGCACTTCCTGACCCTATGTCCAGAGCTGTCCCTGGGCTGGTTCGAGGAGGGCTGCCTTGTGGCCTTCATCATCGGCTCGCTCTGGGACAAGGAG |
| ABL1 | NM_005157.3 | CTGCGTGAGCTATGTGGATTCCATCCAGCAAATGAGGAACAAGTTTGCCTTCCGAGAGGCCATCAACAAACTGGAGAATAATCTCCGGGAGCTTCAGATC |
| ACAA2 | NM_006111.2 | AGCTTACGGAGGCCTTCTGAAAGACTTCACTGCTACTGACTTGTCTGAATTTGCTGCCAAGGCTGCCTTGTCTGCTGGCAAAGTCTCACCTGAAACAGTT |
| ACACA | NM_198834.1 | GACATGAACACTGTACTGAACTACATCTTCTCTCACGCTCAAGTCACCAAGAAGAATCTTCTGGTCACAATGCTTATTGATCAGTTGTGTGGCCGGGACC |
| ACACB | NM_001093.3 | CAGCGGGATCCGCGGCTATATGAAAACAGTGGTGTTGGATCTCCTGAGAAGATACTTGCGTGTTGAGCACCATTTTCAGCAAGCCCACTACGACAAGTGT |
| ACADL | NM_001608.3 | TTGAAGATATACGGTTGCCAGCTAGTGCCCTACTTGGAGAAGAGAATAAAGGCTTCTATTACATCATGAAAGAGCTTCCACAGGAAAGGCTGTTAATTGC |
| ACAP2 | NM_012287.5 | TCAAGGATATGATCTGTTTAGTGAACTTGGACCCTACATGAAGGATCTTGGTGCACAGTTGGATCGACTGGTTGTGGATGCAGCAAAGGAGAAAAGAGAA |
| ACAT1 | NM_000019.3 | CATTGCAATTCAGGGAGCCATTGAAAAGGCAGGGATTCCAAAAGAAGAAGTGAAAGAAGCATACATGGGTAATGTTCTACAAGGAGGTGAAGGACAAGCT |
| ACAT2 | NM_005891.2 | TGTCGTTCTTATGAAGAAGTCAGAAGCTGATAAACGTGGGCTTACACCTTTAGCACGGATAGTTTCCTGGTCCCAAGTGGGTGTGGAGCCTTCCATTATG |
| ACMSD | NM_138326.2 | AGAGCTGGGCTTTCCCGGGGTCCAAATTGGCACCCACGTCAACGAGTGGGACCTGAACGCGCAGGAGCTCTTTCCTGTCTATGCGGCAGCCGAAAGGCTG |
| ACOT12 | NM_130767.2 | CAATGCTGCTGATGATAAGGAAAATCTCATCACGTTTCCCAGAATCCAACCCATTTCAAAGGATGATTTCAGACGCTATCGGGGAGCTATTGCACGCAAG |
| ACOX1 | NM_004035.5 | TTGCTAAATTGTCACAGTAGTAGGAAGTATAGGGAAACCTCTCAGCTGTGGCACTGTTGTAGCTTTGGAGTGCAGAGTGTAACTCTGGGACAATCAGATT |
| ACSF3 | NR_045667.2 | ACCCAAGGGAACGGCAGTCAGAGACTACAGTCCAGACGTTTGTGTCTAGGCCTTGCTGGTAGCTAAGGAAATGCCTTCTGTTGGGAAAAGGTCAAATTTA |
| ACY1 | NM_000666.2 | CGGCCGCTGGCGCCGGGACGGCCCTCACTGACGGTCTTCGGTCTCCGCCCCGACATCCGGCCTCGGCCACGTGGTGGGCGGACCGGGGCGGTCCTGAGCC |
| ADA | NM_000022.2 | TCCAAGAAGACCATGATCTCAATAGTCAGTTACTGATGCTCCTGAACCCTATGTGTCCATTTCTGCACACACGTATACCTCGGCATGGCCGCGTCACTTC |
| ADAL | NM_001012969.2 | GATGTTAGGTATTTGATAGCAGTTGACAGAAGAGGTGGCCCTTTAGTAGCCAAGGAGACTGTAAAACTTGCCGAGGAGTTCTTCCTTTCTACTGAGGGTA |
| ADH1A | NM_000667.3 | TGATTCCCAAAACCTCTCAATGAACCCTATGCTGCTACTGACTGGACGTACCTGGAAGGGAGCTATTCTTGGTGGCTTTAAAAGTAAAGAATGTGTCCCA |
| ADH1B | NM_000668.4 | ATAATCTTTAGTCATCGAATCCCAGTGGAGGGGACCCTTTTACTTGCCCTGAACATACACATGCTGGGCCATTGTGATTGAAGTCTTCTAACTCTGTCTC |
| ADH1C | NM_000669.3 | GATTCCCAGAACCTCTCAATAAACCCTATGCTGCTACTGACTGGACGCACGTGGAAAGGAGCTATTTTTGGAGGCTTTAAGAGTAAAGAATCTGTCCCCA |
| ADH4 | NM_000670.3 | ATTCAGATCATTGCTACCTCCCTGTGCCATACTGATGCCACTGTTATCGATTCTAAATTTGAGGGCCTAGCTTTCCCAGTGATCGTTGGCCATGAGGCTG |
| ADH6 | NM_000672.3 | GAAAGTTGCTACAGGATCTCCCTTTCTCAATAAATTCATCTGCGGTGGAGAAAATCAGCATGAGTACTACAGGCCAAGTCATCAGATGCAAAGCAGCCAT |
| ADH7 | NM_000673.3 | CAAGATGCTCACCTATGACCCGATGTTGCTCTTCACTGGACGCACATGGAAGGGATGTGTCTTTGGAGGTTTGAAAAGCAGAGATGATGTCCCAAAACTA |
| ADK | NM_001123.2 | AAAGTCGAATATCATGCTGGTGGCTCTACCCAGAATTCAATTAAAGTGGCTCAGTGGATGATTCAACAGCCACACAAAGCAGCAACATTTTTTGGATGCA |
| ADORA2A | NM_000675.3 | TGGCCATCGTCCTCTCCCACACCAATTCGGTTGTGAATCCCTTCATCTACGCCTACCGTATCCGCGAGTTCCGCCAGACCTTCCGCAAGATCATTCGCAG |
| AFMID | NM_001010982.4 | AGTGGAAAGCCTCATTTGAAGAGCTCCACGATGTGGACCACTTTGAAATTGTTGAGAATCTGACCCAGAAGGACAACGTGCTCACCCAGATTATCTTGAA |
| AGXT | NM_000030.2 | ACGCCCCTCCAGGGACCTCGCTCATCTCCTTCAGTGACAAGGCCAAAAAGAAGATGTACTCCCGCAAGACGAAGCCCTTCTCCTTCTACCTGGACATCAA |
| AGXT2 | NM_031900.3 | AGCACATGGGACTCCTCGTTGGCAGAGGCAGCATTTTTTCTCAGACATTTCGCATTGCGCCCTCAATGTGCATCACTAAACCAGAAGTTGATTTTGCAGT |
| AK3 | NM_016282.2 | GAGCTTATCAGATCGACACAGTGATTAACCTGAATGTGCCCTTTGAGGTCATTAAACAACGCCTTACTGCTCGCTGGATTCATCCCGCCAGTGGCCGAGT |
| AKR1C4 | NM_001818.2 | CCAGCCTTGGAAAGCTCACTGAAAAAACTTCAACTGGACTATGTTGACCTCTATCTTCTTCATTTCCCAATGGCTCTCAAGCCAGGTGAGACGCCACTAC |
| AKT1 | NM_001014431.1 | AGCCCAAGCACCGCGTGACCATGAACGAGTTTGAGTACCTGAAGCTGCTGGGCAAGGGCACTTTCGGCAAGGTGATCCTGGTGAAGGAGAAGGCCACAGG |
| AKT1S1 | NM_032375.3 | TAGTCGCTGGACAGCTCTTTTTTTGATTGGCTCAAATCCTGTAAAGGGCTTGACCAGTCTCTACATAGTCACCGTCCGCTTTTCCTGAGTTCTCCCTCCC |
| AKT2 | NM_001626.4 | AGGAGATGGAAGTGGCGGTCAGCAAGGCACGGGCTAAAGTGACCATGAATGACTTCGACTATCTCAAACTCCTTGGCAAGGGAACCTTTGGCAAAGTCAT |
| AKT3 | NM_005465.4 | GCCAGTTAATGAAAACAGAACGACCAAAGCCAAACACATTTATAATCAGATGTCTCCAGTGGACTACTGTTATAGAGAGAACATTTCATGTAGATACTCC |
| ALDH2 | NM_000690.2 | AAGCCCTATGTCATCTCCTACCTGGTGGATTTGGACATGGTCCTCAAATGTCTCCGGTATTATGCCGGCTGGGCTGATAAGTACCACGGGAAAACCATCC |
| ALDOA | NM_184041.2 | AGGCTGGCTTGCCCGCGCTCTTTCTTCCCTCGTGACAGTGGTGTGTGGTGTCGTCTGTGAATGCTAAGTCCATCACCCTTTCCGGCACACTGCCAAATAA |
| ALDOB | NM_000035.3 | CATCCAAAGAACAACTGCTGATTGAAACACCTCATTAGCTGAGTGTAGAGAAGTGCATCTTATGAAACAGTCTTAGCAGTGGTAGGTTGGGAAGGAGATA |
| ALOX12 | NM_000697.1 | TTACAGCCCGGAATGAGCAACTTGACTGGCCCTATGAATATCTGAAGCCCAGCTGCATAGAGAACAGTGTCACCATCTGAGCCCTAGAGTGACTCTACCT |
| ALOX15 | NM_001140.3 | CTGGATAAGGAAATTGAGATCCGGAATGCAAAGCTGGACATGCCCTACGAGTACCTGCGGCCCAGCGTGGTGGAAAACAGTGTGGCCATCTAAGCGTCGC |
| ALOX5 | NM_000698.2 | GTCAAGATCAGCAACACTATTTCTGAGCGGGTCATGAATCACTGGCAGGAAGACCTGATGTTTGGCTACCAGTTCCTGAATGGCTGCAACCCTGTGTTGA |
| AMDHD1 | NM_152435.2 | ATCTCAGCAATAGAGCTTCCCAGCAGCGTTCAAGACACATCATTTATACACAGGCACAGGGGCCTTCCTGAAATGGGTGCATTTTTACCAACTACAATCA |
| AMPD1 | NM_000036.2 | CCCACATTGATGAATACATTTCCTCATCTCCAACCTACCAGACCGTGCCTGATTTTCAGAGAGTGCAGATTACTGGTGACTATGCCTCTGGGGTTACAGT |
| AMPD2 | NM_004037.6 | CTTCTCTGTCTCTGTCTTGCATGTCTCCTACCATGTCACTGTCCCTGGGCCACCCAGTGAAAGCAAAGCCTGGGAATCTGCTCATTGTTGTTTGGGCTCA |
| AMPD3 | NM_000480.2 | CATTGGCCCAGGTAGGCTAATTGGTAGTTGTTCATTTCAGCCTCTGGATGGCTGGCTGCCTTAAACACAATCAATTTCAAAGCTCCATTTCATAAAGGGG |
| AOC1 | NM_001091.2 | CCACCATGGCCAAGAACACCGTGTTTCTCATCGAGATGCTGCTGCCCAAGAAGTACCATGTGCTGAGGTTTCTGGATAAAGGTGAAAGGCATCCTGTGCG |
| AOC3 | NM_001277731.1 | CCGTGGCCGAGGGCTTCCCTAGATGGTTCCCTTTGTTGCTGTCTGGCTTTCCCGAATCTTTTTAGGCCACCTCCAAGGACTCTAAAAGGGGGCTATTCCC |
| AOX1 | NM_001159.3 | GCGATAGTCAATTCAGGAATGAGAGTCTTTTTTGGAGAAGGGGATGGCATTATTAGAGAGTTATGCATCTCATATGGAGGCGTTGGTCCAGCCACCATCT |
| AP2S1 | NM_021575.2 | TGGCTGTGGGCAGAGGCCACCGTGTGTGTCCCGAGTAACCGTGCCGTTGTCGTGTGATGCCATAAGCGTCTGTGCGTGGAGTCCCCAATAAACCTGTGGT |
| APOA1 | NM_000039.1 | CTGGCCACTGTGTACGTGGATGTGCTCAAAGACAGCGGCAGAGACTATGTGTCCCAGTTTGAAGGCTCCGCCTTGGGAAAACAGCTAAACCTAAAGCTCC |
| APOA2 | NM_001643.1 | GAAGCTGCTCGCAGCAACTGTGCTACTCCTCACCATCTGCAGCCTTGAAGGAGCTTTGGTTCGGAGACAGGCAAAGGAGCCATGTGTGGAGAGCCTGGTT |
| APOA4 | NM_000482.3 | ACCTGTCTGTCTGTCTGTCCCAAAGAAGTTCTGGTATGAACTTGAGGACACATGTCCAGTGGGAGGTGAGACCACCTCTCAATATTCAATAAAGCTGCTG |
| APOB | NM_000384.2 | CACCAACTTCTTCCACGAGTCGGGTCTGGAGGCTCATGTTGCCCTAAAAGCTGGGAAGCTGAAGTTTATCATTCCTTCCCCAAAGAGACCAGTCAAGCTG |
| APOC2 | NM_000483.3 | TTGCTTCTCATGGATGGCACTGCTTTTCTGAGGACTCAAGGGCCAAGATGGAGGGGCTGACTCAGTCCAGCCAACATTTAATGAGCACCTACTTTATGTA |
| APOC3 | NM_000040.1 | CGAGCTCCTTGGGTCCTGCAATCTCCAGGGCTGCCCCTGTAGGTTGCTTAAAAGGGACAGTATTCTCAGTGCTCTCCTACCCCACCTCATGCCTGGCCCC |
| APOE | NM_000041.2 | GGGCTGCGTTGCTGGTCACATTCCTGGCAGGATGCCAGGCCAAGGTGGAGCAAGCGGTGGAGACAGAGCCGGAGCCCGAGCTGCGCCAGCAGACCGAGTG |
| APOM | NM_019101.2 | CAGCGCTTTCTCCTCTACAATCGCTCACCACATCCTCCCGAAAAGTGTGTGGAGGAATTCAAGTCCCTGACTTCCTGCCTGGACTCCAAAGCCTTCTTAT |
| APRT | NM_000485.2 | CTGCCCACAGGGAACACATTCCTTTGCTGGGGTTCAGCGCCTCTCCTGGGGCTGGAAGTGCCAAAGCCTGGGGCAAAGCTGTGTTTCAGCCACACTGAAC |
| AR | NM_000044.2 | AGACTGTGAGCCTAGCAGGGCAGATCTTGTCCACCGTGTGTCTTCTTCTGCACGAGACTTTGAGGCTGTCAGAGCGCTTTTTGCGTGGTTGCTCCCGCAA |
| ARF5 | NM_001662.2 | ATGGGCCTCACCGTGTCCGCGCTCTTTTCGCGGATCTTCGGGAAGAAGCAGATGCGGATTCTCATGGTTGGCTTGGATGCGGCTGGCAAGACCACAATCC |
| ARG1 | NM_000045.3 | TCAATGACTGAAGTGGACAGACTAGGAATTGGCAAGGTGATGGAAGAAACACTCAGCTATCTACTAGGAAGAAAGAAAAGGCCAATTCATCTAAGTTTTG |
| ARID1A | NM_006015.4 | AGCTCCCAGGGTTGCTAGAGCTCCTTGTAGAATATTTCCGACGATGCCTGATTGAGATCTTTGGCATTTTAAAGGAGTATGAGGTGGGTGACCCAGGACA |
| ARID1B | NM_020732.3 | TGCTACATTAGTTAGGTACGTTGGGGATCGCAAAAACCCAGTCTGTCGAGAAATGTCCATGGCGCTTTTATCGAACCTTGCCCAAGGGGACGCACTAGCA |
| ARID2 | NM_152641.2 | AGCTATTGGTTGGGCAGCAAAATGTTCAGTTGGTCCCAAGTGCAATGCCACCCTCAGGGGGAGTACAAACTGTGCCCATTTCGAACTTACAAATATTGCC |
| ARPC4 | NM_005718.4 | GACTTTGTGATCCACTTCATGGAGGAGATTGACAAGGAGATCAGTGAGATGAAGCTGTCAGTCAATGCCCGTGCCCGCATTGTGGCTGAAGAGTTCCTTA |
| ASCL1 | NM_004316.3 | ATAGTAACTCCCATCACCTCTAACACGCACAGCTGAAAGTTCTTGCTCGGGTCCCTTCACCTCCTCGCCCTTTCTTAAAGTGCAGTTCTTAGCCCTCTAG |
| ASH1L | NM_018489.2 | CTGTCCTGGAATCTTTGAAGCGCTATAGATTTGGAAAGGATGCTGTTGGAGAGCGATATAAGCATAAGGAAAAGCACCGTTGTCACATGTCCTGCCCTCA |
| ASL | NM_000048.3 | AGCTTTGGGGTGGCCGGTTTGTGGGTGCAGTGGACCCCATCATGGAGAAGTTCAACGCGTCCATTGCCTACGACCGGCACCTTTGGGAGGTGGATGTTCA |
| ASMT | NM_001171039.1 | AGGACCAGGCCTATCGCCTCCTTAATGACTACGCCAACGGCTTCATGGTGTCCCAGGTTCTCTTCGCCGCCTGCGAGCTGGGCGTGTTTGACCTTCTCGC |
| ASNS | NM_183356.2 | GGGTTACATATATTTTCACAAGGCTCCTTCTCCTGAAAAAGCCGAGGAGGAGAGTGAGAGGCTTCTGAGGGAACTCTATTTGTTTGATGTTCTCCGCGCA |
| ASPA | NM_000049.2 | CCTTCCCTCAAATATGCGACCACTCGTTCCATAGCCAAGTATCCTGTGGGTATAGAAGTTGGTCCTCAGCCTCAAGGGGTTCTGAGAGCTGATATCTTGG |
| ASPG | NM_001080464.2 | TGGTCATCCACGGCACCGACACCATGGCCTTTGCTGCCTCGATGCTGTCCTTCATGCTGGAGAACCTGCAGAAGACTGTCATCCTCACTGGGGCCCAGGT |
| ASS1 | NM_000050.4 | GCAAAATCAAACAAGGCCTGGGCTTGAAATTTGCTGAGCTGGTGTATACCGGTTTCTGGCACAGCCCTGAGTGTGAATTTGTCCGCCACTGCATCGCCAA |
| ATF4 | NM_001675.2 | TTTGAAGGAGTTCGACTTGGATGCCCTGTTGGGTATAGATGACCTGGAAACCATGCCAGATGACCTTCTGACCACGTTGGATGACACTTGTGATCTCTTT |
| ATF7 | NM_001130060.1 | CAGAGATTTACAAACGAGGACCACCTGGCAGTTCATAAACACAAGCATGAGATGACATTGAAATTTGGCCCAGCCCGAACTGACTCAGTCATCATTGCAG |
| ATF7IP | NM_018179.3 | TGATTTTGCATGTACCTGTTGCAGTATCCTCCCAGCCTCAGCTTCTACAGAGCCATCCAGGGACTTTGGTGACTAATCAACCATCTGGCAATGTTGAATT |
| ATG101 | NM_021934.4 | CGTGGAGGTGATGAATCGGCATGAGTACTTGCCCAAGATGCCCACACAGTCGGAGGTGGATAACGTGTTTGACACAGGCTTGCGGGACGTGCAGCCCTAC |
| ATG2B | NM_018036.5 | CTGTGCTTCAGCCCACTTGGGGAGAGTTCCTTGATCATCATAAAGAACAGCCAGTAAGAGGGTCAACATTTCCATCCAACCTAGTTCACCCAACACCTTT |
| ATOX1 | NM_004045.3 | GCTGAAGCTGTCTCTCGGGTCCTCAATAAGCTTGGAGGAGTTAAGTATGACATTGACCTGCCCAACAAGAAGGTCTGCATTGAATCTGAGCACAGCATGG |
| ATP5F1D | NM_001687.4 | CCCGGCTGCCGCCTCTGGCCCCAACCAGATGTCCTTCACCTTCGCCTCTCCCACGCAGGTGTTCTTCAACGGTGCCAACGTCCGGCAGGTGGACGTGCCC |
| ATP5ME | NM_007100.3 | TACCTAAAACCTCGGGCAGAAGAGGAGAGGAGGATAGCAGCAGAAGAGAAGAAGAAGCAGGATGAACTGAAACGGATTGCCAGAGAATTGGCAGAAGATG |
| ATP6V1F | NM_001198909.1 | AGGTGAGGCGCTTCTAGGTTGCTGGGGCTCTGCTGGTTAAGGAACAGGAAGCCTGACCATCTCCCTCCACTACCTCTTCCCTGTGCTGTTACACAGTGTC |
| ATXN7 | NM_001128149.2 | GAGTTGGAGAGAATGTAAGTCCTGACCTGAAGGTCTTTTGTGATGCATGTATAGGATTGCCCTGACACACACCCTCCTTTCTTGGGATTATACCAGCCAT |
| BAD | NM_004322.3 | CAGCACAGCGCTATGGCCGCGAGCTCCGGAGGATGAGTGACGAGTTTGTGGACTCCTTTAAGAAGGGACTTCCTCGCCCGAAGAGCGCGGGCACAGCAAC |
| BCL2 | NM_000657.2 | GTGAAGCAGAAGTCTGGGAATCGATCTGGAAATCCTCCTAATTTTTACTCCCTCTCCCCGCGACTCCTGATTCATTGGGAAGTTTCAAATCAGCTATAAC |
| BCL2A1 | NM_004049.2 | TCATGTGTCATAACTCAGTCAAGCTCAGTGAGCATTCTCAGCACATTGCCTCAACAGCTTCAAGGTGAGCCAGCTCAAGACTTTGCTCTCCACCAGGCAG |
| BCL2L1 | NM_138578.1 | CTAAGAGCCATTTAGGGGCCACTTTTGACTAGGGATTCAGGCTGCTTGGGATAAAGATGCAAGGACCAGGACTCCCTCCTCACCTCTGGACTGGCTAGAG |
| BHMT | NM_001713.2 | CGCAGCGGGAAGGCTCGCCTAGTCGGTCCGCATCCGTGTCGACCACCTGTCTGGACACCACGAAGATGCCACCCGTTGGGGGCAAAAAGGCCAAGAAGGG |
| BHMT2 | NM_001178005.1 | GCCAGACTTCTAAGGAGTAGTGAAAGAAAACCCTGAAATAATCGAACAGGAAAAAGTTGCCCTCAAGCCTGACCTGGAACCGTTCCTCACCTTCATCCTC |
| BIRC3 | NM_182962.1 | GTGAGACTCGCGCCCTCCGGCACGGAAAAGGCCAGGCGACAGGTGTCGCTTGAAAAGACTGGGCTTGTCCTTGCTGGTGCATGCGTCGTCGGCCTCTGGG |
| BLK | NM_001715.2 | AGCTTCTTGCTCCAATCAACAAGGCCGGCTCCTTTCTTATCAGAGAGAGTGAAACCAACAAAGGTGCCTTCTCCCTGTCTGTGAAGGATGTCACCACCCA |
| BRAF | NM_004333.3 | GTACCTGCAAGGTGTGGAGTTACAGTCCGAGACAGTCTAAAGAAAGCACTGATGATGAGAGGTCTAATCCCAGAGTGCTGTGCTGTTTACAGAATTCAGG |
| BRCA1 | NM_007294.3 | TCTTCTGAAGATACCGTTAATAAGGCAACTTATTGCAGTGTGGGAGATCAAGAATTGTTACAAATCACCCCTCAAGGAACCAGGGATGAAATCAGTTTGG |
| BRCA2 | NM_000059.3 | GGGGACAGATTTGTGACCGGCGCGGTTTTTGTCAGCTTACTCCGGCCAAAAAAGAACTGCACCTCTGGAGCGGACTTATTTACCAAGCATTGGAGGAATA |
| BRCC3 | NM_024332.3 | TGTGGGCTGGTATCATTCCCATCCTCATATAACTGTTTGGCCTTCACATGTTGATGTTCGCACACAAGCCATGTACCAGATGATGGATCAAGGCTTTGTA |
| BRIP1 | NM_032043.1 | GATGTGCAAAGCCTGGGATATAGAAGAACTTGTCAGCCTGGGGAAGAAACTAAAGGCCTGTCCATATTACACAGCCCGAGAACTAATACAAGATGCTGAC |
| BTK | NM_000061.1 | TGATCTGGTTCAGAAATATCACCCTTGCTTCTGGATCGATGGGCAGTATCTCTGCTGCTCTCAGACAGCCAAAAATGCTATGGGCTGCCAAATTTTGGAG |
| BUB1 | NM_004336.3 | GAAGATAAAGAAAATGTGGTAGCAAAACAGTGTACCCAGGCGACTTTGGATTCTTGTGAGGAAAACATGGTGGTGCCTTCAAGGGATGGAAAATTCAGTC |
| BUB1B | NM_001211.4 | GAGTCTTCTGTACCACAACGAAGCACACTAGCTGAACTAAAGAGCAAAGGGAAAAAGACAGCAAGAGCTCCAATCATCCGTGTAGGAGGTGCTCTCAAGG |
| CA12 | NM_001218.3 | CCATATTCTTCCCCGAATCACACAGACAGTTTCTGACAGGCGCAACTCCTCCATTTTCCTCCCGCAGGTGAGAACCCTGTGGAGATGAGTCAGTGCCATG |
| CA9 | NM_001216.2 | CAGGTCCCAGGACTGGACATATCTGCACTCCTGCCCTCTGACTTCAGCCGCTACTTCCAATATGAGGGGTCTCTGACTACACCGCCCTGTGCCCAGGGTG |
| CAB39 | NM_001130849.1 | TCACTGAAGCTTCTCGGTGAACTACTACTAGATAGACACAACTTCACAATTATGACAAAATACATCAGTAAACCTGAGAACCTCAAATTAATGATGAACC |
| CACNA1A | NM_001127221.1 | AGTTTGAGAAAGATTGTCGAGGCAAATACCTCCTCTACGAGAAGAATGAGGTGAAGGCGCGAGACCGGGAGTGGAAGAAGTATGAATTCCATTACGACAA |
| CACNA1E | NM_000721.2 | CAGGACGACCATTCTGAGCCAGTGAGTCTATTGCATGGTGTTTTGAGAAAGGGAATTTTAGTGACACCTTATGCCATTTCAAGAGGAGAAATGCAGGCTG |
| CACNB4 | NM_000726.3 | GTGAAATTGGCTTCATTCCAAGTCCACTCAGATTGGAGAACATACGGATCCAGCAAGAACAAAAAAGAGGACGTTTTCACGGAGGGAAATCAAGTGGAAA |
| CACNG2 | NM_006078.3 | TTTCTTGACACCGGCATTTCCTGAGGTTGAGCTCTCTGGGGAGTTCTAGAGAATGGCTAGTAAGGCTTTTTGAGCTTGCACATCTCACCTGCCTTTCCTG |
| CACNG3 | NM_006539.2 | TGGTTGCCGTGCACATCTATATTGAAAAACATCAGCAGTTACGAGCCAAATCCCACTCGGAGTTCCTGAAGAAATCTACTTTTGCCCGCCTCCCACCCTA |
| CACNG7 | NM_031896.3 | TGGCTCCGCCCACAGACTCCCTTATTTCAATGGCCGCGCCCTCTTTTCCCGACCTCTCCTTTTCATTGGTCCCTCTCACTCCCAAATGACTCCTCCCCTT |
| CAD | NM_004341.3 | TTCCTCGATGGGACCTTAGCAAGTTCCTGCGAGTCAGCACAAAGATTGGGAGCTGCATGAAGAGCGTTGGTGAAGTCATGGGCATTGGGCGTTCATTTGA |
| CARD11 | NM_032415.2 | TTGAAAATCGGCCCAAGAAGGAGCAGGTTCTGGAACTGGAGCGGGAGAATGAAATGCTGAAGACCAAAAACCAGGAGCTGCAGTCCATCATCCAGGCCGG |
| CAT | NM_001752.2 | ATGCTTCAGGGCCGCCTTTTTGCCTATCCTGACACTCACCGCCATCGCCTGGGACCCAATTATCTTCATATACCTGTGAACTGTCCCTACCGTGCTCGAG |
| CBL | NM_005188.2 | GTTGTGGTAAGGATGCAGGGTATTTCGCAGAACCCAGGACGGGAAGTGCCTTTGGTTCTTGGGTGGAGCTGGAACTGCAGAGCTTTGCACCTAGTCCTTT |
| CBR4 | NM_032783.4 | AAGATATGGTATCTCAGCTTCATACTAACCTCTTGGGTTCCATGCTGACCTGTAAAGCTGCCATGAGGACTATGATTCAACAACAGGGAGGGTCTATTGT |
| CCL13 | NM_005408.2 | CCAGAATTATATGAAACACCTGGGCCGGAAAGCTCACACCCTGAAGACTTGAACTCTGCTACCCCTACTGAAATCAAGCTGGAGTACGTGAAATGACTTT |
| CCL19 | NM_006274.2 | GACCTCAGCCAAGATGAAGCGCCGCAGCAGTTAACCTATGACCGTGCAGAGGGAGCCCGGAGTCCGAGTCAAGCATTGTGAATTATTACCTAACCTGGGG |
| CCL4 | NM_002984.2 | GAAGCTTCCTCGCAACTTTGTGGTAGATTACTATGAGACCAGCAGCCTCTGCTCCCAGCCAGCTGTGGTATTCCAAACCAAAAGAAGCAAGCAAGTCTGT |
| CCL5 | NM_002985.2 | CCAAGTGTGTGCCAACCCAGAGAAGAAATGGGTTCGGGAGTACATCAACTCTTTGGAGATGAGCTAGGATGGAGAGTCCTTGAACCTGAACTTACACAAA |
| CCNA1 | NM_003914.3 | TTGTGCCTTGCCTGAGTGAGCTTCATAAAGCGTACCTTGATATACCCCATCGACCTCAGCAAGCAATTAGGGAGAAGTACAAGGCTTCAAAGTACCTGTG |
| CCNA2 | NM_001237.2 | CGGGACAAAGCTGGCCTGAATCATTAATACGAAAGACTGGATATACCCTGGAAAGTCTTAAGCCTTGTCTCATGGACCTTCACCAGACCTACCTCAAAGC |
| CCNB2 | NM_004701.2 | AGGTTGATGTTGAACAGCACACTTTAGCCAAGTATTTGATGGAGCTGACTCTCATCGACTATGATATGGTGCATTATCATCCTTCTAAGGTAGCAGCAGC |
| CCND1 | NM_053056.2 | TTGAACACTTCCTCTCCAAAATGCCAGAGGCGGAGGAGAACAAACAGATCATCCGCAAACACGCGCAGACCTTCGTTGCCCTCTGTGCCACAGATGTGAA |
| CD14 | NM_000591.2 | GCCCAAGCACACTCGCCTGCCTTTTCCTGCGAACAGGTTCGCGCCTTCCCGGCCCTTACCAGCCTAGACCTGTCTGACAATCCTGGACTGGGCGAACGCG |
| CD163 | NM_004244.4 | CATCTGTGATTCGGACTTCTCTCTGGAAGCTGCCAGCGTTCTATGCAGGGAATTACAGTGTGGCACAGTTGTCTCTATCCTGGGGGGAGCTCACTTTGGA |
| CD180 | NM_005582.2 | TTCACCCAACTCCAAGAATTGGATCTGACAGCAACTCACTTGAAAGGGTTACCCTCTGGGATGAAGGGTCTGAACTTGCTCAAGAAATTAGTTCTCAGTG |
| CD19 | XM_011545981.1 | CACCCCAAGGGGCCTAAGTCATTGCTGAGCCTAGAGCTGAAGGACGATCGCCCGGCCAGAGATATGTGGGTAATGGAGACGGGTCTGTTGTTGCCCCGGG |
| CD209 | NM_001144899.1 | TAGAGCTTGTTTTTCTGGCCCATCCTTGGAGCTTTATGAGTGAGCTGGTGTGGGATGCCTTTGGGGGTGGACTTGTGTTCCAAGAATCCACTCTCTCTTC |
| CD244 | NM_001166663.1 | CTCCAGGCGCTGGGGCTTTCTCAGTGGCCTTGTCAGCTCACAGCAGGCGTTAACAGCCTCTAATTGAGGAAACTGTGGCTGGACAGGTTGCAAGGCAGTT |
| CD247 | NM_000734.3 | GGCATAAAAAACATGTGGTTACACAGTGTGAATAAAGTGCTGCGGAGCAAGAGGAGGCCGTTGATTCACTTCACGCTTTCAGCGAATGACAAAATCATCT |
| CD27 | NM_001242.4 | GCTGCCAGATGTGTGAGCCAGGAACATTCCTCGTGAAGGACTGTGACCAGCATAGAAAGGCTGCTCAGTGTGATCCTTGCATACCGGGGGTCTCCTTCTC |
| CD274 | NM_014143.3 | AAGGATACTTCTGAACAAGGAGCCTCCAAGCAAATCATCCATTGCTCATCCTAGGAAGACGGGTTGAGAATCCCTAATTTGAGGGTCAGTTCCTGCAGAA |
| CD276 | NM_001024736.1 | TACATTTCTTAGGGACACAGTACACTGACCACATCACCACCCTCTTCTTCCAGTGCTGCGTGGACCATCTGGCTGCCTTTTTTCTCCAAAAGATGCAATA |
| CD28 | NM_001243078.1 | GCTGCTCCTGTACCTTGGAGGTCCATTCACATGGGAAAGTATTTTGGAATGTGTCTTTTGAAGAGAGCATCAGAGTTCTTAAGGGACTGGGTAAGGCCTG |
| CD36 | NM_000072.3 | AGCCAAGGAAAATGTAACCCAGGACGCTGAGGACAACACAGTCTCTTTCCTGCAGCCCAATGGTGCCATCTTCGAACCTTCACTATCAGTTGGAACAGAG |
| CD3D | NM_000732.4 | TATCTACTGGATGAGTTCCGCTGGGAGATGGAACATAGCACGTTTCTCTCTGGCCTGGTACTGGCTACCCTTCTCTCGCAAGTGAGCCCCTTCAAGATAC |
| CD3E | NM_000733.2 | AAGTAACAGTCCCATGAAACAAAGATGCAGTCGGGCACTCACTGGAGAGTTCTGGGCCTCTGCCTCTTATCAGTTGGCGTTTGGGGGCAAGATGGTAATG |
| CD3G | NM_000073.2 | GAACTAAATGCAGCCACCATATCTGGCTTTCTCTTTGCTGAAATCGTCAGCATTTTCGTCCTTGCTGTTGGGGTCTACTTCATTGCTGGACAGGATGGAG |
| CD4 | NM_000616.4 | TGGCAGGCGGAGAGGGCTTCCTCCTCCAAGTCTTGGATCACCTTTGACCTGAAGAACAAGGAAGTGTCTGTAAAACGGGTTACCCAGGACCCTAAGCTCC |
| CD40LG | NM_000074.2 | GCATTTGATTTATCAGTGAAGATGCAGAAGGGAAATGGGGAGCCTCAGCTCACATTCAGTTATGGTTGACTCTGGGTTCCTATGGCCTTGTTGGAGGGGG |
| CD6 | NM_001254751.1 | AACCCTGGACACTGCATTACAGACCCGCCATCCCTGGGCCCTCAGTATCACCCGAGGAGCAACAGTGAGTCGAGCACCTCTTCAGGGGAGGATTACTGCA |
| CD63 | NM_001780.4 | GTCATCATCGCAGTGGGTGTCTTCCTCTTCCTGGTGGCTTTTGTGGGCTGCTGCGGGGCCTGCAAGGAGAACTATTGTCTTATGATCACGTTTGCCATCT |
| CD68 | NM_001251.2 | ACCGGTCCATCTTGCTGCCTCTCATCATCGGCCTGATCCTTCTTGGCCTCCTCGCCCTGGTGCTTATTGCTTTCTGCATCATCCGGAGACGCCCATCCGC |
| CD84 | NM_001184879.1 | TCTGCTAGAACAGTGCCGTGCTTTTCCACAGAAGGTTAGACCCTGAAAGAGATGGCTCAGCACCACCTATGGATCTTGCTCCTTTGCCTGCAAACCTGGC |
| CD8A | NM_001768.6 | AGCTCAGGGCTCTTTCCTCCACACCATTCAGGTCTTTCTTTCCGAGGCCCCTGTCTCAGGGTGAGGTGCTTGAGTCTCCAACGGCAAGGGAACAAGTACT |
| CD8B | NM_172099.2 | TCAGCTGAGTGTGGTTGATTTCCTTCCCACCACTGCCCAGCCCACCAAGAAGTCCACCCTCAAGAAGAGAGTGTGCCGGTTACCCAGGCCAGAGACCCAG |
| CDA | NM_001785.2 | AATCTTCAAAGGGTGCAACATAGAAAATGCCTGCTACCCGCTGGGCATCTGTGCTGAACGGACCGCTATCCAGAAGGCCGTCTCAGAAGGGTACAAGGAT |
| CDC20 | NM_001255.2 | GGAACATCAGAAAGCCTGGGCTTTGAACCTGAACGGTTTTGATGTAGAGGAAGCCAAGATCCTTCGGCTCAGTGGAAAACCACAAAATGCGCCAGAGGGT |
| CDCA5 | NM_080668.3 | ACCCAGTGCGGCTGCAGTCAGAAAGCCCATCGTCTTAAAGAGGATCGTGGCCCATGCTGTAGAGGTCCCAGCTGTCCAATCACCTCGCAGGAGCCCTAGG |
| CDCA8 | NM_018101.2 | TCCCTGTTTACTGAAGACCAAATACTGGTTTGGAGACAACTTCCATGTCTTGCTCTTCTACCTCCCTAGTTAGTGGAAATTTGGATAAGGGAACTGTAGG |
| CDK9 | NM_001261.2 | GGTGTTCGACTTCTGCGAGCATGACCTTGCTGGGCTGTTGAGCAATGTTTTGGTCAAGTTCACGCTGTCTGAGATCAAGAGGGTGATGCAGATGCTGCTT |
| CEACAM3 | NM_001815.3 | GCCTTCCTGTGGGGGCCGTCGCCGGCATCGTGACCGGGGTCCTGGTCGGAGTGGCGCTGGTGGCCGCGCTGGTGTGTTTCCTGCTCCTTGCCAAAACTGG |
| CENPA | NM_001042426.1 | CACTTTGAGCAGTTGCCTGGAAGGCTGGGCATTTCCATCATATAGACCTCTGCCCTTCAGAGTAGCCTCACCATTAGTGGCAGCATCATGTAACTGAGTG |
| CHMP2A | NM_014453.3 | GGAGCTGGACCGCGAGCGACAGAAACTAGAGACCCAGGAGAAGAAAATCATTGCAGACATTAAGAAGATGGCCAAGCAAGGCCAGATGGATGCTGTTCGC |
| CHMP6 | NM_024591.4 | GTATTGAGTTCACCCAGATCGAAATGAAAGTGATGGAGGGGCTGCAGTTTGGAAATGAGTGTCTGAACAAGATGCACCAGGTGATGTCCATTGAAGAGGT |
| CLOCK | NM_004898.2 | AGCCGGAAGCATGGTCCAGATTCCATCTAGTATGCCACAAAACAGCACCCAGAGTGCTGCAGTAACTACATTCACTCAGGACAGGCAGATAAGATTTTCT |
| CLSPN | NM_022111.2 | GGAAAATCTTGAAGCGCAAGTGAAACCTTGCTTAGAGCTGAGTCTTCAGTCTGGAAACTCTACAGACTTTACCACTGACAGAAAGAGTTCCAAAAAGCAC |
| CMKLR1 | NM_004072.2 | TTCAACGTCTTCCTCCCAATCCATATCACCTATGCCGCCATGGACTACCACTGGGTTTTCGGGACAGCCATGTGCAAGATCAGCAACTTCCTTCTCATCC |
| COL4A1 | NM_001845.4 | TGGGCTTAAGTTTTCAAGGACCAAAAGGTGACAAGGGTGACCAAGGGGTCAGTGGGCCTCCAGGAGTACCAGGACAAGCTCAAGTTCAAGAAAAAGGAGA |
| COL6A1 | NM_001848.2 | CCTCTGTCCCCATAGCTGGTTTTTCCCACCAATCCTCACCTAACAGTTACTTTACAATTAAACTCAAAGCAAGCTCTTCTCCTCAGCTTGGGGCAGCCAT |
| COL6A3 | NM_004369.3 | AGAGCAAGCGAGACATTCTGTTCCTCTTTGACGGCTCAGCCAATCTTGTGGGCCAGTTCCCTGTTGTCCGTGACTTTCTCTACAAGATTATCGATGAGCT |
| COPS6 | NM_006833.4 | CAGATTTTTATGATCAATGCAACGACGTGGGGCTCATGGCCTACCTCGGCACCATCACCAAAACGTGCAACACCATGAACCAGTTTGTGAACAAGTTCAA |
| COX14 | NM_001257133.1 | CCCTGTTGGAAGAAAGTGCCCATGGTTTCTCTGGTTCTGCCAGTTTGACAGTTTATGGAGGCTTTTGAATCGTAATAGCAATGTGAGGGTGAGGTACACC |
| COX4I1 | NM_001318797.1 | GGCAGAATGTTGGCTACCAGGGTATTTAGCCTAGTTGGCAAGCGAGCAATTTCCACCTCTGTGTGTGTACGAGCTCATGTGTTGTGAAGAGCGAAGACTT |
| COX5A | NM_004255.3 | ACTTCAACAAGCCAGATATAGATGCCTGGGAATTGCGTAAAGGGATAAACACACTTGTTACCTATGATATGGTTCCAGAGCCCAAAATCATTGATGCTGC |
| COX5B | NM_001862.2 | CAGGCACCAGGGAAGACCCTAATTTAGTCCCCTCCATCTCCAACAAGAGAATAGTAGGCTGCATCTGTGAAGAGGACAATACCAGCGTCGTCTGGTTTTG |
| COX6A1 | NM_004373.2 | ATCAGGACCAAGCCGTTTCCCTGGGGAGATGGTAACCATACTCTATTCCATAACCCTCATGTGAATCCACTTCCAACTGGCTACGAAGATGAATAAAGAG |
| COX6B1 | NM_001863.4 | AACCAGACTAGAAACTGCTGGCAGAACTACCTGGACTTCCACCGCTGTCAGAAGGCAATGACCGCTAAAGGAGGCGATATCTCTGTGTGCGAATGGTACC |
| COX7B | NM_001866.2 | CAGAGCCACCAGAAACGTACACCTGATTTTCATGACAAATACGGTAATGCTGTATTAGCTAGTGGAGCCACTTTCTGTATTGTTACATGGACATATGTAG |
| COX7C | NM_001867.2 | TCTGCGCCTTTCGCAGAGCTTCCAGCAGCGGTATGTTGGGCCAGAGCATCCGGAGGTTCACAACCTCTGTGGTCCGTAGGAGCCACTATGAGGAGGGCCC |
| COX8A | NM_004074.2 | GGCCAGCGCAGCCATTTTGGCTTCCTGACCTTGGGCTACGGCTGACCGTTTTTTGTGGTGTACTCCGTGCCATCATGTCCGTCCTGACGCCGCTGCTGCT |
| CPA3 | NM_001870.2 | ACCCACCACGTAGCTGCTAATATGATGGTGGATTTCCGAGTTAGTGAGAAGGAATCCCAAGCCATCCAGTCTGCCTTGGATCAAAATAAAATGCACTATG |
| CPS1 | NM_001875.3 | AGACGATGGATTCTGTTGAACTATGGGGTCCCACACTGCACTATTAATTCCACCCACTGTAAGGGCAAGGACACCATTCCTTCTACATATAAGAAAAAAG |
| CPT1A | NM_001876.3 | CAGGCCTATTTTGGACGTGGGAAAAATAAGCAGTCTCTTGATGCTGTGGAGAAAGCAGCGTTCTTCGTGACGTTAGATGAAACTGAAGAAGGATACAGAA |
| CREB3L3 | NM_001271995.1 | CTGAAGAAACTCCAGGCCATTGTGGTGCAGTCCACCAGCAAGTCAGCCCAGACAGGCACCTGTGTCGCAGTCCTGTTGCTGTCCTTTGCCCTCATCATCC |
| CS | NM_004077.2 | CATATGCACAGGGTATCAGCCGAACCAAGTACTGGGAGTTGATTTATGAAGACTCTATGGATCTAATCGCAAAGCTACCTTGTGTTGCAGCAAAGATCTA |
| CSF3R | NM_000760.3 | AGGCCCTTTCAGCTCTATGAGATCATCGTGACTCCCTTGTACCAGGACACCATGGGACCCTCCCAGCATGTCTATGCCTACTCTCAAGAAATGGCTCCCT |
| CTCF | NM_001191022.1 | ATTGGTTCGGCATCGTCGTTACAAACACACCCACGAGAAGCCATTCAAGTGTTCCATGTGCGATTACGCCAGTGTAGAAGTCAGCAAATTAAAACGTCAC |
| CTLA4 | NM_005214.3 | AGTCTGTGCGGCAACCTACATGATGGGGAATGAGTTGACCTTCCTAGATGATTCCATCTGCACGGGCACCTCCAGTGGAAATCAAGTGAACCTCACTATC |
| CTPS1 | NM_001301237.1 | ATATGATCGCTTGCTGGAGACCTGCTCTATTGCCCTTGTGGGCAAATACACGAAGTTCTCAGACTCCTATGCCTCTGTCATTAAGGCTCTGGAGCATTCT |
| CTSA | NM_001127695.1 | TGCCACAATGGGACATGTGCAACTTTCTGGTAAACTTACAGTACCGCCGTCTCTACCGAAGCATGAACTCCCAGTATCTGAAGCTGCTTAGCTCACAGAA |
| CTSD | NM_001909.3 | GAAGCCGGCGGCCCAAGCCCGACTTGCTGTTTTGTTCTGTGGTTTTCCCCTCCCTGGGTTCAGAAATGCTGCCTGCCTGTCTGTCTCTCCATCTGTTTGG |
| CTSL | NM_001912.4 | TGTGGGGCCCATTTCTGTTGCTATTGATGCAGGTCATGAGTCCTTCCTGTTCTATAAAGAAGGCATTTATTTTGAGCCAGACTGTAGCAGTGAAGACATG |
| CTSS | NM_004079.3 | ATGACAACGGCTTTCCAGTACATCATTGATAACAAGGGCATCGACTCAGACGCTTCCTATCCCTACAAAGCCATGGATCAGAAATGTCAATATGACTCAA |
| CTSW | NM_001335.3 | TGCACCGAGGGAGCAATACCTGTGGCATCACCAAGTTCCCGCTCACTGCCCGTGTGCAGAAACCGGATATGAAGCCCCGAGTCTCCTGCCCTCCCTGAAC |
| CTSZ | NM_001336.3 | GGACACCACATATATAAACCATGTCGTTTCTGTGGCTGGGTGGGGCATCAGTGATGGGACTGAGTACTGGATTGTCCGGAATTCATGGGGTGAACCATGG |
| CXCL9 | NM_002416.2 | CCATCTCCCATGAAGAAAGGGAACGGTGAAGTACTAAGCGCTAGAGGAAGCAGCCAAGTCGGTTAGTGGAAGCATGATTGGTGCCCAGTTAGCCTCTGCA |
| CXCR6 | NM_006564.1 | ACCATGAAGACTATGGGTTCAGCAGTTTCAATGACAGCAGCCAGGAGGAGCATCAAGACTTCCTGCAGTTCAGCAAGGTCTTTCTGCCCTGCATGTACCT |
| CYBB | NM_000397.3 | TTTGAAGCATGAAAAAAGAGGGTTGGAGGTGGAGAATTAACCTCCTGCCATGACTCTGGCTCATCTAGTCCTGCTCCTTGTGCTATAAAATAAATGCAGA |
| CYP1A1 | NM_000499.3 | GTATCAGTGACCAATGTCATCTGTGCCATTTGCTTTGGCCGGCGCTATGACCACAACCACCAAGAACTGCTTAGCCTAGTCAACCTGAATAATAATTTCG |
| CYP1A2 | NM_000761.3 | ACTTCGACCCTTACAATCAGGTGGTGGTGTCAGTGGCCAACGTCATTGGTGCCATGTGCTTCGGACAGCACTTCCCTGAGAGTAGCGATGAGATGCTCAG |
| CYP1B1 | NM_000104.3 | AGAACTTTGATCCAGCTCGATTCTTGGACAAGGATGGCCTCATCAACAAGGACCTGACCAGCAGAGTGATGATTTTTTCAGTGGGCAAAAGGCGGTGCAT |
| CYP4A11 | NM_000778.3 | AGTCTGCCTGCCCTTCTCTCTCTCACCTTTCTCCAGGCTCCCTACCTGCTTGTCTACCTGTCTCCTACCCACCTGTATCTCTTGTTGGGAGAAAAGCTGA |
| CYP4A22 | NM_001010969.3 | TCAGGAACGGGTGAAGACATTCCCAAGTGCCTGTCCTTATTGGATATGGGGAGGCAAAGTTCGTGTCCAGCTCTATGACCCTGACTATATGAAGGTGATT |
| CYP8B1 | NM_004391.2 | TTTCCATCTGCCCTGGGAGGTTCTTTGCACTCAGTGAGGTGAAGCTCTTTATCCTGCTTATGGTCACACACTTTGACTTAGAGTTGGTGGACCCTGACAC |
| D2HGDH | NM_001287249.1 | GAGGAAGGACAACACGGGCTATGACCTGAAGCAGCTGTTCATCGGGTCGGAGGGCACTTTGGGGATCATCACCACGGTGTCCATCTTGTGTCCACCCAAG |
| DAO | NM_001917.4 | GAATGCAAGAATTATTGGTGAACGAACTGGCTTCCGGCCAGTACGCCCCCAGATTCGGCTAGAAAGAGAACAGCTTCGCACTGGACCTTCAAACACAGAG |
| DCK | NM_000788.2 | GCTGCCCGTCTTTCTCAGCCAGCTCTGAGGGGACCCGCATCAAGAAAATCTCCATCGAAGGGAACATCGCTGCAGGGAAGTCAACATTTGTGAATATCCT |
| DDC | NM_000790.3 | GTTGCCACCCTGGGGACCACAACATGCTGCTCCTTTGACAATCTCTTAGAAGTCGGTCCTATCTGCAACAAGGAAGACATATGGCTGCACGTTGATGCAG |
| DEPTOR | NM_022783.3 | AGCATGTGTCCAACAAGCACCCATTTGTGGACAGCAATCTTCTCTACCAGTTCAGAATGAACTTCCGGCGGAGGCGAAGACTGATGGAGCTGCTCAATGA |
| DERA | NM_015954.2 | GGAGATGAGTGGCTGAAGCCAGAACTCTTTCGAATAGGTGCCAGTACTCTGCTCTCGGACATTGAGAGGCAGATTTACCATCATGTGACTGGAAGATATG |
| DGLUCY | NM_001102369.2 | GAGCCAAGTTACACCCTGTTTAACCCTGCCTTCAAAGGGACGACTCTGTAAGATTCTCTGCTACTTATTCAAGTTGACACGATGCCCTTCACACTCCACC |
| DGUOK | NM_080916.2 | TTGTAAAGAATCTGTAACCAATACCATGAAGTTCAGGCTGTGATCTGGGCTCCCTGACTTTCTGAAGCTAGAAAAATGTTGTGTCTCCCAACCACCTTTC |
| DMGDH | NM_013391.2 | CCAGGCCAGGACACTCAGTACAGGCCAAGTTTTCGCCGCACAAACTGGTTTGAGCCTGTGGGCTCGGAGTATAAACAGGTTATGCAAAGAGTAGCGGTAA |
| DTL | NM_016448.2 | AACATGCAAAGGTCATCAATGCAGCCTCAAGTCAGTTGCCTTTTCTAAGTTTGAGAAAGCTGTATTCTGTACGGGTGGAAGAGATGGCAACATTATGGTC |
| DUOX1 | NM_175940.1 | TGAGCCCACACCTCACCTCTGTTCTTCCTATTTCTGGCTGCCTCAGCCTTCTCTGATTTCCCACCTCCCAACCTTGTTCCAGGTGGCCATAGTCAGTCAC |
| DUOX2 | NM_014080.4 | AGGCACATCGTGTGTGTGGCAATCTTCTCGGCCATCTGTGTTGGCGTGTTTGCAGATCGTGCTTACTACTATGGCTTTGCCTCGCCACCCTCGGACATTG |
| ECHS1 | NM_004092.3 | GTTCGCTGTCCCGCCTGGCGTCCCTTCGCCTCGGGTGCTAACTTTGAGTACATCATCGCAGAAAAAAGAGGGAAGAATAACACCGTGGGGTTGATCCAAC |
| EEA1 | NM_003566.3 | CATAAACAAGCCCAGGAGAATTTGCATGACCAGGTACAAGAGCAGAAGGCACATCTTAGAGCTGCACAAGACCGTGTCCTTTCCCTAGAAACTAGTGTCA |
| EFNA4 | NM_005227.2 | GTGCTCCCTGCCCTTTGGCCATGTTCAATTCTCAGAGAAGATTCAGCGCTTCACACCCTTCTCCCTCGGCTTTGAGTTCTTACCTGGAGAGACTTACTAC |
| EGFR | NM_201282.1 | ACATCCTGCCGGTGGCATTTAGGGGTGACTCCTTCACACATACTCCTCCTCTGGATCCACAGGAACTGGATATTCTGAAAACCGTAAAGGAAATCACAGG |
| EHHADH | NM_001166415.1 | CATTGAACCACGTACCATTAGCCAGGATGAGATCCTTGAACGCTGCTTATATTCACTTATCAATGAAGCATTCCGTATCTTGGGAGAAGGGATAGCTGCT |
| EME1 | XM_011524392.1 | ATGGCTCTAAAGAAGTCATCACCCTCACTGGATTCTGGTGATAGTGACTCTGAGGAGTTGCCAACATTTGCCTTTCTGAAGAAGGAACCATCTTCAACAA |
| ENO1 | NM_001428.2 | TTCTCGCCTCACTTTCCACCAAGTGTCTAGAGTCATGTGAGCCTCGTGTCATCTCCGGGGTGGCCACAGGCTAGATCCCCGGTGGTTTTGTGCTCAAAAT |
| ENO3 | NM_001976.4 | ATGAGGATCGAGGAGGCTCTTGGGGACAAGGCAATCTTTGCTGGACGCAAGTTCCGTAACCCGAAGGCCAAGTGAGAAGCTGGAGGCTCCAGGACTCCAC |
| EOMES | NM_005442.2 | ATCCCATGCCCTGGGGTATTACCCAGACCCAACCTTTCCTGCAATGGCAGGGTGGGGAGGTCGAGGTTCTTACCAGAGGAAGATGGCAGCTGGACTACCA |
| EPC1 | NM_025209.2 | AAAGGTGTATTGGATTTGCACGAAGACGGGTTGGGCGCGGTGGAAGGGTCTTACTGGACAGAGCTCATTCAGACTATGACAGTGTGTTTCACCATCTGGA |
| ERCC6 | NM_001277058.1 | TCAAGTCAAACTCAGGAGCAAGACTGTTTACAGAGTCAACCTGTCAGTAATAATGAAGAAATGGCAATCAAGCAAGAAAGTGGTGGTGATGGGGAGGTGG |
| ERN1 | NM_001433.2 | TACATGGGTAAAAAGCAGGACATCTGGTATGTTATTGACCTCCTGACCGGAGAGAAGCAGCAGACTTTGTCATCGGCCTTTGCAGATAGTCTCTGCCCAT |
| EXO1 | NM_003686.3 | GCCAGAGCCAGTGGGCTGAGCAAGAAGCCGGCAAGCATCCAGAAGAGAAAGCATCATAATGCCGAGAACAAGCCGGGGTTACAGATCAAACTCAATGAGC |
| EZH2 | NM_001203247.1 | ACACAGAAACAGCTCTAGACAACAAACCTTGTGGACCACAGTGTTACCAGCATTTGGAGGGAGCAAAGGAGTTTGCTGCTGCTCTCACCGCTGAGCGGAT |
| FABP5 | NM_001444.1 | GCTTTGATGAATACATGAAGGAGCTAGGAGTGGGAATAGCTTTGCGAAAAATGGGCGCAATGGCCAAGCCAGATTGTATCATCACTTGTGATGGTAAAAA |
| FAH | NM_000137.1 | CATGACGAGCCCTACACATTTGACATCAACCTCTCTGTTAACCTGAAAGGAGAAGGAATGAGCCAGGCGGCTACCATATGCAAGTCCAATTTTAAGTACA |
| FAHD1 | NM_031208.3 | CATAACCTTGGAAGAAGGAGATATTATCTTGACTGGGACGCCAAAGGGAGTTGGACCGGTTAAAGAAAACGATGAGATCGAGGCTGGCATACACGGGCTG |
| FAM30A | NR_026800.2 | ACAGTTCTGAAGTCAAAGGCTGATGTCCTGTTTCTCTTTCCCTCTGTGACCGACTCCCTTCCCAGTGGTAACAAGTACCCACAGCTTGGTTTGAATTTCT |
| FANCA | NM_000135.2 | CTGAGAAGAACTGTGGAGCCTGAAAAAATGCCGCAGGTCACGGTTGATGTACTGCAGAGAATGCTGATTTTTGCACTTGACGCTTTGGCTGCTGGAGTAC |
| FANCD2 | NM_033084.3 | GGGAGAGAGTCAGAATCAACTAGCTGTGGATCAAATAGCTTTCCAAAAGAAGCTCTTTCAGACCCTGAGGAGACACCCTTCCTATCCCAAAATAATAGAA |
| FANCI | NM_001113378.1 | TGAGTGGGGAAGAATGTAAGAAACAGTTGATTAACACCCTGTGTTCTGGCAGGTGGGATCAGCAATATGTAATCCAACTCACCTCCATGTTCAAGGATGT |
| FASLG | NM_000639.1 | TCCATGCCTCTGGAATGGGAAGACACCTATGGAATTGTCCTGCTTTCTGGAGTGAAGTATAAGAAGGGTGGCCTTGTGATCAATGAAACTGGGCTGTACT |
| FASN | NM_004104.4 | GAGGTGCTTGGCTACGCACGGTCGCTTCCTGGAAATTGGCAAATTCGACCTTTCTCAGAACCACCCGCTCGGCATGGCTATCTTCCTGAAGAACGTGACA |
| FBP1 | NM_000507.3 | AGTCATCCTTTGCCACGTGTGTTCTCGTGTCAGAAGAAGATAAACACGCCATCATAGTGGAACCGGAGAAAAGGGGTAAATATGTGGTCTGTTTTGATCC |
| FCAR | NM_002000.2 | TGCTGAGATTATAGGCATGAGCCACCACGCCTGGCCAGATGCATGTTCAAACCAATCAAATGGTGTTTTCTTATGCAGGACTGATCGATTTGCACCCACC |
| FCRL2 | NM_001159488.1 | CCTCTTCTGTCTTCGAAGGAGACAGCATCGTTCTGAAATGCCAGGGAGAACAGAACTGGAAAATTCAGAAGATGGCTTACCATAAGGATAACAAAGAGTT |
| FDX1 | NM_004109.4 | AGACAGATCACGGTTGGGCTGCCAAATCTGTTTGACAAAATCTATGGACAATATGACTGTTCGAGTGCCTGAAACAGTGGCTGATGCCAGACAATCCATT |
| FDXR | NM_004110.3 | CCCGTGCAGTGCCCACGGGAGACATGGAAGACCTCCCTTGTGGGCTGGTGCTCAGCAGCATTGGGTATAAGAGCCGCCCTGTCGACCCAAGCGTGCCCTT |
| FGF1 | NM_033137.1 | AAATGAGGAATGTTTGTTCCTGGAAAGGCTGGAGGAGAACCATTACAACACCTATATATCCAAGAAGCATGCAGAGAAGAATTGGTTTGTTGGCCTCAAG |
| FH | NM_000143.2 | GGTGAACTAAAGGTGCCAAATGATAAGTATTATGGCGCCCAGACCGTGAGATCTACGATGAACTTTAAGATTGGAGGTGTGACAGAACGCATGCCAACCC |
| FLT1 | NM_002019.4 | AAGAAATGGCAAACAATTCTGCAGTACTTTAACCTTGAACACAGCTCAAGCAAACCACACTGGCTTCTACAGCTGCAAATATCTAGCTGTACCTACTTCA |
| FLT3 | NM_004119.2 | GAAATTGAATATGAAAACCAAAAAAGGCTGGAAGAAGAGGAGGACTTGAATGTGCTTACATTTGAAGATCTTCTTTGCTTTGCATATCAAGTTGCCAAAG |
| FNIP1 | NM_001008738.2 | ACAATTTGTAATCTTTACACGATGCCACGAATTGGAGAACCTGTCTGGCTTACAATGATGTCGGGGACTCCAGAAAAGAACCACCTTTGCTATCGTTTCA |
| FNIP2 | NM_020840.1 | TACTTTATGTCCTGACCTACTTTCTCCGTTGCTCTGAGCTACAAGAGAACCAGCTGACCTGGAGTGGCAATCATGGTGAAGGTGACCAAGTTTTAAATGG |
| FOLH1 | NM_004476.1 | ACCACCTCCTCCAGGATATGAAAATGTTTCGGATATTGTACCACCTTTCAGTGCTTTCTCTCCTCAAGGAATGCCAGAGGGCGATCTAGTGTATGTTAAC |
| FOLH1B | NM_153696.2 | CTGAGGATTTTTAGAGCTTATAGTAGCAAAAAGAAAAGGGAAATTCTCTCTGAGATGTCCTTTTTTGTAGGCCTAATGACAAAAGGTTGAAGATAAAGTT |
| FOLR1 | NM_000802.2 | GATACCTGGAAATCCCTGCCCTGTTCAGCCCCACAGCTCCCAACTATTTGGTTCCTGCTCCATGGTCGGGCCTCTGACAGCCACTTTGAATAAACCAGAC |
| FOLR3 | NM_000804.2 | GCGCTGGTGGGAGGACTGTCGCACCTCCTACACCTGCAAAAGCAACTGGCACAAAGGCTGGAATTGGACCTCAGGGATTAATGAGTGTCCGGCCGGGGCC |
| FOXM1 | NM_202002.1 | CAATTCGCCATCAACAGCACTGAGAGGAAGCGCATGACTTTGAAAGACATCTATACGTGGATTGAGGACCACTTTCCCTACTTTAAGCACATTGCCAAGC |
| FOXP3 | NM_014009.3 | GGGCCATCCTGGAGGCTCCAGAGAAGCAGCGGACACTCAATGAGATCTACCACTGGTTCACACGCATGTTTGCCTTCTTCAGAAACCATCCTGCCACCTG |
| FPR1 | NM_002029.3 | GCCATGGGAGGACATTGGCCTTTCGGCTGGTTCCTGTGCAAATTCGTCTTTACCATAGTGGACATCAACTTGTTCGGAAGTGTCTTCCTGATCGCCCTCA |
| FTCD | NM_006657.2 | TGACCTACGGGCGGCGCCAATTCCAGTCCCTGGACACGACGATGCGGCGCCTGATCCCGCCCTTCCGCGAGGCTTCGGCCAAGCTAACCACGCTGGTGGA |
| G6PC | NM_000151.2 | GATGTGGAGTCTTCGGTGTTTAAAGTCAACAACCATGCCAGGGATTGAGGAGGACTACTATTTGAAGCAATGGGCACTGGTATTTGGAGCAAGTGACATG |
| GABARAP | NM_007278.1 | CTGGACAAAAAGAAATACCTGGTGCCTTCTGATCTCACAGTTGGTCAGTTCTACTTCTTGATCCGGAAGCGAATTCATCTCCGAGCTGAGGATGCCTTGT |
| GAD1 | NM_000817.2 | CAAAGGACCAACAGCCTGGAAGAGAAGAGTCGCCTTGTGAGTGCCTTCAAGGAGAGGCAATCCTCCAAGAACCTGCTTTCCTGTGAAAACAGCGACCGGG |
| GADL1 | NM_207359.2 | CTTGGAGATACACAGTAGATTGCAGCCCTTCTGATGAGAAATAGGGAATACTCCCAGTCCAGGCCCAGCAAAACCAAAATGCTAAGCAATGAATATTAAG |
| GAPDH | NM_001256799.1 | GAACGGGAAGCTTGTCATCAATGGAAATCCCATCACCATCTTCCAGGAGCGAGATCCCTCCAAAATCAAGTGGGGCGATGCTGGCGCTGAGTACGTCGTG |
| GAPDHS | NM_014364.4 | ACCCCGCTACTCCTCCTCCTAAGATGGTGTCTGTGGCCCGGGAGCTGACTGTGGGCATCAATGGATTTGGACGCATCGGTCGCCTGGTCCTGCGCGCCTG |
| GAPVD1 | NM_001282679.1 | TTCATACTCTGGCTCATCACCTCAAGCAGGAACGCTTATATGTAAACTCTGAGAAACAGCTCATTCAGAGGCTCAATGCAGATGTACTTAAGACAGCTGA |
| GART | NM_000819.3 | GCCCAACAGCAGAAGCGGCTCAGTTAGAGTCCAGCAAAAGGTTTGCCAAAGAGTTTATGGACAGACATGGAATCCCAACCGCACAATGGAAGGCTTTCAC |
| GATM | NM_001482.2 | TCTCTTCTTACAACGAATGGGACCCCTTAGAGGAAGTGATAGTGGGCAGAGCAGAAAACGCCTGTGTTCCACCGTTCACCATCGAGGTGAAGGCCAACAC |
| GBA | NM_001005742.2 | CAATTGGGTGCGTAACTTTGTCGACAGTCCCATCATTGTAGACATCACCAAGGACACGTTTTACAAACAGCCCATGTTCTACCACCTTGGCCACTTCAGC |
| GCDH | NM_000159.2 | CTGGAGCGGGTGGACAGTGGCTACAGGTCGGCGATGAGTGTCCAGTCCTCCCTCGTCATGCACCCTATCTATGCCTATGGCAGCGAGGAACAGCGGCAGA |
| GCK | NM_000162.4 | CTTCAAGGAGCGGTTCCATGCCAGCGTGCGCAGGCTGACGCCCAGCTGCGAGATCACCTTCATCGAGTCGGAGGAGGGCAGTGGCCGGGGCGCGGCCCTG |
| GCLC | NM_001498.2 | CTCAAGTGGGGCGATGAGGTGGAATACATGTTGGTATCTTTTGATCATGAAAATAAAAAAGTCCGGTTGGTCCTGTCTGGGGAGAAAGTTCTTGAAACTC |
| GDA | NM_001242506.2 | TAGAGTGAAGCCCATAGTGACACCACGTTTTTCCCTCTCCTGCTCTGAGACTTTGATGGGTGAACTGGGCAACATTGCTAAAACCCGTGATTTGCACATT |
| GLRX | NM_002064.2 | CACCAACCACACTAACGAGATTCAAGATTATTTGCAACAGCTCACGGGAGCAAGAACGGTGCCTCGAGTCTTTATTGGTAAAGATTGTATAGGCGGATGC |
| GLS | NM_014905.3 | AGTCTGGAGGAAAGGTTGCAGATTATATTCCTCAACTGGCCAAATTCAGTCCCGATTTGTGGGGTGTGTCTGTTTGTACAGTAGATGGACAGAGGCATTC |
| GLS2 | NM_013267.2 | CCCTGGATGATGCTGTGCAGTTCAACCATCTGGAGGTGGTCAAACTGCTTCAAGATTACCAGGACTCCTACACACTCTCTGAAACTCAGGCTGAGGCAGC |
| GLUD1 | NM_005271.2 | GTGTCCCAGAAAAAAACCACTTGGGCTCCCTGTTTGGAGTCTGGCTGGCTCTGAGCATTGCCAATGGCCCCTACTCACCTGACTTTGTATCCTCTCCTTT |
| GLUL | NM_001033044.2 | GGTTTAGAGATAAGAGTTGGCTGGTCAACTTGAGCATGTTACTGACAGAGGGGGTATTGGGGTTATTTTCTGGTAGGAATAGCATGTCACTAAAGCAGGC |
| GLYAT | NM_005838.3 | TTCACATAAACCATGGAAATCCATTCAATCTGAAGGCTGTGGTGGACAAGTGGCCTGATTTTAATACAGTGGTTGTCTGCCCTCAGGAGCAGGATATGAC |
| GLYCTK | NR_026700.1 | CAGCCTCAGCTTGTAGGCCTCGTTGGATGGATGTCCTTTCTGTCTTTGGACAGCCCTTAGCCACATACTGCACTTCTGGTTGAGCTGAAGTTTGTCCCAC |
| GMPR | NM_006877.3 | CCATGTTTACAGCAATTCATAAGCATTACTCCCTGGATGACTGGAAGCTCTTTGCCACAAATCACCCAGAATGCCTGCAGAATGTAGCCGTGAGTTCAGG |
| GMPR2 | NM_001002001.2 | GCTAAGCGCCATGCCTCATATTGACAACGATGTGAAACTGGACTTCAAGGATGTCCTTTTGAGGCCCAAACGCAGTACCCTTAAGTCTCGAAGTGAGGTG |
| GMPS | NM_003875.2 | TAAGGATGGCCACCACCACTATGAAGGAGCTGTTGTCATTCTGGATGCTGGTGCTCAGTACGGGAAAGTCATAGACCGAAGAGTGAGGGAACTGTTCGTG |
| GNG12 | NM_018841.3 | ATAAAGGTTTCGAAGGCATCAGCGGACCTCATGTCCTACTGTGAGGAACATGCCAGGAGTGACCCTTTGCTGATAGGAATACCAACTTCAGAAAACCCTT |
| GNLY | NM_012483.2 | TGCCGGCTCCTCGCTTCCTCGATCCAGAATCCACTCTCCAGTCTCCCTCCCCTGACTCCCTCTGCTGTCCTCCCCTCTCACGAGAATAAAGTGTCAAGCA |
| GNS | NM_002076.3 | ATGGATGGGATGTCCTTATTGCCCATTTTGAGAGGTGCCAGTAACTTGACCTGGCGATCAGATGTCCTGGTGGAATACCAAGGAGAAGGCCGTAACGTCA |
| GOT1 | NM_002079.2 | GTCCTCACCAACCTGGGAGAATCACAATGCTGTGTTTTCCGCTGCTGGTTTTAAAGACATTCGGTCCTATCGCTACTGGGATGCAGAGAAGAGAGGATTG |
| GOT2 | NM_002080.2 | GGAGAGTAGGAAACTGTACTTTATCTCGGCATCCTCTTGAATGATAGTGCAAGTTTCTCCAGTTGGGATGTTGTCTCTGCCCGGTTGGACCTCCTCCCTT |
| GPI | NM_000175.2 | CAGTGCTCAAGTGACCTCTCACGACGCTTCTACCAATGGGCTCATCAACTTCATCAAGCAGCAGCGCGAGGCCAGAGTCCAATAAACTCGTGCTCATCTG |
| GPS1 | NM_004127.4 | GGCCCGGGACTACTGCACCAGCGCCAAACACGTCATCAACATGTGCCTCAATGTCATCAAGGTCAGCGTCTACTTGCAGAATTGGTCTCATGTGCTCAGC |
| GPT | NM_005309.2 | ACGCACAGGCTATGGGGCAGAGGCCCATCACCTTCCTGCGCCAGGTCTTGGCCCTCTGTGTTAACCCTGATCTTCTGAGCAGCCCCAACTTCCCTGACGA |
| GPX1 | NM_000581.2 | GGTTTTCATCTATGAGGGTGTTTCCTCTAAACCTACGAGGGAGGAACACCTGATCTTACAGAAAATACCACCTCGAGATGGGTGCTGGTCCTGTTGATCC |
| GPX4 | NM_001039847.1 | CAGGGAGTAACGAAGAGATCAAAGAGTTCGCCGCGGGCTACAACGTCAAATTCGATATGTTCAGCAAGATCTGCGTGAACGGGGACGACGCCCACCCGCT |
| GRAP2 | NM_001291826.1 | TATCTGCAGCACCACCATTTCCACCAGGAACGCCGAGGAGGCAGCCTTGACATAAATGATGGGCATTGTGGCACCGGCTTGGGCAGTGAAATGAATGCGG |
| GRIN1 | NM_000832.5 | TTCAAGAGAGTGCTGATGTCTTCCAAGTATGCGGATGGGGTGACTGGTCGCGTGGAGTTCAATGAGGATGGGGACCGGAAGTTCGCCAACTACAGCATCA |
| GSK3B | NM_002093.2 | ACTGATTATACCTCTAGTATAGATGTATGGTCTGCTGGCTGTGTGTTGGCTGAGCTGTTACTAGGACAACCAATATTTCCAGGGGATAGTGGTGTGGATC |
| GTSE1 | NM_016426.5 | GATGAAGTCTTCTTCGGACCCTTTGGACATAAAGAAAGATGTATTGCTGCCAGCTTGGAATTAAATAATCCGGTTCCCGAACAGCCTCCGTTGCCCACAT |
| GUSB | NM_000181.3 | CCGATTTCATGACTGAACAGTCACCGACGAGAGTGCTGGGGAATAAAAAGGGGATCTTCACTCGGCAGAGACAACCAAAAAGTGCAGCGTTCCTTTTGCG |
| GYS2 | NM_021957.3 | GCTGGAATTGGACTGATCCTTTCTCGAGCCAGGAAACTTCCTATTGCCACAATATTTACAACCCACGCTACACTACTTGGGAGGTATCTCTGTGCAGCAA |
| GZMA | NM_006144.2 | AGACCCTACATGGTCCTACTTAGTCTTGACAGAAAAACCATCTGTGCTGGGGCTTTGATTGCAAAAGACTGGGTGTTGACTGCAGCTCACTGTAACTTGA |
| GZMB | NM_004131.3 | ACACTACAAGAGGTGAAGATGACAGTGCAGGAAGATCGAAAGTGCGAATCTGACTTACGCCATTATTACGACAGTACCATTGAGTTGTGCGTGGGGGACC |
| GZMH | NM_033423.3 | AAAAAAGGGACACCTCCAGGAGTCTACATCAAGGTCTCACACTTCCTGCCCTGGATAAAGAGAACAATGAAGCGCCTCTAACAGCAGGCATGAGACTAAC |
| H6PD | NM_004285.3 | AGCATTGTAACTCAGTCATGGGAGCTGCCTCTTTGGAAGTGCAGATTTATTCCTGTAATAATCCTGCCTGCTTTTACCTCTCGTCCACTGACCAGCAAGT |
| HAAO | NM_012205.2 | CAGCTGGAGGGAGACATGGTTCTCCGAGTCCTGGAGCAAGGGAAACACCGGGATGTGGTCATTCGGCAGGGAGAGATATTCCTCCTGCCTGCCAGGGTGC |
| HACD2 | NM_198402.2 | CTAATATGGGCAGTAACACATAGCGTCAAAGAGGTACAGAGTGAAGACAGTGTCCTCCTGTTTGTTATTGCATGGACGATCACGGAAATCATCCGTTACT |
| HADH | NM_001184705.2 | ATCTTTGCCAGCAACACTTCCTCCTTGCAGATTACAAGCATAGCTAATGCCACCACCAGACAAGACCGATTCGCTGGCCTCCATTTCTTCAACCCAGTGC |
| HDC | NM_002112.3 | GCAGGCACTGCCTTCCTGTGCCCCGAGTTCCGGGGGTTTCTGAAGGGGATTGAGTATGCCGACTCCTTCACCTTTAATCCTTCCAAGTGGATGATGGTGC |
| HERC1 | NM_003922.3 | CCAACAAGTTTTATGCCTCAAAGGACCACAGTTGCCAGACTTTGAACGTGAGTCTCTTTCAAGTGATGAGCAGGACCACTATTTGGATGCCCTTCTTAGC |
| HEXA | NM_000520.4 | CGCTTTCCTCACCGGGGCTTGCTGTTGGATACATCTCGCCATTACCTGCCACTCTCTAGCATCCTGGACACTCTGGATGTCATGGCGTACAATAAATTGA |
| HEXB | NM_000521.3 | CAGATTACGAGGAATTCGAGTCCTGCCAGAATTTGATACCCCTGGGCATACACTATCTTGGGGAAAAGGTCAGAAAGACCTCCTGACTCCATGTTACAGT |
| HIF1A | NM_001530.2 | ATGGATGATGACTTCCAGTTACGTTCCTTCGATCAGTTGTCACCATTAGAAAGCAGTTCCGCAAGCCCTGAAAGCGCAAGTCCTCAAAGCACAGTTACAG |
| HIF3A | NM_152796.2 | ACCTACTGTGACGACAGGATTGCAGAAGTGGCTGGCTATAGTCCCGATGACCTGATCGGCTGTTCCGCCTACGAGTACATCCACGCGCTGGACTCCGATG |
| HJURP | NM_018410.3 | AACAATACGACAGGGCCATGGAGAGAACCGTCAGAGGGAGATTGAAATCCGATTTGATCAGCTTCATCGGGAATATTGCCTGAGTCCCAGGAACCAGCCT |
| HK1 | NM_000188.2 | CTGTGGCCTGGCATCGCATCGTGGTGTGTCAATGCCACAAAATCGTGTGTCCGTGGAACCAGTCCTAGCCGCGTGTGACAGTCTTGCATTCTGTTTGTCT |
| HK2 | NM_000189.4 | TCAGAATCAGGTTGACAGTCCCTTGCTGACATGGCTTTGCTTTGTGTAAATACAGTGGATCTCAATCTTCGGGGTGTGATGAATAGCGAATCATCTCAAA |
| HK3 | NM_002115.1 | ACTTTGCTGCCCACTGCCTGTCTGAGTTCCTGGATGCGCAGCCTGTGAACAAACAGGGTCTGCAGCTTGGCTTCAGCTTCTCTTTCCCTTGTCACCAGAC |
| HLA-A | NM_002116.5 | GGAAGAGCTCAGATAGAAAAGGAGGGAGTTACACTCAGGCTGCAAGCAGTGACAGTGCCCAGGGCTCTGATGTGTCCCTCACAGCTTGTAAAGTGTGAGA |
| HLA-C | NM_002117.4 | AGCTGGGAGCCATCTTCCCAGCCCACCATCCCCATCATGGGCATCGTTGCTGGCCTGGCTGTCCTGGTTGTCCTAGCTGTCCTTGGAGCTGTGGTCACCG |
| HLA-DQA1 | NM_002122.3 | GGCGGTGGCCTGAGTTCAGCAAATTTGGAGGTTTTGACCCGCAGGGTGCACTGAGAAACATGGCTGTGGCAAAACACAACTTGAACATCATGATTAAACG |
| HLA-DRB1 | NM_002124.3 | TTCGGCAACTGCAGAAAATGTCCTCCCTTGTGGCTTCCTCAGCTCCTGCCCTTGGCCTGAAGTCCCAGCATTGATGGCAGCGCCTCATCTTCAACTTTTG |
| HLA-E | NM_005516.5 | CTATGTGTCTTAGGGGACTCTGGCTTCTCTTTTTGCAAGGGCCTCTGAATCTGTCTGTGTCCCTGTTAGCACAATGTGAGGAGGTAGAGAAACAGTCCAC |
| HMOX1 | NM_002133.2 | CAAGGACCAGAGCCCCTCACGGGCACCAGGGCTTCGCCAGCGGGCCAGCAACAAAGTGCAAGATTCTGCCCCCGTGGAGACTCCCAGAGGGAAGCCCCCA |
| HNF4A | NM_178850.1 | CCTTGCAGAGCATCACCTGGCAGATGATCGAGCAGATCCAGTTCATCAAGCTCTTCGGCATGGCCAAGATTGACAACCTGTTGCAGGAGATGCTGCTGGG |
| HPD | NM_002150.2 | CGATCACCTGGTGAAACACGGTGACGGAGTGAAGGACATTGCGTTCGAGGTGGAAGATTGTGACTACATCGTGCAGAAAGCACGGGAACGGGGCGCCAAA |
| HPRT1 | NM_000194.1 | TGTGATGAAGGAGATGGGAGGCCATCACATTGTAGCCCTCTGTGTGCTCAAGGGGGGCTATAAATTCTTTGCTGACCTGCTGGATTACATCAAAGCACTG |
| HRAS | NM_005343.2 | AGTACATGCGCACCGGGGAGGGCTTCCTGTGTGTGTTTGCCATCAACAACACCAAGTCTTTTGAGGACATCCACCAGTACAGGGAGCAGATCAAACGGGT |
| HSD11B1 | NM_181755.1 | GCCTACTACTACTATTCTGCAAACGAGGAATTCAGACCAGAGATGCTCCAAGGAAAGAAAGTGATTGTCACAGGGGCCAGCAAAGGGATCGGAAGAGAGA |
| HSD17B8 | NM_014234.3 | AGGACTCTAAGTTCCCAGGATACAAAAGGGGTGGCAGTGTATGGTTCAGGAATGCTGAATATGGGAAGCAGGGGTGCTTGTGACCCTAATAAATTCCAAG |
| HSF1 | XM_011517006.1 | CCCCTGATGCTGAACGACAGTGGCTCAGCACATTCCATGCCCAAGTATAGCCGGCAGTTCTCCCTGGAGCACGTCCACGGCTCGGGCCCCTACTCGGCCC |
| HSF2 | NM_001135564.1 | TGAGTCCAGGCTTTCTGAATTAAAAAGTGAGAATGAGTCCCTTTGGAAGGAGGTGTCAGAATTACGAGCAAAGCATGCACAACAGCAACAAGTTATTCGA |
| HSPA2 | NM_021979.3 | AAGATCCTCGACAAGTGTCAGGAGGTGATCAACTGGCTCGACCGAAACCAGATGGCAGAGAAAGATGAGTATGAACACAAGCAGAAAGAGCTCGAAAGAG |
| HSPA4 | NM_002154.3 | TAGAGTGGAGCCACCACTTCGTAGTGTTTTGGAACAAACCAAGTTAAAGAAAGAAGATATTTATGCAGTGGAGATAGTTGGTGGTGCTACACGAATCCCT |
| HSPE1 | NM_002157.2 | GGGTGGAGAGATTCAACCAGTTAGCGTGAAAGTTGGAGATAAAGTTCTTCTCCCAGAATATGGAGGCACCAAAGTAGTTCTAGATGACAAGGATTATTTC |
| ICOS | NM_012092.2 | AACTCTGGCACCCAGGCATGAAGCACGTTGGCCAGTTTTCCTCAACTTGAAGTGCAAGATTCTCTTATTTCCGGGACCACGGAGAGTCTGACTTAACTAC |
| IDH1 | NM_005896.3 | TATGATTTAGGCATAGAGAATCGTGATGCCACCAACGACCAAGTCACCAAGGATGCTGCAGAAGCTATAAAGAAGCATAATGTTGGCGTCAAATGTGCCA |
| IDH2 | NM_002168.2 | CACCGGCTCATTGATGACATGGTGGCTCAGGTCCTCAAGTCTTCGGGTGGCTTTGTGTGGGCCTGCAAGAACTATGACGGAGATGTGCAGTCAGACATCC |
| IDH3A | NM_005530.2 | TGAAAAACATAATGGAAATAAAACAGGATATTGACATAATAGCACAAAATGACACTCTTCTAAAACTAAATGGGCACAAGAGAATTTTCCTGGGAAAGTT |
| IDH3B | NM_001258384.1 | CATGAGAGTGCAAGGGGTGTGATTGAGTGTTTGAAGATTGTCACACGAGCCAAGTCTCAGCGGATTGCAAAGTTCGCCTTTGACTATGCCACCAAGAAGG |
| IDH3G | NM_004135.2 | CTCATGCTGCATGTCAAGTCCGTCTTCAGGCACGCATGTGTACCAGTGGACTTTGAAGAGGTGCACGTGAGTTCCAATGCTGATGAAGAGGACATTCGCA |
| IDNK | NM_001001551.3 | CATACCGCTCAATGACCAGGACCGGATTCCATGGCTCTGTAACTTGCATGACATTTTACTAAGAGATGTAGCCTCGGGACAGCGTGTGGTTCTAGCCTGT |
| IDO1 | NM_002164.5 | ATTATAAGATGCTCTGAAAACTCTTCAGACACTGAGGGGCACCAGAGGAGCAGACTACAAGAATGGCACACGCTATGGAAAACTCCTGGACAATCAGTAA |
| IDO2 | NM_194294.2 | GTTCTGCCTGGGATCATCCAGGAAGGATCTCAGCCCTATTCATGTTTCTGCTCTACAGAGCACTATATTCTCCTTGTTGAGAGCTGTTGGCTTCACAAAG |
| IFNG | NM_000619.2 | ATACTATCCAGTTACTGCCGGTTTGAAAATATGCCTGCAATCTGAGCCAGTGCTTTAATGGCATGTCAGACAGAACTTGAATGTGTCAGGTGACCCTGAT |
| IL10 | NM_000572.2 | AAGGATCAGCTGGACAACTTGTTGTTAAAGGAGTCCTTGCTGGAGGACTTTAAGGGTTACCTGGGTTGCCAAGCCTTGTCTGAGATGATCCAGTTTTACC |
| IL2 | NM_000586.2 | AGGATGCAACTCCTGTCTTGCATTGCACTAAGTCTTGCACTTGTCACAAACAGTGCACCTACTTCAAGTTCTACAAAGAAAACACAGCTACAACTGGAGC |
| IL21R | NM_021798.2 | CGTGTTTGTGGTCAACAGATGACAACAGCCGTCCTCCCTCCTAGGGTCTTGTGTTGCAAGTTGGTCCACAGCATCTCCGGGGCTTTGTGGGATCAGGGCA |
| IL2RA | NM_000417.1 | CTTGGTAAGAAGCCGGGAACAGACAACAGAAGTCATGAAGCCCAAGTGAAATCAAAGGTGCTAAATGGTCGCCCAGGAGACATCCGTTGTGCTTGCCTGC |
| IL4 | NM_000589.2 | GACACTCGCTGCCTGGGTGCGACTGCACAGCAGTTCCACAGGCACAAGCAGCTGATCCGATTCCTGAAACGGCTCGACAGGAACCTCTGGGGCCTGGCGG |
| IL4I1 | NM_152899.1 | CCGGCGCTCTGGCAAACCGAAAAGGATGACTGGACGGTCCCTTATGGCCGCATCTACTTTGCCGGCGAGCACACCGCCTACCCGCACGGCTGGGTGGAGA |
| IL6 | NM_000600.3 | GGCACTGGCAGAAAACAACCTGAACCTTCCAAAGATGGCTGAAAAAGATGGATGCTTCCAATCTGGATTCAATGAGGAGACTTGCCTGGTGAAAATCATC |
| IL7 | NM_000880.3 | AGGGTCCTGGGAGTGACTATGGGCGGTGAGAGCTTGCTCCTGCTCCAGTTGCGGTCATCATGACTACGCCCGCCTCCCGCAGACCATGTTCCATGTTTCT |
| IMPDH1 | NM_000883.3 | GTGAGGTGATGACGCCAAGGATTGAACTGGTGGTGGCTCCAGCAGGTGTGACGTTGAAAGAGGCAAATGAGATCCTGCAGCGTAGCAAGAAAGGGAAGCT |
| IMPDH2 | NM_000884.2 | AGCCGCTTGGTGGGCATCATCTCCTCCAGGGACATTGATTTTCTCAAAGAGGAGGAACATGACTGTTTCTTGGAAGAGATAATGACAAAGAGGGAAGACT |
| INMT | NM_006774.4 | TATACTAAGCCTTACAGCTATCTTAGATGCGATCTGACTCCTGTGTGACTGTGGAGCACCCAGGGACGTGGTTTTAGAGTCTACCTAATATGTTAAGGAC |
| INSR | NM_000208.2 | CACCCGGGGTTCTGTCCGCATCGAGAAGAACAATGAGCTCTGTTACTTGGCCACTATCGACTGGTCCCGTATCCTGGATTCCGTGGAGGATAATTACATC |
| IRF1 | NM_002198.2 | TTAGTCGAGGCAAGACGTGCGCCCGAGCCCCGCCGAACCGAGGCCACCCGGAGCCGTGCCCAGTCCACGCCGGCCGTGCCCGGCGGCCTTAAGAACCCGG |
| IRF4 | NM_002460.1 | GGGCACTGTTTAAAGGAAAGTTCCGAGAAGGCATCGACAAGCCGGACCCTCCCACCTGGAAGACGCGCCTGCGGTGCGCTTTGAACAAGAGCAATGACTT |
| ITCH | NM_001257138.1 | GAGGTGACAAAGAGCCAACAGAGACAATAGGAGACTTGTCAATTTGTCTTGATGGGCTACAGTTAGAGTCTGAAGTTGTTACCAATGGTGAAACTACATG |
| ITGA1 | NM_181501.1 | AAGTGGCAAGACTATAAGGAAAGAGTATGCACAACGTATTCCATCAGGTGGGGATGGTAAGACACTGAAATTTTTTGGCCAGTCTATCCACGGAGAAATG |
| ITGA11 | NM_012211.3 | CCTGAAAAAGTTTTACATTGGCCCAGGGCAGATCCAGGTTGGAGTTGTGCAGTATGGCGAAGATGTGGTGCATGAGTTTCACCTCAACGACTACAGGTCT |
| ITGAM | NM_000632.3 | GCCCTCCGAGGGTGTCCTCAAGAGGATAGTGACATTGCCTTCTTGATTGATGGCTCTGGTAGCATCATCCCACATGACTTTCGGCGGATGAAGGAGTTTG |
| ITGB1 | NM_002211.3 | TGGGTGGTGCACAAATTCAACATTTTTACAGGAAGGAATGCCTACTTCTGCACGATGTGATGATTTAGAAGCCTTAAAAAAGAAGGGTTGCCCTCCAGAT |
| ITGB2 | NM_000211.2 | CATCGACCTGTACTATCTGATGGACCTCTCCTACTCCATGCTTGATGACCTCAGGAATGTCAAGAAGCTAGGTGGCGACCTGCTCCGGGCCCTCAACGAG |
| ITGB5 | NM_002213.3 | CAAAGTTTCAGAGCGAGCGATCCAGGGCCCGCTATGAAATGGCTTCAAATCCATTATACAGAAAGCCTATCTCCACGCACACTGTGGACTTCACCTTCAA |
| ITK | NM_005546.3 | GCCAGTAAAGAAGTCAGTATAGAACCACTAGCGAATAGTGTTGCTCTGGCACAGACCACTGTGGTTGATGGCATGGCCCTCCAACTTGGAATAGGATTTT |
| JAK2 | NM_004972.3 | TTGATGTCAGTATTAAGCAAGCAAACCAAGAGGGTTCAAATGAAAGCCGAGTTGTAACTATCCATAAGCAAGATGGTAAAAATCTGGAAATTGAACTTAG |
| KANSL1 | NM_001193465.1 | GGTCATATTTCAGAGTCACTGTCTACCAAATCATGTGGAGCACTCAGACCTGTCAATGGAGTTATTAACACTCTTCAGCCTGTCTTGGCAGACCACATTC |
| KAT6A | NM_001099412.1 | AAACTCGCAAACCCGCTTTATACTGAGTGGATTTTGGAGGCCATCAAAAAAGTGAAAAAGCAGAAACAGCGTCCTTCAGAAGAAAGGATATGCAATGCTG |
| KDM3B | NM_016604.3 | AAGAGCAAGGCCAGCCTACCCAACTTTCTTGACCACATCATTGCCTCAGTGGTAGAAAATAAGAAAACCTCAGATGCTTCAAAGCGGGCCTGCAACTTGA |
| KEAP1 | NM_012289.3 | CTCATTGAATTCGCCTACACGGCCTCCATCTCCATGGGCGAGAAGTGTGTCCTCCACGTCATGAACGGTGCTGTCATGTACCAGATCGACAGCGTTGTCC |
| KIF2C | NM_006845.3 | CCTGCTCTAACGGGGCGCTGATTCCAGGCAATTTATCCAAGGAAGAGGAGGAACTGTCTTCCCAGATGTCCAGCTTTAACGAAGCCATGACTCAGATCAG |
| KIR3DL1/2 | NM_001322168.1 | AATCAGGAGAGAGAGTCATCCTGCAATGTTGGTCAGATATCATGTTTGAGCACTTCTTTCTGCACAAAGAGGGGATCTCTAAGGACCCCTCACGCCTCGT |
| KIR3DL3 | NM_153443.3 | GGGTTCCCAGGTCAACTATTCCATGGGTCCCATGACACCTGCCCTTGCAGGGACCTACAGATGCTTTGGTTCTGTCACTCACTTACCCTATGAGTTGTCG |
| KLRB1 | NM_002258.2 | TGAGTTAAACTTACCCACAGACTCAGGCCCAGAAAGTTCTTCACCTTCATCTCTTCCTCGGGATGTCTGTCAGGGTTCACCTTGGCATCAATTTGCCCTG |
| KLRD1 | NM_007334.2 | TGGTCTTGAACTCCTGGCCTCAAGGGATTCTCCCACCTTGGATTCCCAAAGTGCTGGGATTATAGGTGTGAACCACCATCCCTGGCCCTCTTCACATTCT |
| KLRK1 | NM_007360.1 | GGACCAGGATTTACTTAAACTGGTGAAGTCATATCATTGGATGGGACTAGTACACATTCCAACAAATGGATCTTGGCAGTGGGAAGATGGCTCCATTCTC |
| KMO | NM_003679.3 | GATGAAGAAACCTCGCTTTGATTACAGTCAGCAGTACATTCCTCATGGGTACATGGAGTTGACTATTCCACCTAAGAACGGAGATTATGCCATGGAACCT |
| KMT2A | NM_005933.2 | CTCTGCCTCTGCCACAAGGTTTCAGAGTAGTGTAGTCCAAGTAGAGGGTGGGGCACCCTTTTCTCGCCGCAAGAAGCCCATTCCTATGGAAGTCTAGCAA |
| KMT2D | NM_003482.3 | CCAACATTAATTTTCCTAATCTCAAGCAAGACTACCCAGACTGGTCAAGCCGTTGCAAACAAATCATGAAGCTCTGGAGAAAGGTTCCAGCAGCTGACAA |
| KMT2E | NM_018682.3 | TCTAAATTTCATGGGCTAGAAATGTGTGTTGATGCAAGGACTTTTGGGAATGAGGCTCGATTCATCAGGCGGTCTTGTACACCCAATGCAGAGGTGAGGC |
| KPNA2 | NM_002266.2 | TGATGATCCAGAAGTATTAGCAGATACCTGCTGGGCTATTTCCTACCTTACTGATGGTCCAAATGAACGAATTGGCATGGTGGTGAAAACAGGAGTTGTG |
| KRAS | NM_033360.2 | GGACGAATATGATCCAACAATAGAGGATTCCTACAGGAAGCAAGTAGTAATTGATGGAGAAACCTGTCTCTTGGATATTCTCGACACAGCAGGTCAAGAG |
| KRT1 | NM_006121.2 | TGCAGCAGGTAGATACCTCCACTAGAACCCATAATTTAGAGCCCTACTTTGAGTCATTCATCAACAATCTCCGAAGGAGAGTGGACCAACTGAAGAGTGA |
| KYAT1 | NM_004059.4 | TGCTATTGGGAAACCTCTTCTCCGTGACACAGAATGTTCTGGGTGGGAGCCGCCCTTCTTCATCTTAGAGAACCAAGTGCCTCCTGTCTGAAAGGTGAGG |
| KYAT3 | NM_001008661.2 | CTTGAAAGTGTTGGCCTAAAACCCATAGTTCCTGATGGAGGATACTTCATCATCGCTGATGTGTCTTTGCTAGATCCAGACCTCTCTGATATGAAGAATA |
| KYNU | NM_003937.2 | TATCCTTGAAGTAATTGAGAAGGAAGGAGACTCAATTGCAGTGATCCTGTTCAGTGGGGTGCATTTTTACACTGGACAGCACTTTAATATTCCTGCCATC |
| L2HGDH | NM_024884.2 | CCAGGCCCTGGATAGAGATGGAAATCTGGTAGAAGATTTTGTATTTGATGCAGGAGTTGGGGATATTGGAAATCGCATTCTTCATGTGAGAAATGCACCT |
| LAG3 | NM_002286.5 | TTTTGGTGACTGGAGCCTTTGGCTTTCACCTTTGGAGAAGACAGTGGCGACCAAGACGATTTTCTGCCTTAGAGCAAGGGATTCACCCTCCGCAGGCTCA |
| LAMA4 | NM_001105209.1 | CCGAGCCCTGGAAGAGCACTACTGGATGTCAGCGGAGAAATGGCTTTGAGCTCAGCCTGGCGCTCGGTTCTGCCTCTGTGGCTCCTCTGGAGCGCTGCCT |
| LAMB1 | NM_002291.2 | TTGCCAGGAGCTGCTACCAAGATCCTGTTACTTTACAGCTTGCCTGTGTTTGTGATCCTGGATACATTGGTTCCAGATGTGACGACTGTGCCTCAGGATA |
| LAMC1 | NM_002293.3 | TCTTGATAGGAAAGTGTCTGACCTGGAGAATGAAGCCAAGAAGCAGGAGGCTGCCATCATGGACTATAACCGAGATATCGAGGAGATCATGAAGGACATT |
| LAMTOR2 | NM_001145264.1 | TACGGGGACACTGACGCCCGGGTCACCGCTGCCATAGCCAGTAACATCTGGGCCGCCTACGACCGGAACGGGAACCAAGCGTTTAATGAAGACAATCTCA |
| LAMTOR4 | NM_001008395.2 | CTGGACGGGCCCAGGCTTGGGGACTCTGAGCTGTGTTAAGGAGAACAAGGGCAAGGAGACCTCCCTTTGTGCTCCCTCACTCCCTAATAAACATGAGTCT |
| LAMTOR5 | NM_006402.2 | AGAATCAGATAATGGGAACATTATGATCCAGAAACACGATGGCATCACGGTGGCAGTGCACAAAATGGCCTCTTGATGCTCATATCTGTTCTTCAGCAGC |
| LAT | NM_001014987.1 | TGTGTAATAGAATAAAGGCCTGCGTGTGTCTGTGTTGAGCGTGCGTCTGTGTGTGCCTGTGTGCGAGTCTGAGTCAGAGATTTGGAGATGTCTCTGTGTG |
| LCK | NM_005356.2 | ATTAAGTGGACAGCGCCAGAAGCCATTAACTACGGGACATTCACCATCAAGTCAGATGTGTGGTCTTTTGGGATCCTGCTGACGGAAATTGTCACCCACG |
| LDHA | NM_001165414.1 | AACTTCCTGGCTCCTTCACTGAACATGCCTAGTCCAACATTTTTTCCCAGTGAGTCACATCCTGGGATCCAGTGTATAAATCCAATATCATGTCTTGTGC |
| LDHB | NM_001174097.1 | CTGTGACCGCCAATTCTAAGATTGTAGTGGTAACTGCAGGAGTCCGTCAGCAAGAAGGGGAGAGTCGGCTCAATCTGGTGCAGAGAAATGTTAATGTCTT |
| LDHC | NM_002301.2 | TTGGATTATTGGAGAACATGGTGATTCTAGTGTGCCCTTATGGAGTGGGGTGAATGTTGCTGGTGTTGCTCTGAAGACTCTGGACCCTAAATTAGGAACG |
| LEPR | NM_001003679.1 | TTCCAGACTTGTGTGCAGTCTATGCTGTTCAGGTGCGCTGTAAGAGGCTAGATGGACTGGGATATTGGAGTAATTGGAGCAATCCAGCCTACACAGTTGT |
| LTA | NM_000595.2 | CTGATCAAGTCACCGGAGCTTTCAAAGAAGGAATTCTAGGCATCCCAGGGGACCACACCTCCCTGAACCATCCCTGATGTCTGTCTGGCTGAGGATTTCA |
| LTA4H | NM_001256643.1 | ATTTGGGGAGACACATCCTTTCACCAAACTTGTGGTTGATCTGACAGATATAGACCCTGATGTAGCTTATTCTTCAGTTCCCTATGAGAAGGGCTTTGCT |
| LTB | NM_002341.1 | AGGAACAGGCGTTTCTGACGAGCGGGACGCAGTTCTCGGACGCCGAGGGGCTGGCGCTCCCGCAGGACGGCCTCTATTACCTCTACTGTCTCGTCGGCTA |
| LTC4S | NM_145867.1 | AGACGGGGCTAAGCGTTCCCCAGCTCGCCTTCACACACAGCCCGTGCCACCACACCGACGGTACCATGAAGGACGAGGTAGCTCTACTGGCTGCTGTCAC |
| LY86 | NM_004271.3 | GAGAGAGGACATCAAAGAGCTTTTTCTTGACCTAGCTCTCATGTCTCAAGGCTCATCTGTTTTGAATTTCTCCTATCCCATCTGTGAGGCGGCTCTGCCC |
| LY96 | NM_015364.2 | AGAAGTTATTTGCCGAGGATCTGATGACGATTACTCTTTTTGCAGAGCTCTGAAGGGAGAGACTGTGAATACAACAATATCATTCTCCTTCAAGGGAATA |
| MAP1LC3B | NM_022818.4 | AGAGCCGTACGCTCTTTACAGATACTAATGTCAAGAGTTAAACCTCCTCAGGTTCAACCTGTGATAAAAGACTAGTGCTTCCCAGTACTTGCATGGGGTT |
| MAP2K1 | NM_002755.2 | ACGGAATGGACAGCCGACCTCCCATGGCAATTTTTGAGTTGTTGGATTACATAGTCAACGAGCCTCCTCCAAAACTGCCCAGTGGAGTGTTCAGTCTGGA |
| MAP2K2 | NM_030662.3 | TGGAGGAGCTGGAACTTGACGAGCAGCAGAAGAAGCGGCTGGAAGCCTTTCTCACCCAGAAAGCCAAGGTCGGCGAACTCAAAGACGATGACTTCGAAAG |
| MAP2K3 | NM_145109.1 | GAACCTGGACTCCCGGACCTTCATCACCATTGGAGACAGAAACTTTGAGGTGGAGGCTGATGACTTGGTGACCATCTCAGAACTGGGCCGTGGAGCCTAT |
| MAP3K12 | NM_006301.2 | AGGGATCTCAAGTCACCCAACATGCTAATCACCTACGACGATGTGGTGAAGATCTCAGATTTTGGCACTTCCAAGGAGCTGAGTGACAAGAGCACCAAGA |
| MAPK1 | NM_138957.2 | ACTGCCAGAGAACCCTGAGGGAGATAAAAATCTTACTGCGCTTCAGACATGAGAACATCATTGGAATCAATGACATTATTCGAGCACCAACCATCGAGCA |
| MAPK8 | NM_002750.2 | TCTCTGTAGATGAAGCTCTCCAACACCCGTACATCAATGTCTGGTATGATCCTTCTGAAGCAGAAGCTCCACCACCAAAGATCCCTGACAAGCAGTTAGA |
| MAPK8IP1 | NM_005456.2 | TCCCAATTTCAGGCTCACCCATGACATCAGCCTGGAGGAGTTTGAGGATGAAGACCTCTCGGAGATCACTGATGAGTGTGGCATCAGCTTACAGTGCAAA |
| MAPKAP1 | NM_001006617.1 | GCTCATGGATTCTCCCTTATTCAGGTGGACAACACAAAGGTTACCATGAAGGAAATCTTACTGAAGGCAGTGAAGCGAAGAAAAGGATCCCAGAAAGTTT |
| MAPT | NM_016834.3 | ATTGGGTCCCTGGACAATATCACCCACGTCCCTGGCGGAGGAAATAAAAAGATTGAAACCCACAAGCTGACCTTCCGCGAGAACGCCAAAGCCAAGACAG |
| MAT1A | NM_000429.2 | GGGGATTAACCCACTAGCTCTTGTTGAGCCGTGGGCAATTGTCTGAAAAGTGAAGACAGAACCACAGGGCTATTTTGTTTGCTTCATGTGTCCCAGAAGA |
| MAT2A | NM_005911.4 | AAGCAGTTGTGCCTGCGAAATACCTTGATGAGGATACAATCTACCACCTACAGCCAAGTGGCAGATTTGTTATTGGTGGGCCTCAGGGTGATGCTGGTTT |
| MCAT | NM_014507.3 | GCAGGAGACCCTGGACCGCACCGTGCACTGTCAGCCCGCGATCTTCGTGGCATCGCTGGCCGCTGTCGAGAAACTACATCACCTGCAGCCCTCGGTGATT |
| ME2 | NM_002396.3 | GCCTGCTCCCAGTATGGACACATCTTTAGAAGACCTAAGGGATTATTTATTTCGATCTCAGACAGAGGTCATGTTAGATCAATTGTGGATAACTGGCCAG |
| MGST3 | NM_004528.2 | CAAAGTGGAGTATCCTATCATGTACAGCACGGACCCTGAAAATGGGCACATCTTCAACTGCATTCAGCGAGCCCACCAGAACACGTTGGAAGTGTATCCT |
| MKI67 | NM_002417.2 | AGCAGATGTAGAGGGAGAACTCTTAGCGTGCAGGAATCTAATGCCATCAGCAGGCAAAGCCATGCACACGCCTAAACCATCAGTAGGTGAAGAGAAAGAC |
| MLST8 | NM_001199173.1 | GGCTATCCACATCTGGGACTTGAAAACAGACCACAACGAGCAGCTGATCCCTGAGCCCGAGGTCTCCATCACGTCCGCCCACATCGATCCCGACGCCAGC |
| MLYCD | NM_012213.2 | CCGGCTAAGAAAACGATCATTTTCAGGAGGGGCCGGGAGTTATGTATCTGAAGCAGCTTTCCAAGCAAAGCCAAAGTTGACTGTGTTCTTGTCCCGCAGC |
| MPC1 | NM_016098.2 | CAGTAGCCAACTGGGGTCTTCCCATTGCTGCCATCAATGATATGAAAAAGTCTCCAGAGATTATCAGTGGGCGGATGACATTTGCCCTCTGTTGCTATTC |
| MPC2 | NM_001143674.1 | TTCTGGGCTCCAATTATGAAATGGGGGTTGGTGTGTGCTGGATTGGCTGATATGGCCAGACCTGCAGAAAAACTTAGCACAGCTCAATCTGCTGTTTTGA |
| MPO | NM_000250.1 | AGACCTGCTGGAGAGGAAGCTGCGGTCCCTGTGGCGAAGGCCATTCAATGTCACTGATGTGCTGACGCCCGCCCAGCTGAATGTGTTGTCCAAGTCAAGC |
| MRAS | NM_001085049.2 | AGATCTGTCTCTTGCTGCGTTTTCACATCAGCTGTGCTGCTTGGTGCCTCTCTGATACGAATACACTGACACGTCAAAGTAACCTAATGTGGACACCATC |
| MS4A1 | NM_152866.2 | CTTCTGATGATCCCAGCAGGGATCTATGCACCCATCTGTGTGACTGTGTGGTACCCTCTCTGGGGAGGCATTATGTATATTATTTCCGGATCACTCCTGG |
| MS4A2 | NM_000139.3 | GCAAATCTTGCTCTCCCACAGGAGCCTTCCAGTGTGCCTGCATTTGAAGTCTTGGAAATATCTCCCCAGGAAGTATCTTCAGGCAGACTATTGAAGTCGG |
| MS4A4A | NM_024021.2 | GTTTGAGGCCACCAAAAGATCAACAGACAAATGCTCCAGAAATCTATGCTGACTGTGACACAAGAGCCTCACATGAGAAATTACCAGTATCCAACTTCGA |
| MSH2 | NM_000251.1 | CGACAAACTGGGGTGATAGTACTCATGGCCCAAATTGGGTGTTTTGTGCCATGTGAGTCAGCAGAAGTGTCCATTGTGGACTGCATCTTAGCCCGAGTAG |
| MSRB2 | NM_012228.3 | GCTGCGACAGTCCACTCTTCAGTTCTGAGAAAAAGTACTGCTCTGGCACTGGGTGGCCTTCGTTTTCCGAGGCTCATGGTACGTCTGGCTCTGATGAAAG |
| MTF1 | NM_005955.2 | GATAAAATGCTCAGGTTTGTGGATAAAAACGGACTGGTGCCTTCCTCATCTGGAACTGTTTATGATAGGACCACTGTTCTTATTGAGCAGGACCCTGGCA |
| MTOR | NM_004958.3 | TCACTCTTGCCCTCCGAACGCTTGGCAGCTTTGAATTTGAAGGCCACTCTCTGACCCAATTTGTTCGCCACTGTGCGGATCATTTCCTGAACAGTGAGCA |
| MYB | NM_001130173.1 | CCGCGCCCGCCGCGCCATGGCCCGAAGACCCCGGCACAGCATATATAGCAGTGACGAGGATGATGAGGACTTTGAGATGTGTGACCATGACTATGATGGG |
| MYBL1 | NM_001080416.3 | TTCAACACAAACCTTGTGCAGCTATGGATCATATGCAAACCCAGAATCAGTTTTACATACCTGTTCAGATCCCTGGGTATCAGTATGTGTCACCTGAAGG |
| MYBL2 | NM_002466.2 | GAATCCAGACCTTGTCAAGGGGCCATGGACCAAAGAGGAAGACCAAAAAGTCATCGAGCTGGTTAAGAAGTATGGCACAAAGCAGTGGACACTGATTGCC |
| MYC | NM_002467.3 | TCGGACACCGAGGAGAATGTCAAGAGGCGAACACACAACGTCTTGGAGCGCCAGAGGAGGAACGAGCTAAAACGGAGCTTTTTTGCCCTGCGTGACCAGA |
| MYCL | NM_001033081.2 | GCAGGGAGCGGACATGGACTACGACTCGTACCAGCACTATTTCTACGACTATGACTGCGGGGAGGATTTCTACCGCTCCACGGCGCCCAGCGAGGACATC |
| MYCN | NM_005378.4 | AAGGCCGCCAAGGTGGTCATTTTGAAAAAGGCCACTGAGTATGTCCACTCCCTCCAGGCCGAGGAGCACCAGCTTTTGCTGGAAAAGGAAAAATTGCAGG |
| MYD88 | NM_002468.3 | ACGTTTTTCTAGGTACAGCTCCCAGGAACAGCTAGGTGGGAAAGTCCCATCACTGAGGGAGCCTAACCATGTCCCTGAACAAAAATTGGGCACTCATCTA |
| NAALAD2 | NM_005467.3 | CTGCAGCCCAGAGAGGAAATGTGTTAAATTTGAATGGTGCTGGTGACCCACTCACTCCAGGCTATCCAGCAAAAGAATACACTTTCAGACTTGATGTTGA |
| NADK | NM_001198993.1 | ATCTGCGTGTGTCTGTGACCGCCTGTCTCAGTGGCACGGCCACTTCCTTTCTGTAGCTGGGTTAGAGCCTGGGTCTGCCTTTTGTCCAGATCAGCTGTTT |
| NADK2 | NM_153013.3 | AGAACGGTCTGAGGGTCATTTATGCCTGCCCGTTCGATATACACATTCCTTTCCAGAAGCCTTACAGAAGTTCTATCGTGGTGAGTTCAGGTGGTTGTGG |
| NAGLU | NM_000263.3 | GGAGCTGACCGAGGCCACGCCCAACAGGTACCGCTATTACCAGAATGTGTGCACGCAAAGCTACTCTTTCGTGTGGTGGGACTGGGCCCGCTGGGAGCGA |
| NAT8L | NM_178557.3 | GCCTTCTCTCATTCTGGTGGCATCTGCGCCCGTGAGTGACCTCTTCCTTGGCTGCACTGCCCTGTGGGTGGTGAGACGCTTGGCCTTTTTTGTTGCTGCC |
| NCAPH | NM_015341.3 | TGCTACTATTCTGACCAAGTCCACTTTGGAGAACCAGAATTGGAGAGCTACCACCCTTCCTACAGATTTCAACTACAATGTTGACACTCTGGTCCAGCTT |
| NCOA2 | NM_006540.2 | GCTGGGAGGACCTGGTAAGAAGGTGTATTCAGAAGTTCCATGCGCAGCATGAAGGAGAATCTGTGTCCTATGCTAAGAGGCATCATCATGAAGTACTGAG |
| NCOR1 | NM_006311.3 | TAGGAGTGAGCATGAGATTTCTGAAATTATTGATGGGCTCTCTGAGCAGGAGAATAATGAGAAACAAATGCGGCAGCTCTCTGTGATTCCACCTATGATG |
| NCR1 | NM_004829.5 | CGATGTTTTGGCTCCTATAACAACCATGCCTGGTCTTTCCCCAGTGAGCCAGTGAAGCTCCTGGTCACAGGCGACATTGAGAACACCAGCCTTGCACCTG |
| NDC1 | NM_001168551.1 | CAAATGCATATTTGGGCATTAGAAGGTCTGTCGCACTTAGTAGCAGCATCATTTACAGAGGATAGATTTGGAGTTGTCCAGACGACACTACCAGCTATCC |
| NDUFA1 | NM_004541.3 | AAGGAAAAAAGGGTTGCTCATTTTGGGTATCACTGGAGTCTGATGGAAAGAGATAGGCGCATCTCTGGAGTTGATCGTTACTATGTGTCAAAGGGTTTGG |
| NDUFA11 | NM_175614.4 | CCGGTCCCCGGCGCGTGCGCGCGATCCATGTCCATGTCCGCGCCTATCAATAAAGTTGCTCACTTGTTGCCGGCCCGCTAGCCCGAAAGGTTGCGCGCGC |
| NDUFA12 | NM_018838.3 | TCGTTGGCTTCACAGTATGACTGATGATCCTCCAACAACAAAACCACTTACTGCTCGTAAATTCATTTGGACGAACCATAAATTCAACGTGACTGGCACC |
| NDUFA13 | NM_015965.6 | CACTGGAGCATAATGAAGTGGAACCGTGAGCGCAGGCGCCTACAAATCGAGGACTTCGAGGCTCGCATCGCGCTGTTGCCACTGTTACAGGCAGAAACCG |
| NDUFA2 | NM_001185012.1 | ATGGGCTAGGCTTTAGGGTCCGCGGTTGGTCAGACCGGAGCACTTGGCCTGAAGACCTGGAATTGGCGACTTCGATATTAACAAGGATGGCGGCGGCCGC |
| NDUFA3 | NM_004542.3 | CCAGTGCTGGTCGTGTCCTTCGTCGTCGGGGGCCTCGCTGTAATTCTGCCCCCATTGAGCCCCTACTTCAAGTACTCCGTCATGATCAACAAGGCCACGC |
| NDUFA4 | NM_002489.2 | AAGTCCGTAGTGTCTCATTGCAGATAATTTTTAGCTTAGGGCCTGGTGGCTAGGTCGGTTCTCTCCTTTCCAGTCGGAGACCTCTGCCGCAAACATGCTC |
| NDUFA6 | NM_002490.3 | GAAGCAGCGGACACATGTTATGCGGTTCTTCCATGAAACAGAAGCGCCAAGGCCAAAGGATTTCCTATCCAAGTTCTATGTTGGCCACGATCCATGAAGT |
| NDUFA7 | NM_005001.2 | CCTGTGACACTGCACCCTCACGGCCACCCGACTACTTTGCCTCCTTGGATTTCCTCCAGGGAGAATGTGACCTAATTTATGACAAATACGTAGAGCTCAG |
| NDUFB1 | NM_004545.3 | AACGGCTAACTGCCTTCCGGAACAAGAGTATGTTATTTAAAAGGGAATTGCAACCCAGTGAAGAAGTTACCTGGAAGTAAAGACTGGCTAGATTATCGAA |
| NDUFB10 | NM_004548.2 | GCACGCCGGTGCAGCCCAATCCCATCGTCTACATGATGAAAGCGTTCGACCTCATCGTGGACCGACCCGTGACCCTCGTGAGAGAATTTATAGAGCGGCA |
| NDUFB11 | NM_001135998.1 | CCGTTTTGGACGTCTGGAACATGCGACTTGTCTTCTTCTTTGGCGTCTCCATCATCCTGGTCCTTGGCAGCACCTTTGTGGCCTATCTGCCTGACTACAG |
| NDUFB2 | NM_004546.2 | TCCAGAGCGAGTTCTTCAGCGGACTCATGTGGTTCTGGATTCTCTGGCGCTTTTGGCATGACTCAGAAGAGGTGCTGGGTCACTTTCCGTATCCTGATCC |
| NDUFB4 | NM_004547.4 | TCATCGAAAATCCTGCCTTGCTTCGTTGGGCCTATGCAAGAACAATAAATGTCTATCCTAATTTCAGACCCACTCCTAAAAACTCACTCATGGGAGCTCT |
| NDUFB7 | NM_004146.5 | CTGCGGGACTACTGCGCCCACCACCTCATCCGGCTGCTCAAGTGCAAGCGTGACAGCTTCCCCAACTTCCTGGCCTGCAAGCAGGAGCGGCACGACTGGG |
| NDUFB8 | NM_001284367.1 | CCAGAAGAACGGGCCGCCGCCGCCAAGAAGTATAATATGCGTGTGGAAGACTACGAACCTTACCCGGATGATGGCATGGGGTATGGCGACTACCCGAAGC |
| NDUFS7 | NM_024407.4 | GCCAGCCCGCGCCAGTCCGACGTCATGATCGTGGCCGGCACACTCACCAACAAGATGGCCCCAGCGCTTCGCAAGGTCTACGACCAGATGCCGGAGCCGC |
| NDUFS8 | NM_002496.3 | GGAGCCTCCACAGCAGTGCAGTGGCAGCCACCTACAAGTATGTGAACATGCAGGATCCCGAGATGGACATGAAGTCAGTGACTGACCGGGCAGCCCGCAC |
| NEDD8 | NM_006156.2 | AGGTGGTCTTAGGCAGTGATGGACCCTCCATTTTACCTCTTTACCCTGTCGCTCATAATGAGGCATCATATATCCTCTCACTCTCTGGGACACCATAGCC |
| NEU1 | NM_000434.3 | CTTTAGACTCCAGCCTTGGCAAAATCACCTTCCCTTTACCAGGGAAATCACTTCCTTTAGGACTGAAAGCTAGGCGTCCTCTCCCACAAAAAAGTCCTGC |
| NFAT5 | NM_173214.1 | CCCTGACAACTATTCAAACCCAGGACATCTCACAGCCTGGTACTTTTCCAGCAGTTTCTGCTTCTAGTCAGCTGCCCAACAGCGATGCACTATTGCAGCA |
| NFE2L2 | NM_006164.3 | TCCCGGTCACATCGAGAGCCCAGTCTTCATTGCTACTAATCAGGCTCAGTCACCTGAAACTTCTGTTGCTCAGGTAGCCCCTGTTGATTTAGACGGTATG |
| NFKB1 | NM_003998.2 | AGGGTATAGCTTCCCACACTATGGATTTCCTACTTATGGTGGGATTACTTTCCATCCTGGAACTACTAAATCTAATGCTGGGATGAAGCATGGAACCATG |
| NFKB2 | NM_002502.2 | ATCTCCGGGGGCATCAAACCTGAAGATTTCTCGAATGGACAAGACAGCAGGCTCTGTGCGGGGTGGAGATGAAGTTTATCTGCTTTGTGACAAGGTGCAG |
| NFS1 | NM_021100.3 | GACCATGATGCTCTACATGGACATTTGAGTCTTCGTCTTCTGCTGCTGCTCGGCTGGACCAGCTTCTTTAACAGCAAGCATAATCCACTTCAATGTAATA |
| NGFR | NM_002507.3 | TGAAGAAAAGTGGGCCAGTGTGGGAATGCGGCAAGAAGGAATTGACTTCGACTGTGACCTGTGGGGATTTCTCCCAGCTCTAGACAACCCTGCAAAGGAC |
| NKG7 | NM_005601.3 | TGTGGCGGTCCCCGTCCTGGCTATGAAACCTTGTGAGCAGAAGGCAAGAGCGGCAAGATGAGTTTTGAGCGTTGTATTCCAAAGGCCTCATCTGGAGCCT |
| NME1 | NM_000269.2 | CTTGTGGTTTCACCCTGAGGAACTGGTAGATTACACGAGCTGTGCTCAGAACTGGATCTATGAATGACAGGAGGGCAGACCACATTGCTTTTCACATCCA |
| NME2 | NM_001018137.2 | TTGACTACAAGTCTTGTGCTCATGACTGGGTCTATGAATAAGAGGTGGACACAACAGCAGTCTCCTTCAGCACGGCGTGGTGTGTCCCTGGACACAGCTC |
| NOD2 | NM_001293557.1 | TGCCTTCCTTCTACAACATGTTCAGGAATTACCAGTCCCATTGGCCCTGCCTTTGGAAGCTGCCACATGCAAGAAGTATATGGCCAAGCTGAGGACCACG |
| NOS1 | NM_000620.4 | CAGCCAATGTGCAGTTCACAGAGATATGCATACAGCAGGGCTGGAAACCGCCTAGAGGCCGCTTCGATGTCCTGCCGCTCCTGCTTCAGGCCAACGGCAA |
| NOS2 | NM_000625.4 | TTGCCTGGGGTCCATTATGACTCCCAAAAGTTTGACCAGAGGACCCAGGGACAAGCCTACCCCTCCAGATGAGCTTCTACCTCAAGCTATCGAATTTGTC |
| NOS3 | NM_000603.4 | CCGGACCACCTCGTCCCTGTGGAAAGACAAGGCAGCAGTGGAAATCAACGTGGCCGTGCTGCACAGTTACCAGCTAGCCAAAGTCACCATCGTGGACCAC |
| NOX1 | NM_007052.4 | TCATTTTGCAGCCGCACACTGAGAAAGCAATTGGATCACAACCTCACCTTCCACAAGCTGGTGGCCTATATGATCTGCCTACATACAGCTATTCACATCA |
| NOX3 | NM_015718.2 | TCAGATTTCACAAACTGGTCGCCTATGGGATAGCTGTTAATGCAACCATCCACATCGTGGCGCATTTCTTCAACCTGGAACGCTACCACTGGAGCCAGTC |
| NOX4 | NM_001143836.2 | ATAGCAAAATATAACAGAGGAAAAACAGTTGGTGTTTTCTGTTGTGGACCCAATTCACTATCCAAGACTCTTCATAAACTGAGTAACCAGAACAACTCAT |
| NPM1 | NM_002520.6 | AAAAGGACAAGAATCCTTCAAGAAACAGGAAAAAACTCCTAAAACACCAAAAGGACCTAGTTCTGTAGAAGACATTAAAGCAAAAATGCAAGCAAGTATA |
| NPR1 | NM_000906.3 | ACCATGCCGCGCAAAGGCCGAGTTATCTACATCTGCAGCTCCCCTGATGCCTTCAGAACCCTCATGCTCCTGGCCCTGGAAGCTGGCTTGTGTGGGGAGG |
| NPR2 | NM_003995.3 | TCGAATTGTGGAAAAGATGCAGGGACGAAGATATCACGGTGTAACTGGGCTGGTTGTCATGGACAAGAACAATGACCGAGAGACTGACTTTGTCCTCTGG |
| NQO1 | NM_000903.2 | CCGAATTCAAATCCTGGAAGGATGGAAGAAACGCCTGGAGAATATTTGGGATGAGACACCACTGTATTTTGCTCCAAGCAGCCTCTTTGACCTAAACTTC |
| NR2F1 | NM_005654.4 | AAACTCTCATCCGCGATATGTTACTGTCTGGGAGCAGCTTCAACTGGCCTTACATGTCCATCCAGTGCTCCTAGACCTTGGGCGCTTCCCACCTGCCCCG |
| NRAS | NM_002524.3 | ACCCTGGTCCTGACTTCCCTGGAGGAGAAGTATTCCTGTTGCTGTCTTCAGTCTCACAGAGAAGCTCCTGCTACTTCCCCAGCTCTCAGTAGTTTAGTAC |
| NRF1 | NM_001040110.1 | CAGCTTGCCTCCTCAGAGGTGTTGACTATCTGGGTGTTCTTGGTAACCGTTAACTCTGTCTTTCTCAGCTGCAAGCCCTGAGTCTCCAGTAGCTGAATTC |
| NSD1 | NM_022455.4 | CAAGAGAGACCTCCCTGCTTCTGGTAAAAGTCGTTCAGACTGTGTTACTAGGCGCAACTGTGGACGATCAAAGCCTTCATCCAAATTGCGAGATGCTTTT |
| NT5E | NM_002526.2 | ATTCGGGTTTTGAAATGGATAAACTCATCGCTCAGAAAGTGAGGGGTGTGGACGTCGTGGTGGGAGGACACTCCAACACATTTCTTTACACAGGCAATCC |
| NUP205 | NM_015135.1 | GAACTTGACGTTGATGTAAATGAAGGGTCTCTAATGGAGCTACAGGGACATATTGGAAGATTCCAGCGCCAGTGCTTAGGACTACTAAGTCGCTTTGGTG |
| NUP62 | NM_016553.3 | GGCACTGCAAAGACGGCAACAACCACACCTGCTACAGGGTTTTCTTTCTCCACCTCTGGCACTGGAGGGTTTAATTTTGGGGCTCCCTTCCAACCAGCCA |
| OAT | NM_000274.3 | CTGGAGCGTGCTCTTCAGGATCCAAATGTGGCTGCGTTCATGGTAGAACCAATTCAGGGTGAAGCAGGCGTTGTTGTTCCGGATCCAGGTTACCTAATGG |
| ODC1 | NM_002539.1 | AGACCTTCGTGCAGGCAATCTCTGATGCCCGCTGTGTTTTTGACATGGGGGCTGAGGTTGGTTTCAGCATGTATCTGCTTGATATTGGCGGTGGCTTTCC |
| OGDH | NM_001003941.2 | CATTGACGGCTTCCCAGACTGTTAAGACATTTTCACAAAACAGACCAGCAGCAGCTAGGACATTTCAACAGATTCGGTGCTATTCTGCACCTGTTGCTGC |
| OGDHL | NM_018245.2 | GCGAACCTGCTGGGTGATTTGTTTGCGCTCTGTTTTATGGGGCATTCCTGCGAGATGTGTCAGCTTCTGTGTGAAATGCAGCCACAGCTCATGTGTACCA |
| OTC | NM_000531.5 | CTTACCACACAAGATATTCATTTGGGTGTGAATGAAAGTCTCACGGACACGGCCCGTGTATTGTCTAGCATGGCAGATGCAGTATTGGCTCGAGTGTATA |
| PAH | NM_000277.1 | TTCAAGAGCTGGACAGATTTGCCAATCAGATTCTCAGCTATGGAGCGGAACTGGATGCTGACCACCCTGGTTTTAAAGATCCTGTGTACCGTGCAAGACG |
| PCK1 | NM_002591.2 | GGGCACATCAACATGATGGAGCTTTTCAGCATCTCCAAGGAATTCTGGGAGAAGGAGGTGGAAGACATCGAGAAGTATCTGGAGGATCAAGTCAATGCCG |
| PCK2 | NM_004563.2 | GGGGAGCCAGTGAGCCAGTGGCCGTGCAACCCAGAGAAAACCCTGATTGGCCACGTGCCCGACCAGCGGGAGATCATCTCCTTCGGCAGCGGCTATGGTG |
| PCLAF | NM_014736.5 | AGGGTTTAAACGGTTGATAATGCCTCTACAACAACAAGAAAAAAGATAAAATACTAGGATAGAATCATGGTGGGCACAGTGGCTTCTCAGGAGGCTGAGG |
| PDCD1 | NM_005018.2 | CTTCCCCGAGGACCGCAGCCAGCCCGGCCAGGACTGCCGCTTCCGTGTCACACAACTGCCCAACGGGCGTGACTTCCACATGAGCGTGGTCAGGGCCCGG |
| PDCD1LG2 | NM_025239.3 | TGTGGAGCTGTGGCAAGTCCTCATATCAAATACAGAACATGATCTTCCTCCTGCTAATGTTGAGCCTGGAATTGCAGCTTCACCAGATAGCAGCTTTATT |
| PDGFB | NM_033016.2 | GATTCACCTCTTCCTCTGGTTCCTTTCATCTCTCTACCTCCACCCTGCATTTTCCTCTTGTCCTGGCCCTTCAGTCTGCTCCACCAAGGGGCTCTTGAAC |
| PDGFRB | NM_002609.3 | CCCCTTCCTCCATCCCTCTGTTCTCCTGAGCCTTCAGGAGCCTGCACCAGTCCTGCCTGTCCTTCTACTCAGCTGTTACCCACTCTGGGACCAGCAGTCT |
| PDHA1 | NM_000284.3 | TAAGAGTGACCCTATTATGCTTCTCAAGGACAGGATGGTGAACAGCAATCTTGCCAGTGTGGAAGAACTAAAGGAAATTGATGTGGAAGTGAGGAAGGAG |
| PDK1 | NM_002610.3 | TGGATTGCCCATATCACGTCTTTACGCACAATACTTCCAAGGAGACCTGAAGCTGTATTCCCTAGAGGGTTACGGGACAGATGCAGTTATCTACATTAAG |
| PDK2 | NM_002611.3 | AGTTCACTGACGCCCTGGTCACCATCCGGAACCGGCACAACGACGTGGTGCCCACCATGGCACAAGGCGTGCTTGAGTACAAGGACACCTACGGCGATGA |
| PDK3 | NM_005391.1 | TCCCACCTGTAACGTGGCGGATGTGGTGAAAGATGCATATGAAACAGCCAAGATGCTGTGTGAACAGTATTACCTGGTAGCTCCAGAGCTGGAAGTTGAA |
| PDK4 | NM_002612.3 | AATCAGAACACTGATCCAATGAGGAATGGAGCTTGTTTCTGTGACCCAGGAGAACTTAGTGCAAGACTACAGGAGTTAACAGATGGCCAGCTCCTTATTT |
| PDP1 | NM_001161778.1 | GGCTTGATAATGACATCTCCTTGGAGGCGCAAGTTGGTGATCCTAATTCTTTTCTCAACTACCTGGTGCTTCGAGTGGCATTTTCTGGAGCCACTGCTTG |
| PDPK1 | NM_002613.3 | TCATTTCTCCACTGTAGTTGGGGTCCATTGATTGTGCAGGGGAACGTGCAGGAGGTTTTTCTAGGCACCGTGTTCAGTGCTGCTTCACTCTACCAGAGAT |
| PEBP1 | NM_002567.2 | CAGCGCTCGCTGACAGCTTGGGAGGAAACCTGAGATCTGTGTTTTTTAAATTGATCGTTCTTCATGGGGGTAAGAAAAGCTGGTCTGGAGTTGCTGAATG |
| PEMT | NM_148173.1 | AGCTTCTTTGCACTGGGGTTCGCTGGAACTTTCCTAGGTGATTACTTCGGGATCCTCAAGGAGGCGAGAGTGACCGTGTTCCCCTTCAACATCCTGGACA |
| PFKFB1 | NM_002625.2 | GCATAATTGCAGAAAACATCAGGCAAGTGAAACTTGGCAGCCCTGATTATATAGACTGTGACCGGGAAAAGGTTCTGGAAGACTTTCTAAAGAGAATTGA |
| PFKL | NM_001002021.1 | TGCGGAACGAGAAGTGCCATGACTACTACACCACGGAGTTCCTGTACAACCTGTACTCATCAGAGGGCAAGGGCGTCTTCGACTGCAGGACCAATGTCCT |
| PFKM | NM_000289.5 | TTTGATAGGAATTTTGCCACTAAGATGGGCGCCAAGGCTATGAACTGGATGTCTGGGAAAATCAAAGAGAGTTACCGTAATGGGCGGATCTTTGCCAATA |
| PGAM2 | NM_000290.3 | ATGGCCACTCACCGCCTCGTGATGGTCCGGCACGGCGAGAGCACATGGAACCAGGAGAACCGTTTCTGTGGCTGGTTCGATGCAGAGCTGAGTGAAAAGG |
| PGD | NM_002631.2 | AGGGCAGTTTATCCACACCAACTGGACAGGCCATGGTGGCACCGTGTCATCCTCGTCATACAATGCCTGATCATGCTGCTCCTGTCACCCTCCACGATTC |
| PGK1 | NM_000291.2 | GCAAGAAGTATGCTGAGGCTGTCACTCGGGCTAAGCAGATTGTGTGGAATGGTCCTGTGGGGGTATTTGAATGGGAAGCTTTTGCCCGGGGAACCAAAGC |
| PGM2 | NM_018290.3 | GACTTGACCATCATCCAGACTACACAGGGATTTTGCAGATACCTGGAAAAACAATTCAGTGACTTAAAGCAGAAAGGCATCGTGATCAGTTTTGACGCCC |
| PHGDH | NM_006623.3 | GAAAATCCACATTCTTGGGCTGAACGCGGGCCTCTGACACTGCTTACACTGCACTCTGACCCTGTAGTACAGCAATAACCGTCTAATAAAGAGCCTACCC |
| PIK3C2A | NM_002645.1 | TGGTAATTTTCAAATGTCTCTCAACTGGCAGAGATCGAGGCATGGTGGAGCTGGTTCCTGCTTCCGATACCCTCAGGAAAATCCAAGTGGAATATGGTGT |
| PIK3CA | NM_006218.2 | CCTCAGGCTTGAAGAGTGTCGAATTATGTCCTCTGCAAAAAGGCCACTGTGGTTGAATTGGGAGAACCCAGACATCATGTCAGAGTTACTGTTTCAGAAC |
| PIK3CB | NM_006219.1 | CCGCCAGTGTTGTGAGGATGCATATCTGATTTTACGACGGCATGGGAATCTCTTCATCACTCTCTTTGCGCTGATGTTGACTGCAGGGCTTCCTGAACTC |
| PIK3CD | NM_005026.3 | GAATCAACCGCGAGCGTGTCCCATTCATCCTCACCTACGACTTTGTCCATGTGATTCAGCAGGGGAAGACTAATAATAGTGAGAAATTTGAACGGTTCCG |
| PIK3R1 | NM_181504.2 | TCATGATGAGAAGACATGGAATGTTGGAAGCAGCAACCGAAACAAAGCTGAAAACCTGTTGCGAGGGAAGCGAGATGGCACTTTTCTTGTCCGGGAGAGC |
| PIK3R2 | NM_005027.2 | CCAGCCTGGGCACCCTGATTTTTAAGCCATAGACCTGGGGTCAGGGCAGGAAGGAACTTCACTCTGCTGCTTCCGAGAACCTCGGCCGTGACATTCGGGG |
| PIK3R3 | NM_003629.3 | TGATGGGACCTCCACTTATTTAGGCCTCATGTGCTTTGAAGAAGCTTTGAGAGCCAATGTGTCTTCCACGGGTCTCTTTTTTGCTACAAGTAATCAGCCC |
| PIK3R4 | NM_014602.1 | GGGAAGACCACCACTACCAGATCTATTCTTACATACAGCCGAATTGGAGGACGAGTCAAGACGCTCACATTCTGCCAAGGCTCCCACTATTTAGCCATAG |
| PKLR | NM_181871.3 | AGCTGCTATGGCAGACACCTTCCTGGAACACCTCTGCCTACTGGACATTGACTCCGAGCCCGTGGCTGCTCGCAGTACCAGCATCATTGCCACCATCGGG |
| PKM | NM_182471.1 | ATGGCAAGAGGGTGACAGCTTCCTTTCCTGTGTGTACTCTGTCCAGTTCCTTTAGAAAAAATGGATGCCCAGAGGACTCCCAACCCTGGCTTGGGGTCAA |
| PLA2G15 | NM_012320.3 | ACTACACATGGTCACCTGAGAAGGTGTTCGTGCAGACACCCACAATCAACTACACACTGCGGGACTACCGCAAGTTCTTCCAGGACATCGGCTTTGAAGA |
| PLCG1 | NM_002660.2 | GTGATGCTAGGGAACTCGGAGTTCGACAGCCTTGTTGACCTCATCAGCTACTATGAGAAACACCCGCTATACCGCAAGATGAAGCTGCGCTATCCCATCA |
| PLK1 | NM_005030.3 | GCTTGGCTGCCAGTACCTGCACCGAAACCGAGTTATTCATCGAGACCTCAAGCTGGGCAACCTTTTCCTGAATGAAGATCTGGAGGTGAAAATAGGGGAT |
| PNOC | NM_001284244.1 | CGGGGCCCGGAAGTCGGCCAGGAAGTTGGCCAATCAGAAGCGGTTCAGTGAGTTTATGAGGCAATACTTGGTCCTGAGCATGCAGTCCAGCCAGCGCCGG |
| PNP | NM_000270.2 | TTTCTTCTACCAGACCCTTCTGGTGCCAGATCCTCTTCTCAAAGCTGGGATTACAGGTGTGAGCATAGTGAGACCTTGGCGCTACAAAATAAAGCTGTTC |
| POLE | NM_006231.3 | GCAAGGCAGCCAACATGCCTGACTCTGAGCTATTCGAGCTCATCTCTGAGAACCGTTCCATGTCTCGGAAGCTGGAAGATTACGGGGAGCAGAAGTCTAC |
| PPARG | NM_005037.5 | CAGATCCAGTGGTTGCAGATTACAAGTATGACCTGAAACTTCAAGAGTACCAAAGTGCAATCAAAGTGGAGCCTGCATCTCCACCTTATTATTCTGAGAA |
| PPARGC1A | NM_013261.3 | GAACAAGCACTTCGGTCATCCCAGTCAAGCTGTTTTTGACGACGAAGCAGACAAGACCGGTGAACTGAGGGACAGTGATTTCAGTAATGAACAATTCTCC |
| PPAT | NM_002703.3 | GGAAAGTGTGGACTTCCATATGTGGAGGTGCTGTGTAAAAACCGGTATGTAGGGAGAACCTTCATTCAGCCAAACATGAGGTTAAGACAACTTGGTGTTG |
| PPM1A | NM_021003.4 | TTGAAATGGAGGATGCACATACGGCTGTGATCGGTTTGCCAAGTGGACTTGAATCGTGGTCATTCTTTGCTGTGTATGATGGGCATGCTGGTTCTCAGGT |
| PRDX1 | NM_002574.2 | GACCCATGAACATTCCTTTGGTATCAGACCCGAAGCGCACCATTGCTCAGGATTATGGGGTCTTAAAGGCTGATGAAGGCATCTCGTTCAGGGGCCTTTT |
| PRDX5 | NM_012094.4 | GGAAGGAGACAGACTTATTACTAGATGATTCGCTGGTGTCCATCTTTGGGAATCGACGTCTCAAGAGGTTCTCCATGGTGGTACAGGATGGCATAGTGAA |
| PRF1 | NM_005041.3 | ACTGTTTTTCAGGGAGGTGGCTGGGTTTACACGCTAATCCCGATTCACCCTGTCCAAACTGCCTAAGCCCTCCGCCATTCTCAAGCCCTGCAGTCACAGC |
| PRIM1 | NM_000946.2 | ACGCATCATTGACAGAGCATTGAAGGAGGACTTTGGATTTAAGCATCGTCTCTGGGTATATTCTGGAAGGAGAGGTGTTCATTGTTGGGTCTGTGATGAA |
| PRIM2 | NM_001282488.1 | GATGGAGTTTTCTGGAAGAAAGTGGAGGAAGCTGAGGTTGGCAGGTGACCAGAGGAATGCTTCCTACCCTCATTGCCTTCAGTTTTACTTGCAGCCACCT |
| PRKAA1 | NM_006251.5 | AGGCTGGATGAAAAAGAAAGTCGGCGTCTGTTCCAACAGATCCTTTCTGGTGTGGATTATTGTCACAGGCATATGGTGGTCCATAGAGATTTGAAACCTG |
| PRKAA2 | NM_006252.2 | ATAGTGGTGACCCTCAAGACCAGCTTGCAGTGGCTTATCATCTTATCATTGACAATCGGAGAATAATGAACCAAGCCAGTGAGTTCTACCTCGCCTCTAG |
| PRKAB1 | NM_006253.4 | TGAGGCCTGGCTATGGAACTAAATACAGTGTTGGTCTTGCCTGTCCTTCAAAATCAACAACAGATTGTCTCTCGGCTCCAGGGAGGTGTCATTTCTATAG |
| PRKAB2 | NM_005399.3 | AAGTGACTTTGAAAAGTTTTGTGGCACCTGACCCACCCCAGACACTAGGGCTATCAGAAGGTCTCCTTTTTAGCCCAGCACAGGCCCAGGCCACTTTGTC |
| PRKAG1 | NM_002733.3 | AAGACCTACAACAACCTAGATGTATCTGTGACTAAAGCCTTGCAACATCGATCACATTACTTTGAGGGTGTTCTCAAGTGCTACCTGCATGAGACTCTGG |
| PRKAG2 | NM_016203.3 | GGAATAGGAACGTACCACAACATTGCCTTCATACATCCAGACACTCCCATCATCAAAGCCTTGAACATATTTGTGGAAAGACGAATATCAGCTCTGCCTG |
| PRKCG | NM_002739.3 | TCATCTGGGGTATCGGAAAGCAGGGCCTGCAATGTCAAGTCTGCAGCTTTGTGGTTCATCGACGATGCCACGAATTTGTGACCTTCGAGTGTCCAGGCGC |
| PRKN | NM_004562.2 | CTTAGTGTCACTGGCAGTCCCACTAAATTACACTTCCTTACACTGGCCCGATGCGACAAATCAGGTGGCTCCCTTCTGTCACGTGGAGCACACAGTGTTT |
| PRODH2 | NM_021232.1 | CTGGTCTCGGCGACTCCTGGGCTCCCGGCTCTCAGGCGCATTTCTCCGAGCATCCGTCTATGGGCAGTTTGTGGCTGGTGAGACAGCAGAGGAGGTGAAG |
| PRPS1 | NM_002764.3 | TCTATGATCCTTGCAGAAGCCATCAGGAGAACTCACAATGGAGAATCCGTTTCTTACCTATTCAGCCATGTCCCTTTATAATAGAGTAACTTCTGAGGCT |
| PRR5 | NM_015366.3 | GTCCATGTGGCGTGTGTGTGAGTGAGACTTTTTTACTGCGTCCCGTCCCGCCAGCCCTATCGGCCTCGTCACTGGCCTTGGTCACTTTGTATTTCTGTCT |
| PSAT1 | NM_021154.3 | TGGGGTCTCCTGGGTTCAGCGGCTGTTGATTCAAGGTCAACATTGACCATTGGAGGAGTGGTTTAAGAGTGCCAGGCGAAGGGCAAACTGTAGATCGATC |
| PSMA3 | NM_152132.1 | GCAACTCTACATGATTGACCCATCAGGTGTTTCATACGGTTATTGGGGCTGTGCCATCGGCAAAGCCAGGCAAGCTGCAAAGACGGAAATAGAGAAGCTT |
| PSMA7 | NM_002792.2 | CTATACTGACGAAGCCATTGAAACAGATGATCTGACCATTAAGCTGGTGATCAAGGCACTCCTGGAAGTGGTTCAGTCAGGTGGCAAAAACATTGAACTT |
| PSMB1 | NM_002793.2 | AAGGCAGCCATCTCGCCGTGAGACAGCAAGTGTCGCGCAGCCGTGCGATGTTGTCCTCTACAGCCATGTATTCGGCTCCTGGCAGAGACTTGGGGATGGA |
| PSMB10 | NM_002801.3 | CCATCGCGGGCCTGGTGTTCCAAGACGGGGTCATTCTGGGCGCCGATACGCGAGCCACTAACGATTCGGTCGTGGCGGACAAGAGCTGCGAGAAGATCCA |
| PSMB3 | NM_002795.3 | AGAGACAGCAGTGAGAGCGGTTGCGCAGTGAAGGCTAGACCCGGTTTACTGGAATTGCTCTGGCGATCGAGGGATCCTAGTACACCGCAATCATGTCTAT |
| PSMC1 | NM_002802.2 | GTCAGAGTGGTGGTCATGGTCCTGGAGGTGGCAAGAAGGATGACAAGGACAAGAAAAAGAAATATGAACCTCCTGTACCAACTAGAGTGGGGAAAAAGAA |
| PSMD13 | NM_175932.2 | ATGCTCAACAACCTTCCTGGTGTGACATCGGTTCACAGTCGTTTCTATGATCTCTCCAGTAAATACTATCAAACAATCGGAAACCACGCGTCCTACTACA |
| PSME2 | NM_002818.2 | AGAAGAAAGAAGTCCATAAGTGTGGATTTCTCCCTGGGAATGAGAAAGTCCTGTCCCTGCTTGCCCTGGTTAAGCCAGAAGTCTGGACTCTCAAAGAGAA |
| PSPH | NM_004577.3 | GCTGTGAGGCCTAGCGAAGATGAAGATAGAATGCAAGGTAGAAAGTGCTGGATACCTTTAGAAAGCTGCAGGACTGGTGCGATGGGAGTTGAGACGTAAG |
| PTEN | NM_000314.3 | TGTGGTCTGCCAGCTAAAGGTGAAGATATATTCCTCCAATTCAGGACCCACACGACGGGAAGACAAGTTCATGTACTTTGAGTTCCCTCAGCCGTTACCT |
| PTGER4 | NM_000958.2 | CACTACGTGGACAAGCGATTGGCGGGCCTCACGCTCTTTGCAGTCTATGCGTCCAACGTGCTCTTTTGCGCGCTGCCCAACATGGGTCTCGGTAGCTCGC |
| PTGES | NM_004878.4 | TCCTGGGCTTCGTCTACTCCTTTCTGGGTCCTAACCCTTTTGTCGCCTGGATGCACTTCCTGGTCTTCCTCGTGGGCCGTGTGGCACACACCGTGGCCTA |
| PTGS1 | NM_000962.2 | ACCCCCAAGGCACCAACCTCATGTTTGCCTTCTTTGCACAACACTTCACCCACCAGTTCTTCAAAACTTCTGGCAAGATGGGTCCTGGCTTCACCAAGGC |
| PTGS2 | NM_000963.1 | GCTACAAAAGCTGGGAAGCCTTCTCTAACCTCTCCTATTATACTAGAGCCCTTCCTCCTGTGCCTGATGATTGCCCGACTCCCTTGGGTGTCAAAGGTAA |
| PTK2 | NM_005607.4 | TAGGAGCACGTCTTGCTACCCTCTTTTGAAGATGTTCTCTAGCCTTCCACCAGCAGCGAGGAATTAACCCTGTGTCCTCAGTCGCCAGCACTTACAGCTC |
| PTK6 | NM_001256358.1 | GACACGCAGGCTGTGCGGCACTACAAGATCTGGCGGCGTGCCGGGGGCCGGCTGCACCTGAACGAGGCGGTGTCCTTCCTCAGCCTGCCCGAGCTTGTGA |
| PTPN5 | NM_001039970.1 | CACTTTGTGTCCTGCTGCAATGTGTTCTGTCTGTCCATCCATCTCTGCCCTCTGTACCGGACACTGTGTCTCCTCAGCCAGGAAGGGGTAATGAGCTCCA |
| PTPRC | NM_080923.2 | GACACGGCTGACTTCCAGATATGACCATGTATTTGTGGCTTAAACTCTTGGCATTTGGCTTTGCCTTTCTGGACACAGAAGTATTTGTGACAGGGCAAAG |
| PUDP | NM_001178135.1 | TCCGCGTCGTTCGATATGAAGACAAGCCGCCACAAGGAGTTCTTCAGCTTGTTTTCCCACATTGTGCTGGGAGATGACCCCGAAGTGCAGCATGGCAAGC |
| PYCR1 | NM_006907.2 | TGGATGAAATAGGCGCCGACATTGAGGACAGACACATTGTGGTGTCCTGCGCGGCCGGCGTCACCATCAGCTCCATTGAGAAGAAGCTGTCAGCGTTTCG |
| PYCR2 | NM_013328.2 | AGGTCCTTGTGATAAAACCTCCTTAAATCTGTTCAGACCAAGCAATGCGAGCTTCCTCTCCTGTCCCATGTTGGAAGTTGCTCTGAAGGGGTGGTAGATG |
| PYCR3 | NM_023078.2 | CTGAGGGGCCCAAGAGATGGCGTCTTGGTCATTTGCCCGCATGGTTGGGCAGTTGGTTGAGGCCATGAACAGAACTTACGGTAACAGGCACGGCTGGCCC |
| RAD51 | NM_133487.2 | AGACCACCAGACCCAGCTCCTTTATCAAGCATCAGCCATGATGGTAGAATCTAGGTATGCACTGCTTATTGTAGACAGTGCCACCGCCCTTTACAGAACA |
| RAD51AP1 | NM_001130862.1 | TCTGTGAAGTCTCCCAATCAGAGTCTCCGCCTTGGCTTGTCCAGATTAGCACGAGTTAAACCTTTGCATCCAAATGCCACTAGCACCTGAGTGTGGTACA |
| RANBP2 | NM_006267.4 | AAATCAAACAACTGCAATTTCAACACCTGCCTCTTCGGAGATAAGCAAGGCTCCAAAGAGTGGATTTGAAGGAATGTTCATCAGGAAAGGACAGTGGGAT |
| RB1CC1 | NM_001083617.1 | CAGAAGTGTACCACTGCCAAACAAGAACTAGCAAATAACCTACATGTCAGACTGAAGTGGTGTTGCTTTGTAATGCTTCATGCTGATCAAGATGGAGAGA |
| RBBP5 | NM_005057.2 | ATCCATCCAGTGTGTTCTTTATGCTGGAGCCGAGATGGTCATAAACTCGTGAGTGCTTCCACTGATAACATAGTGTCACAGTGGGATGTTCTTTCAGGCG |
| RBKS | NM_022128.2 | AGACATGCTCAACAGATCCAATTTCATTGCAGCAGTCAGTGTCCAGGCTGCAGGAACACAGTCATCTTACCCTTACAAAAAAGACCTTCCGCTTACTCTG |
| RBP4 | NM_006744.3 | TCAGTTCCCATAAAACCTTCATTACACATAAAGATACACGTGGGGGTCAGTGAATCTGCTTGCCTTTCCTGAAAGTTTCTGGGGCTTAAGATTCCAGACT |
| RELA | NM_021975.3 | GAAGCATTAACTTCTCTGGAAAGGGGGGAGCTGGGGAAACTCAAACTTTTCCCCTGTCCTGATGGTCAGCTCCCTTCTCTGTAGGGAACTCTGGGGTCCC |
| REST | NM_001193508.1 | TCAGTGCAGTTATGTGGCCTCTAATCAACATGAAGTAACCCGCCATGCAAGACAGGTTCACAATGGGCCTAAACCTCTTAATTGCCCACACTGTGATTAC |
| RGN | NM_152869.3 | AGGCTATGTTGCCACCATTGGAACAAAGTTCTGTGCTTTGAACTGGAAAGAACAATCAGCAGTTGTCTTGGCCACGGTGGATAACGACAAGAAAAACAAT |
| RICTOR | NM_152756.3 | ACTCGGAGTCAACCAGCTCTAGACATAATAGTGAAAGTGAATCTGTGCCATCGAGTATGTTCATATTGGAGGATGACCGGTTTGGCAGCAGCTCTACTAG |
| RIMKLA | NM_173642.3 | CCCTTGAAACTCTACTTTTATCTAAGATCCAGACACCTGTCCTTACCCACTTTTCCAAACGGGGAAGACTCAGGTATCAGGAATTACCCATTGCACATTT |
| RIMKLB | NM_020734.2 | GAAGTATGTTAAAGAGTCTCATGGACGGGATGTACGTGTCATTGTCGTGGGAGGCCGTGTGGTTGGCACCATGTTACGTTGTTCAACAGATGGGAGAATG |
| RPIA | NM_144563.2 | TGCTAAGATCTGGGGGTTTCTTCATATTCCTGCTGTTGGAAGCAGTTGACCAGAAATGCTTGCCAGTACTGCCAAAGCACTGCTGTGAAATGTGAAGTAC |
| RPL23 | NM_000978.3 | CTTTTTTCCGGCGTTCAAGATGTCGAAGCGAGGACGTGGTGGGTCCTCTGGTGCGAAATTCCGGATTTCCTTGGGTCTTCCGGTAGGAGCTGTAATCAAT |
| RPLP0 | NM_001002.3 | CGAAATGTTTCATTGTGGGAGCAGACAATGTGGGCTCCAAGCAGATGCAGCAGATCCGCATGTCCCTTCGCGGGAAGGCTGTGGTGCTGATGGGCAAGAA |
| RPS6KA1 | NM_002953.3 | CATTTGCCAACGGTCCCAGTGACACACCAGAGGAAATCCTAACCCGGATCGGCAGTGGGAAGTTTACCCTCAGTGGGGGAAATTGGAACACAGTTTCAGA |
| RPS6KB1 | NM_003161.2 | GAGAAATTTGAAATCTCAGAAACTAGTGTGAACAGAGGGCCAGAAAAAATCAGACCAGAATGTTTTGAGCTACTTCGGGTACTTGGTAAAGGGGGCTATG |
| RPS6KB2 | NM_003952.2 | TCAAAAAGTTTCTGAAACGGAATCCCAGCCAGCGGATTGGGGGTGGCCCAGGGGATGCTGCTGATGTGCAGAGACATCCCTTTTTCCGGCACATGAATTG |
| RPTOR | NM_020761.2 | GGCTGTCTTCACAGGTCTGATGTGAAAATTCAATCACGACGTTAACCGGCTCGAGAGAGCGCCGGCCTAGAGGCTCATTATCTATTTATTTTACCAAACG |
| RRAGC | NM_022157.2 | GCACGCCACGAAACGCCATCTAGTCTGAATCCCAGCGTCGGGGCTCTGTGCCAGCTTACTCTTCACTCCAGGGTCGGATGCCACGTGCTACAGGACATGG |
| RRM1 | NM_001033.3 | ACATCCACATTGCTGAGCCTAACTATGGCAAACTCACTAGTATGCACTTCTACGGCTGGAAGCAGGGTTTGAAGACTGGGATGTATTATTTAAGGACAAG |
| RRM2 | NM_001034.1 | AGGGGTGACCCTTTAGTGAGCTTAGCACAGCGGGATTAAACAGTCCTTTAACCAGCACAGCCAGTTAAAAGATGCAGCCTCACTGCTTCAACGCAGATTT |
| RUNX1 | NM_001754.4 | CAGCCATGAAGAACCAGGTTGCAAGATTTAATGACCTCAGGTTTGTCGGTCGAAGTGGAAGAGGGAAAAGCTTCACTCTGACCATCACTGTCTTCACAAA |
| RUNX2 | NM_001024630.3 | AGAACCACAAGTGCGGTGCAAACTTTCTCCAGGAGGACAGCAAGAAGTCTCTGGTTTTTAAATGGTTAATCTCCGCAGGTCACTACCAGCCACCGAGACC |
| S100A1 | NM_006271.1 | CTGCTGCAGACGGAGCTCTCTGGCTTCCTGGATGCCCAGAAGGATGTGGATGCTGTGGACAAGGTGATGAAGGAGCTAGACGAGAATGGAGACGGGGAGG |
| S100A12 | NM_005621.1 | CAAGATGAACAGGTCGACTTTCAAGAATTCATATCCCTGGTAGCCATTGCGCTGAAGGCTGCCCATTACCACACCCACAAAGAGTAGGTAGCTCTCTGAA |
| SCD | NM_005063.4 | GTTGATTATCTTCAGCCCAGGCTTTTGCTAGATGGAATGGAAAAGCAACTTCATTTGACACAAAGCTTCTAAAGCAGGTAAATTGTCGGGGGAGAGAGTT |
| SDHB | NM_003000.2 | CTGCAGCCACAGCTCCCCGTATCAAGAAATTTGCCATCTATCGATGGGACCCAGACAAGGCTGGAGACAAACCTCATATGCAGACTTATGAAGTTGACCT |
| SDHC | NM_001035511.1 | TTGCCTACTCTCGGCCTAGAAGCAGTTATTCTCTCTCCATATTGGGCTTTGATTTGTGCTGAGGGTCAGCTTTTGGCTCCTTCTTCCTGAGACAGTGGAA |
| SDS | NM_006843.2 | GGAGTTTTATCCAGCGCCTCGTCGTATGTTTGGCTGAGCACCTGTGGCCTGGGTGCAGGTTAACTTCTTGTTATCAGGAGCCCACTATGCAGAGGCCAAA |
| SDSL | NM_138432.2 | ATGCCTGTCTTCCTCAAGTGTGAGAATGTGCAGCCCAGCGGCTCCTTCAAGATTCGGGGCATTGGGCATTTCTGCCAGGAGATGGCCAAGAAGGGATGCA |
| SEC13 | NM_001136026.2 | AACCTGCTCATCAGACAGGTCCGTCAAAATCTTTGATGTGCGCAATGGAGGGCAGATCCTTATCGCCGACCTCAGGGGTCATGAGGGTCCTGTGTGGCAA |
| SELENOK | NM_021237.3 | GCCCACCTTCGTCCTCGTCAGCATTTTGTCTAATCGCGGCCTGTGACGCTCGAAGGGCGGGGAGCAGAGGGAGATACAGAAACCGACAGGGGCCAGGCGC |
| SEM1 | NM_001201451.1 | TGGTTCCAGAGTGAAAATAGAGCCCAGTAGAGTCTGTAGCTGATGCAATATGGACATGTAGGGTGAGTGAGAAAATGCTTTTGTTGGGTTAAGCATCTGA |
| SERINC1 | NM_020755.2 | CCATGGCGAGCTGGATACCATGTTTGTGTGGAAGTGCCCCGTGTTTGCTATGCCGATGCTGTCCTAGTGGAAACAACTCCACTGTAACTAGATTGATCTA |
| SERINC2 | NM_018565.3 | GAGTGCGATTCCCGTGCCTGGTACGCAGGCCTCTTCTTCTTCACTCTCCTCTTCTACTTGCTGTCGATCGCGGCCGTGGCGCTGATGTTCATGTACTACA |
| SERINC3 | NM_006811.2 | TGCAGCGGTGCCTCATGTTTGCTGTGTAGTTGCTGTCCTAACAGTAAGAATTCCACGGTGACTCGCCTCATTTATGCTTTCATTCTCCTCCTGAGCACTG |
| SERINC5 | NM_001174071.2 | CACAATGGCTTTTGGTTCTTTAAACTTCTGCTGTTGGGGGCCATGTGCTCAGGAGCTTTCTTCATTCCAGATCAGGACACCTTTCTGAACGCCTGGCGCT |
| SH2D1A | NM_002351.4 | GCTGTATCACGGTTACATTTATACATACCGAGTGTCCCAGACAGAAACAGGTTCTTGGAGTGCTGAGACAGCACCTGGGGTACATAAAAGATATTTCCGG |
| SHMT1 | NM_148918.1 | TCGCCACTTGGTCATAATCATTTAGATGGTGGTGTAGGGCAAAGCTGTTAGAAAGATTGTAGCGTTTTACTCTCCCTGGGCTTTCCTCCGCCTTGCTGCA |
| SHMT2 | NM_001166356.1 | CCAGCCTTAACTTCTCGACAGTTCCGTGAGGATGACTTCCGGAGAGTTGTGGACTTTATAGATGAAGGGGTCAACATTGGCTTAGAGGTGAAGAGCAAGA |
| SIGLEC5 | NM_003830.3 | GTCCTGCTGCTGCAAGGGAGATCGAACCTCGGGACAGGAGTGGTTCCTGCAGCCCTTGGTGGTGCTGGTGTCATGGCCCTGCTCTGTATCTGTCTGTGCC |
| SLC16A1 | NM_003051.3 | TGGTGGCTGCTTGTCAGGCTGTGGCTTGATTGCAGCTTCTTTCTGTAACACCGTACAGCAACTATACGTCTGTATTGGAGTCATTGGAGGTCTTGGGCTT |
| SLC16A11 | NM_153357.1 | CGGCCTGAGTCTGTTCACACGCCGGGCCTTCTCAATCTTTGCTCTAGGCACAGCCCTGGTTGGGGGCGGGTACTTCGTTCCTTACGTGCACTTGGCTCCC |
| SLC16A13 | NM_201566.2 | GGTGCTCTCAGCGTTCTTCCAGTCGGCGCTTGTGTTTGGGGTGCTCCGCTCCTTTGGGGTCTTCTTCGTGGAGTTTGTGGCGGCGTTTGAGGAGCAGGCA |
| SLC16A2 | NM_006517.3 | GAGGCAAAATGAGACAAGTAGCCTTGGTTAGTCTTAGAGTTGCTGATGACTGTGGCTTCCCAAGAACTGGGTACAAGCAGTGCCCTTATTGTATGGTTGG |
| SLC16A3 | NM_004207.2 | GCTTTGGCTGCCGGCCCGTCATGCTTGTGGGGGGTCTCTTTGCGTCGCTGGGCATGGTGGCTGCGTCCTTTTGCCGGAGCATCATCCAGGTCTACCTCAC |
| SLC16A6 | NM_001174166.1 | CAGCCTCCTCTTCGTGGGCCTACTACAGTTAAACATTGTCATCTTCGGAGCACTGCTCAGACCCATCTTTATCAGAGGACCAGCGTCACCGAAAATAGTC |
| SLC16A7 | NM_001270623.1 | AAACAAGCTCAGCAGACTACCCAAAGTTAAAAGGTAGTATGAACCAAAATTAAAACTCAGGTTTACGTAACCAGAAAGATCCCCGAGGTGTTCACTACCG |
| SLC16A8 | NM_013356.2 | CGAGGCCGAGGCTGGCTGCCGAGTCTGTATAACTCAGGGTGCTCTGGGTGGGGTGGCCCAGTGACTTGGGAACACAGCTTCTTGTCTCAGAGAGCCTGGT |
| SLC1A5 | NM_001145144.1 | GGCTTGGTAGTGTTTGCCATCGTCTTTGGTGTGGCGCTGCGGAAGCTGGGGCCTGAAGGGGAGCTGCTTATCCGCTTCTTCAACTCCTTCAATGAGGCCA |
| SLC25A1 | NM_005984.2 | GGCCGGGTCTGCCTGGATGTGGCCATAGTGTTTGTCATCTATGATGAAGTGGTGAAGCTGCTCAACAAAGTGTGGAAGACGGACTAAGCCTAGAGAGGCC |
| SLC27A1 | NM_198580.1 | CCACTACCTGCCCTTAAATGAGGCAGTCTACACTCGCATCTGCTCGGGCGCCTTCGCCCTCTGAAGCTGTTCCTCTACTGGCCACAAACTCTGGGCCTGG |
| SLC2A1 | NM_006516.2 | AGGCTCCATTAGGATTTGCCCCTTCCCATCTCTTCCTACCCAACCACTCAAATTAATCTTTCTTTACCTGAGACCAGTTGGGAGCACTGGAGTGCAGGGA |
| SLC2A14 | NM_001286234.1 | GCTGCCTTATGGGACTGTGTAAAATAGCTGAGTCAGTTGAAATGCTGATCCTGGGCCGCTTGGTTATTGGCCTCTTCTGCGGACTCTGCACAGGTTTTGT |
| SLC2A3 | NM_006931.2 | GGCTGGGGGCTTGTCGCCCTTTCAGGCTCCACCCTTTGCGGAGATTATAAATAGTCATGATCCCAGCGAGACCCAGAGATGCTGTAATGGTAAGACTTTG |
| SLC2A5 | NM_003039.2 | TGTATCTTCCAACGTGGTCCCCATGTACTTAGGGGAGCTGGCCCCTAAAAACCTGCGGGGGGCTCTCGGGGTGGTGCCCCAGCTCTTCATCACTGTTGGC |
| SLC2A6 | NM_001145099.1 | GGGCGCGGGTCGGGACCCTGCAGAACAAAAGGGTGTTCCTGGCCACCTTCGCCGCAGTGCTCGGCAATTTCAGCTTTGGGTATGCCCTGGTCTACACATC |
| SLC2A8 | NM_014580.3 | TTGTCTTCTGGCTGGAGGTGCTTTTGGAGGTTGGGTGCTGGGCATTCAGTCGCTCCTCTCACGCGGCTGCCTTATCGGGAAGGAAATTTGTTTGCCAAAT |
| SLC3A1 | NM_000341.3 | TAGTTTGGATGCTGTTAAATTCCTCCTAGAAGCAAAGCACCTGAGAGATGAGATCCAAGTAAATAAGACCCAAATCCCGGACACGGTCACACAATACTCG |
| SLC3A2 | NM_001012662.2 | AAAATCCCTAGTCACACAGTATTTGAATGCCACTGGCAATCGCTGGTGCAGCTGGAGTTTGTCTCAGGCAAGGCTCCTGACTTCCTTCTTGCCGGCTCAA |
| SLC6A12 | NM_003044.3 | TACCTGTTCAGCTCCTTCACTTCTGAGCTGCCCTGGACGACCTGCAACAACTTTTGGAACACAGAGCATTGCACGGACTTTCTGAACCACTCAGGAGCCG |
| SLC6A18 | NM_182632.2 | GCTGCAGTCTGGGAACTACTGGCTGGAGATTTTCGACAATTTTGCCGCTTCCCCGAACCTGCTCATGTTGGCCTTTCTCGAGGTTGTGGGTGTCGTTTAT |
| SLC6A19 | NM_001003841.2 | GCTATGCCGGCTCCATTCCCCTGCTCATCATCGCCTTCTGCGAGATGTTCTCTGTGGTCTACGTGTACGGTGTGGACAGGTTCAATAAGGACATCGAGTT |
| SLC7A11 | NM_014331.3 | TCCATTACCAGCTTTTGTACGAGTCTGGGTGGAACTCCTCATAATACGCCCTGCAGCTACTGCTGTGATATCCCTGGCATTTGGACGCTACATTCTGGAA |
| SLC7A5 | NM_003486.5 | GGATGTGGGGAACATTGTGCTGGCATTATACAGCGGCCTCTTTGCCTATGGAGGATGGAATTACTTGAATTTCGTCACAGAGGAAATGATCAACCCCTAC |
| SLC7A9 | NM_014270.4 | TACCTCTTCTCCTGGGCCAGCCTGATCGTCATTAAGCCCACGTCCTTCGCCATCATCTGCCTCAGCTTCTCCGAGTATGTGTGTGCGCCCTTCTATGTGG |
| SMAD2 | NM_005901.5 | TGCTCAAGCATGTCATAAAGCTTCACCAATCAAGTCCCATGAAAAGACTTAATGTAACAACTCTTCTGTCATAGCATTGTGTGTGGTCCCTATGGACTGT |
| SMAD3 | NM_005902.3 | TTAAAGGACAGTTGAAAAGGGCAAGAGGAAACCAGGGCAGTTCTAGAGGAGTGCTGGTGACTGGATAGCAGTTTTAAGTGGCGTTCACCTAGTCAACACG |
| SMAD4 | NM_005359.3 | AGGTTGCACATAGGCAAAGGTGTGCAGTTGGAATGTAAAGGTGAAGGTGATGTTTGGGTCAGGTGCCTTAGTGACCACGCGGTCTTTGTACAGAGTTACT |
| SNF8 | NM_001317192.1 | TTCAAGACCAACCTGGAGGAATTTGCCAGCAAACACAAGCAGGAGATCCGGAAGAATCCTGAGTTCCGTGTGCAGTTCCAGGACATGTGTGCAACCATTG |
| SOD1 | NM_000454.4 | GGGGAAGCATTAAAGGACTGACTGAAGGCCTGCATGGATTCCATGTTCATGAGTTTGGAGATAATACAGCAGGCTGTACCAGTGCAGGTCCTCACTTTAA |
| SOD2 | NM_000636.2 | TTTGGGGTATCTGGGCTCCAGGCAGAAGCACAGCCTCCCCGACCTGCCCTACGACTACGGCGCCCTGGAACCTCACATCAACGCGCAGATCATGCAGCTG |
| SOD3 | NM_003102.2 | GGTGCAGCTCTCTTTTCAGGAGAGAAAGCTCTCTTGGAGGAGCTGGAAAGGTGCCCGACTCCAGCCATGCTGGCGCTACTGTGTTCCTGCCTGCTCCTGG |
| SOS1 | NM_005633.2 | TACCGGAGTACACTGGAAAGGATGCTTGATGTAACAATGCTACAGGAAGAGAAAGAGGAGCAGATGAGGCTGCCTAGTGCTGATGTTTATAGATTTGCAG |
| SOS2 | NM_006939.2 | AGAGACATTAGTACGTGTCCAAATTCGCCAAGCACTCCTCCTAGCACACCCTCTCCAAGGGTACCGCGTCGATGCTATGTGCTCAGTTCTAGTCAGAATA |
| SOX2 | NM_003106.2 | CTTAAGCCTTTCCAAAAAATAATAATAACAATCATCGGCGGCGGCAGGATCGGCCAGAGGAGGAGGGAAGCGCTTTTTTTGATCCTGATTCCAGTTTGCC |
| SPIB | NM_003121.3 | CTTTGTCATGTACAGACTCCCTGGGATCCTCATGTTTTGGGTGACAGGACCTATGGACCACTATACTCGGGGAGGCAGGGTAGCAGTTCTTCCAGAATCC |
| SQSTM1 | NM_003900.3 | GGGCCAGTTTCTCTGCCTTCTTCCAGGATCAGGGGTTAGGGTGCAAGAAGCCATTTAGGGCAGCAAAACAAGTGACATGAAGGGAGGGTCCCTGTGTGTG |
| SREBF1 | NM_001005291.1 | TTCGCTTTCTGCAACACAGCAACCAGAAACTCAAGCAGGAGAACCTAAGTCTGCGCACTGCTGTCCACAAAAGCAAATCTCTGAAGGATCTGGTGTCGGC |
| SREBF2 | NM_004599.2 | AGCAGCCTTTGATATACCAGAATGCAGCTACTAGCTTTCAAGTCCTTCAGCCTCAAGTCCAAAGCCTGGTGACATCCTCCCAGGTACAGCCGGTCACCAT |
| SRM | NM_003132.2 | GCAGTAAGACCTATGGCAACGTGCTGGTGTTGGACGGTGTCATCCAGTGCACGGAGAGAGACGAGTTCTCCTACCAGGAGATGATCGCCAACCTGCCTCT |
| SRR | NM_021947.1 | ACAATTGCCCTGGAAGTGCTGAACCAGGTTCCTTTGGTGGATGCACTGGTGGTACCTGTAGGTGGAGGAGGAATGCTTGCTGGAATAGCAATTACAGTTA |
| STAM2 | NM_005843.4 | CAGTGTACTCAGTCTATTCAAAGCTCCACCCTCCAGCACATTACCCACCTGCATCATCTGGGGTTCCAATGCAGACATATCCAGTTCAATCACATGGTGG |
| STAT1 | NM_007315.3 | TCATTTGCTGTATGCCATCCTCGAGAGCTGTCTAGGTTAACGTTCGCACTCTGTGTATATAACCTCGACAGTCTTGGCACCTAACGTGCTGTGCGTAGCT |
| STAT3 | NM_003150.3 | AAAGAAGGAGGCGTCACTTTCACTTGGGTGGAGAAGGACATCAGCGGTAAGACCCAGATCCAGTCCGTGGAACCATACACAAAGCAGCAGCTGAACAACA |
| STAT5A | NM_003152.2 | GAGACAGAGAGAGAGAAAGAGAGAGTGTGTGGGTCTATGTAAATGCATCTGTCCTCATGTGTTGATGTAACCGATTCATCTCTCAGAAGGGAGGCTGGGG |
| STAT6 | NM_003153.3 | AGAACATCCAGCCATTCTCTGCCAAAGACCTGTCCATTCGCTCACTGGGGGACCGAATCCGGGATCTTGCTCAGCTCAAAAATCTCTATCCCAAGAAGCC |
| STK11 | NM_000455.4 | GCTGAAGCACCAGTGCCCATCCCACCGAGCCCAGACACCAAGGACCGGTGGCGCAGCATGACTGTGGTGCCGTACTTGGAGGACCTGCACGGCGCGGACG |
| STK3 | NM_006281.3 | ACCACAAGTACAAAGACCATCTTTCATGGACTACTTTGATAAGCAAGACTTCAAGAATAAGAGTCACGAAAACTGTAATCAGAACATGCATGAACCCTTC |
| TALDO1 | NM_006755.1 | GCTGGGCGGGTCACAAGAGGACCAGATTAAAAATGCTATTGATAAACTTTTTGTGTTGTTTGGAGCAGAAATACTAAAGAAGATTCCGGGCCGAGTATCC |
| TBK1 | NM_013254.2 | ACCAGTCTTCAGGATATCGACAGCAGATTATCTCCAGGTGGATCACTGGCAGACGCATGGGCACATCAAGAAGGCACTCATCCGAAAGACAGAAATGTAG |
| TBX21 | NM_013351.1 | ACACAGGAGCGCACTGGATGCGCCAGGAAGTTTCATTTGGGAAACTAAAGCTCACAAACAACAAGGGGGCGTCCAACAATGTGACCCAGATGATTGTGCT |
| TBXAS1 | NM_001130966.2 | GCTCATTTAAAACGCTATGCGGAATCTGGGGACGCATTTGACATCCAGAGGTGCTACTGCAATTACACCACAGATGTGGTTGCCAGCGTCGCCTTTGGCA |
| TCL1A | NR_049726.1 | CCCACCCAGATAGGCCCAAGCCTGCTGCCTATCATGTGGCAGCTCTACCCTGATGGACGATACCGATCCTCAGACTCCAGTTTCTGGCGCTTAGTGTACC |
| TDO2 | NM_005651.2 | AGAGTACTTATCTCCAGCATCAGGCTTCCAGAGTTTGCAATTCCGACTATTAGAAAACAAGATAGGTGTTCTTCAGAACATGAGAGTCCCTTATAACAGA |
| TECR | NR_038104.1 | TGCACCTCGCCTGCATCTGTCACTCATTCCACTACATCAAGCGCCTGCTGGAGACGCTCTTCGTGCACCGCTTCTCCCATGGCACTATGCCTTTGCGCAA |
| TELO2 | NM_016111.3 | GAGCCAGCACCCTCAGAGGTTCGACTCGCCGTCCGGGAAGCCATTCATGCCCTCTCGTCTTCGGAGGATGGCGGCCACATCTTCTGCACCCTGGAGTCCC |
| TET2 | NM_001127208.2 | CTCATAATGTCCAAATGGGACTGGAGGAAGTACAGAATATAAATCGTAGAAATTCCCCTTATAGTCAGACCATGAAATCAAGTGCATGCAAAATACAGGT |
| TF | NM_001063.2 | CTGCTCCACCCTTAACCAATACTTCGGCTACTCGGGAGCCTTCAAGTGTCTGAAGGATGGTGCTGGGGATGTGGCCTTTGTCAAGCACTCGACTATATTT |
| TFAM | NM_003201.1 | AGGCTCTCCGAGATTGGGGTCGGGTCACTGCCTCATCCACCGGAGCGATGGCGTTTCTCCGAAGCATGTGGGGCGTGCTGAGTGCCCTGGGAAGGTCTGG |
| TFRC | NM_003234.1 | CAGTTTCCACCATCTCGGTCATCAGGATTGCCTAATATACCTGTCCAGACAATCTCCAGAGCTGCTGCAGAAAAGCTGTTTGGGAATATGGAAGGAGACT |
| TH | NM_000360.3 | GTCTACTTCGTGTCTGAGAGCTTCAGTGACGCCAAGGACAAGCTCAGGAGCTATGCCTCACGCATCCAGCGCCCCTTCTCCGTGAAGTTCGACCCGTACA |
| THBS1 | NM_003246.2 | ACCCTCGTCACATAGGCTGGAAAGATTTCACCGCCTACAGATGGCGTCTCAGCCACAGGCCAAAGACGGGTTTCATTAGAGTGGTGATGTATGAAGGGAA |
| THBS2 | NM_003247.2 | AAACATCCTTGCAAATGGGTGTGACGCGGTTCCAGATGTGGATTTGGCAAAACCTCATTTAAGTAAAAGGTTAGCAGAGCAAAGTGCGGTGCTTTAGCTG |
| TIGAR | NM_020375.2 | GCTATGTCATACTCAGCAAGGACATGCTCATATTAGGGGAAGCACATAAAGTAAGATATCCCTGAGAGAACAGTGCATCTCACACCCATTCCATGGCTTA |
| TIGIT | NM_173799.2 | TGGATCTTAGAAGACTTTTATCCTTCCACCATCTCTCTCAGAGGAATGAGCGGGGAGGTTGGATTTACTGGTGACTGATTTTCTTTCATGGGCCAAGGAA |
| TIMELESS | NM_003920.2 | AGAACTGTTACAACCGGCTCATGGGATCAGTAAAGGATCACCTGCTTCGGGAGAAAGCTCAGCAGCATGATGAGACCTATTATATGTGGGCCTTGGCTTT |
| TK1 | NM_003258.1 | GGATGGCCTGGATTCACGCCCTCTTGTTTCCTTTTGGGCTCAAAGCCCTTCCTACCTCTGGTGATGGTTTCCACAGGAACAACAGCATCTTTCACCAAGA |
| TK2 | NM_004614.3 | AGTGTTATTTCATCCAGGGAAATGCCTCGAGCCATAGAGCCTGAAATCATCTTTGTTGGCTCAGAAAATACCTTAGCTTCACTCAGCTGGACTGCATTGA |
| TKT | NM_001064.2 | TGTGCCACCCGCAACAGGACGGTGCCCTTCTGCAGCACTTTTGCAGCCTTCTTCACGCGGGCCTTTGACCAGATTCGCATGGCCGCCATCTCCGAGAGCA |
| TLR10 | NM_030956.2 | TGGTTGGATGGTCAGATTCATACACCTGTGAATACCCTTTAAACCTAAGGGGAACTAGGTTAAAAGACGTTCATCTCCACGAATTATCTTGCAACACAGC |
| TLR2 | NM_003264.3 | CAATGATGCTGCCATTCTCATTCTTCTGGAGCCCATTGAGAAAAAAGCCATTCCCCAGCGCTTCTGCAAGCTGCGGAAGATAATGAACACCAAGACCTAC |
| TLR4 | NM_138554.2 | ACTCAGAAAAGCCCTGCTGGATGGTAAATCATGGAATCCAGAAGGAACAGTGGGTACAGGATGCAATTGGCAGGAAGCAACATCTATCTGAAGAGGAAAA |
| TLR7 | NM_016562.3 | TGTGGGCACCACACAGGTGGTTGCTGCTTCAGTGCTTCCTGCTCTTTTTCCTTGGGCCTGCTTCTGGGTTCCATAGGGAAACAGTAAGAAAGAAAGACAC |
| TNF | NM_000594.2 | AGCAACAAGACCACCACTTCGAAACCTGGGATTCAGGAATGTGTGGCCTGCACAGTGAAGTGCTGGCAACCACTAAGAATTCAAACTGGGGCCTCCAGAA |
| TNFRSF17 | NM_001192.2 | TCTGACCATTGCTTTCCACTCCCAGCTATGGAGGAAGGCGCAACCATTCTTGTCACCACGAAAACGAATGACTATTGCAAGAGCCTGCCAGCTGCTTTGA |
| TNFRSF4 | NM_003327.3 | CAACTCTGCACCGTTCTAGGTGCCGATGGCTGCCTCCGGCTCTCTGCTTACGTATGCCATGCATACCTCCTGCCCCGCGGGACCACAATAAAAACCTTGG |
| TP53 | NM_000546.2 | GGGGAGCAGGGCTCACTCCAGCCACCTGAAGTCCAAAAAGGGTCAGTCTACCTCCCGCCATAAAAAACTCATGTTCAAGACAGAAGGGCCTGACTCAGAC |
| TP63 | NM_003722.4 | TTACCAGTGAGGGGCCGTGAGACTTATGAAATGCTGTTGAAGATCAAAGAGTCCCTGGAACTCATGCAGTACCTTCCTCAGCACACAATTGAAACGTACA |
| TPH1 | NM_004179.1 | TTCTGACCTGGACCATTGTGCCAACAGAGTTCTGATGTATGGATCTGAACTAGATGCAGACCATCCTGGCTTCAAAGACAATGTCTACCGTAAACGTCGA |
| TPR | NM_003292.2 | TCTAGTCACTCTGATCTTGGCCAGCTTGCTTCTCAAGGAGGTTTAGGAATGTATGAAACACCCCTGTTCCTAGCTCATGAAGAAGAGTCAGGTGGCCGAA |
| TPSAB1/B2 | NM_003294.3 | GCAGGTGAAGGTCCCCATAATGGAAAACCACATTTGTGACGCAAAATACCACCTTGGCGCCTACACGGGAGACGACGTCCGCATCGTCCGTGACGACATG |
| TPX2 | NM_012112.4 | TGGGACCTGCTCTTAACCTCAAACCTAGGACCGTCTTGCTTTGTCATTGGGCATGGAGAGAACCCATTTCTCCAGACTTTTACCTACCCGTGCCTGAGAA |
| TRAF1 | NM_005658.3 | CGAGTGATGGGTCTAGGCCCTGAAACTGATGTCCTAGCAATAACCTCTTGATCCCTACTCACCGAGTGTTGAGCCCAAGGGGGGATTTGTAGAACAAGCC |
| TRAF6 | NM_145803.2 | TTGCAAAACGTGAGATTCTTTCTCTGATGGTGAAATGTCCAAATGAAGGTTGTTTGCACAAGATGGAACTGAGACATCTTGAGGATCATCAAGCACATTG |
| TRAT1 | NM_016388.2 | ACAGAGGACACAGAAGGACTTGGCAGCAGGGTGATGACCTGATCATTTGTTGATGGGATGGTGGCTTACCTCTTATTCACAGCTTACACTTATGCATGCC |
| TTPA | NM_000370.3 | ATTACAGTCGTGAGCCATCGCGCCTGGCCGTGATAGAAACTTTCAGCTGAGGAGTCTATATGCCATACTACTCTATGTGGCATCTTTAGGTCTCTGTGAA |
| TTR | NM_000371.3 | CCTACGGGCACCGGTGAATCCAAGTGTCCTCTGATGGTCAAAGTTCTAGATGCTGTCCGAGGCAGTCCTGCCATCAATGTGGCCGTGCATGTGTTCAGAA |
| TXN | NM_003329.2 | CAGCCAAGATGGTGAAGCAGATCGAGAGCAAGACTGCTTTTCAGGAAGCCTTGGACGCTGCAGGTGATAAACTTGTAGTAGTTGACTTCTCAGCCACGTG |
| TXN2 | NM_012473.3 | CTGGTGGCCTGACTGTAACACCCAACCCAGCCCGGACAATATACACCACGAGGATCTCCTTGACAACCTTTAATATCCAGGATGGACCTGACTTTCAAGA |
| TXNRD1 | NM_001093771.1 | AGTGATGATCTTTTCTCCTTGCCTTACTGCCCGGGTAAGACCCTGGTTGTTGGAGCATCCTATGTCGCTTTGGAGTGCGCTGGATTTCTTGCTGGTATTG |
| TYMP | NM_001953.3 | GCTGGACCAGGCGGGCTGCTGTATCGTGGGTCAGAGTGAGCAGCTGGTTCCTGCGGACGGAATCCTATATGCAGCCAGAGATGTGACAGCCACCGTGGAC |
| TYMS | NM_001071.2 | GTCAGTCTTTAGGGGTTGGGCTGGATGCCGAGGTAAAAGTTCTTTTTGCTCTAAAAGAAAAAGGAACTAGGTCAAAAATCTGTCCGTGACCTATCAGTTA |
| UBE2C | NM_007019.2 | CTTTTAAGAAGTACCTGCAAGAAACCTACTCAAAGCAGGTCACCAGCCAGGAGCCCTGACCCAGGCTGCCCAGCCTGTCCTTGTGTCGTCTTTTTAATTT |
| UBE2T | NM_014176.3 | AAACAAAAGGCTGATGAGGAAGAGATGCTTGATAATCTACCAGAGGCTGGTGACTCCAGAGTACACAACTCAACACAGAAAAGGAAGGCCAGTCAGCTAG |
| UCK1 | NM_031432.3 | GGAGTTGCTGGGACAGAACGAGGTGGAACAGCGGCAGCGGAAGGTGGTCATCCTGAGCCAGGACAGGTTCTACAAGGTCCTGACGGCAGAGCAGAAGGCC |
| UCK2 | NM_012474.3 | CAGTACATTACGTTCGTCAAGCCTGCCTTTGAGGAATTCTGCTTGCCAACAAAGAAGTATGCTGATGTGATCATCCCTAGAGGTGCAGATAATCTGGTGG |
| UCKL1 | NM_001193379.1 | GGGAAGAGTGTCAAGGTGCCCATTTATGACTTCACCACGCACAGCCGGAAGAAGGACTGGAAAACACTGTATGGTGCAAACGTCATCATCTTTGAGGGCA |
| UMPS | NM_000373.2 | ACCGGAGTTGGGGTTTGGGCTCATTTTTTCCCCTAGCCAGCAATTATGGACCAGTAGTAACACAAGTGACAGCTTCCTGTGACTGACTTCACAATTAGGA |
| UPP1 | NM_003364.2 | TCATAACAGAGCAGGCAGTGGATACCTGCTTCAAGGCAGAGTTTGAGCAGATTGTCCTGGGGAAGCGGGTCATCCGGAAAACGGACCTTAACAAGAAGCT |
| UPP2 | NM_001135098.1 | CACGGTGCTGCGATGTCACCATTATTAGAATCGGTACATCAGGGGGAATAGGGATTGCACCAGGGACTGTTGTAATAACGGATATAGCTGTAGACTCCTT |
| UQCR10 | NM_001003684.1 | CGACGTTGACTTCGAAATTGTACTCCCTGCTGTTCCGCAGGACCTCCACCTTCGCCCTCACCATCATCGTGGGCGTCATGTTCTTCGAGCGCGCCTTCGA |
| UQCR11 | NM_006830.2 | CACGTTGCATTAAACCTCACTGAAACCTGCTCCGTGCCCGGATGTTGATCATGCTGGTGGCTTGGTTACTGTGACTGTAGCTGGAGTGGCACAGGTGACC |
| UQCRQ | NM_014402.4 | AGGAATCCCCAATGTTCTGCGCCGCATTCGGGAGTCTTTCTTTCGCGTGGTGCCGCAGTTTGTAGTGTTTTATCTTATCTACACATGGGGGACTGAAGAG |
| USP8 | NM_001128610.1 | CGGAGTCTGAAAGATGCACTTTTCAAGTGGGAAAGTAAAACTGTCCTGCGCAATGAGCCTTTGGTTTTAGAGGGAGGCTATGAAAACTGGCTCCTTTGTT |
| VEGFA | NM_001025366.1 | GAGTCCAACATCACCATGCAGATTATGCGGATCAAACCTCACCAAGGCCAGCACATAGGAGAGATGAGCTTCCTACAGCACAACAAATGTGAATGCAGAC |
| VHL | NM_000551.2 | GGAAGACTGAGGCATCCGTGAGGCAGGGACAAGTCTTTCTCCTCTTTGAGACCCCAGTGCCTGCACATCATGAGCCTTCAGTCAGGGTTTGTCAGAGGAA |
| VPS28 | NM_016208.3 | GCCCGCTGGCCATGGAGCGGATCAAGGAGGACCGGCCCATCACCATCAAGGACGACAAGGGCAACCTCAACCGCTGCATCGCAGACGTGGTCTCGCTCTT |
| WASHC4 | NM_015275.1 | CCAATGACCAAAACCTCAGTTAAGGCATTGTGCAGGCTTGTTGAACTTCTCAAGGCAATAGAGCATATGTTCTACAGGAGAAGCATGGTTGTGGCTGATT |
| WDR45 | NM_007075.3 | TCCAAGAACGTCAACTCTGTCATTGCCATCTGCGTAGATGGGACCTTCCACAAATATGTCTTCACTCCTGATGGAAACTGCAACAGAGAGGCTTTCGACG |
| WNT1 | NM_005430.2 | GAGTCTGCAACTGGTACTCGAGCCCAGTCTGCAGCTGTTGAGCCGCAAACAGCGGCGTCTGATACGCCAAAATCCGGGGATCCTGCACAGCGTGAGTGGG |
| WNT2 | NM_003391.2 | GATGTAACAAGGTGGGGACGTGTGTCCTTTGGTACTATGGTGTGTTGTATCTTTGTAAGAGCAAAAGCCTCAGAAAGGGATTGCTTTGCATTACTGTCCC |
| WRN | NM_000553.4 | TCTGAGAAATCCTCAGATCACCTGTACTGGTTTTGATCGACCAAACCTGTATTTAGAAGTTAGGCGAAAAACAGGGAATATCCTTCAGGATCTGCAGCCA |
| XCL1/2 | NM_003175.3 | GTAGTCTCTGGCACCCTGTCCGTCTCCAGCCAGCCAGCTCATTTCACTTTACACCCTCATGGACTGAGATTATACTCACCTTTTATGAAAGCACTGCATG |
| XDH | NM_000379.3 | AGTATTTCTCAGCATTCAAGCAGGCCTCCCGGAGAGAAGATGACATTGCCAAGGTAACCAGTGGCATGAGAGTTTTATTCAAGCCAGGAACCACAGAGGT |
| XRCC2 | NM_005431.1 | CTCTGAGGAAATGTTCTCAGTGCTTAGAGAAGCTTGTAAATGACTATCGCCTGGTTCTTTTTGCAACGACACAAACTATAATGCAGAAAGCCTCGAGCTC |
| YWHAZ | NM_003406.2 | CTTGGTGGCCATGTACTTGGAAAAAGGCCGCATGATCTTTCTGGCTCCACTCAGTGTCTAAGGCACCCTGCTTCCTTTGCTTGCATCCCACAGACTATTT |
| ZAP70 | NM_001079.3 | GGAGCTCAAGGACAAGAAGCTCTTCCTGAAGCGCGATAACCTCCTCATAGCTGACATTGAACTTGGCTGCGGCAACTTTGGCTCAGTGCGCCAGGGCGTG |
| ZNF100 | NM_173531.3 | CGGGTTTGGCGGGGTCTTTGTCTCTCGCTGTAGCCGGAGCTCCAGGTTTTGCTCTCACTTCTCTGTGTCTTCTGCTCCTAGGGGCCTAGCCTGTGTGGCC |
| ZNF136 | NM_003437.2 | ATCCCTGGAGTGAAACTCTGTGAAAGCATTGTATATGGAGAAGTCAGCATGGGTCAGTCATCCCTTAATAGACACATCAAAGATCACAGTGGACATGAAC |
| ZNF253 | NM_021047.2 | CAAACATAAAAGAACTCATACTGGAGAGAAACCCTACAAATGTGAAGAATGTGGCAAATCCTTTACTGCATCCTCAACTCTAACTACACATAAGAGAATT |
| ZNF254 | NM_203282.3 | GTCCTCAACCCTAACTAAACATAAGAGAATTCATACTGGAGAGCAACCCTACAAATGGGAAAAATTTGGCAAAGCCTTTAATCGGTCCTCGCACCTCACC |
| ZNF43 | NM_003423.2 | TTTACCAGGCACCTATAATCCCAGCTACTTCAAAGGCTGAAGCAGGAGAATCACTTGAACCTGGAAAGTGGAGGTCTCAGTGAGCCGAAATCATACCATT |
| ZNF610 | NM_001161427.1 | ACTGACTCATCCAGAGCAATTTCTAGTCCTGCAAGCGCCATTCATGGATTGACTTATAAACAGTCATGCTATGTGATGAAGAAGCCCAGAAGAGGAAAGC |
| ZNF675 | NM_138330.2 | TGTGAGAAACTCTACAAATGTCAAGAATGTGACAGAACTTTTAACCAATTCTCAAACCTTACTGAATATAAAAAAGATTATGCTCGAGAGAAACCATACA |
| ZNF682 | NM_001077349.1 | TAAACGGTGCTCACATCTTAATGAACATAAGAGAGTTCAAAGAGGAGAGAAATCCTGCAAGTATAAAAAATGTGGGGAAGCTTTTAATCACTGCTCAAAC |
| ZNF708 | NM_021269.2 | AGTGATACTGAGAAGATATGGAAAATGTGGATATCAGAAAGGCTGTAAAAGTGTGGATGAGCATAAGTTGCACAAAGGAGGTCACAAGGGACTTAACCGG |
| ZNF85 | NM_001256171.1 | TGGGTATTACTGTTTCTAAGCCAGACCTGATCACTTGTCTGGAGCAAGGGAAAGAGGCCTGGAGTATGAAGAGACATGAGATCATGGTGGCCAAACCCAC |
| ZNF91 | NM_003430.2 | AACAAATATAGACAAAAAGCCATTAATACCTGCTCACATCGTACTCAAATTCAGAGCGTTCATACTAAATAAGAAGTGTAATTACTGTCAAACGACTGAT |
| ZNF93 | NM_031218.3 | CACAGTTAACCTGAGGAAATGCTCTCTGGTACTCACTGAAAGCCACACTCATCCACATCCTGATACAAGGCCCACCATATGCAGACCTGACTGCCAAAAC |


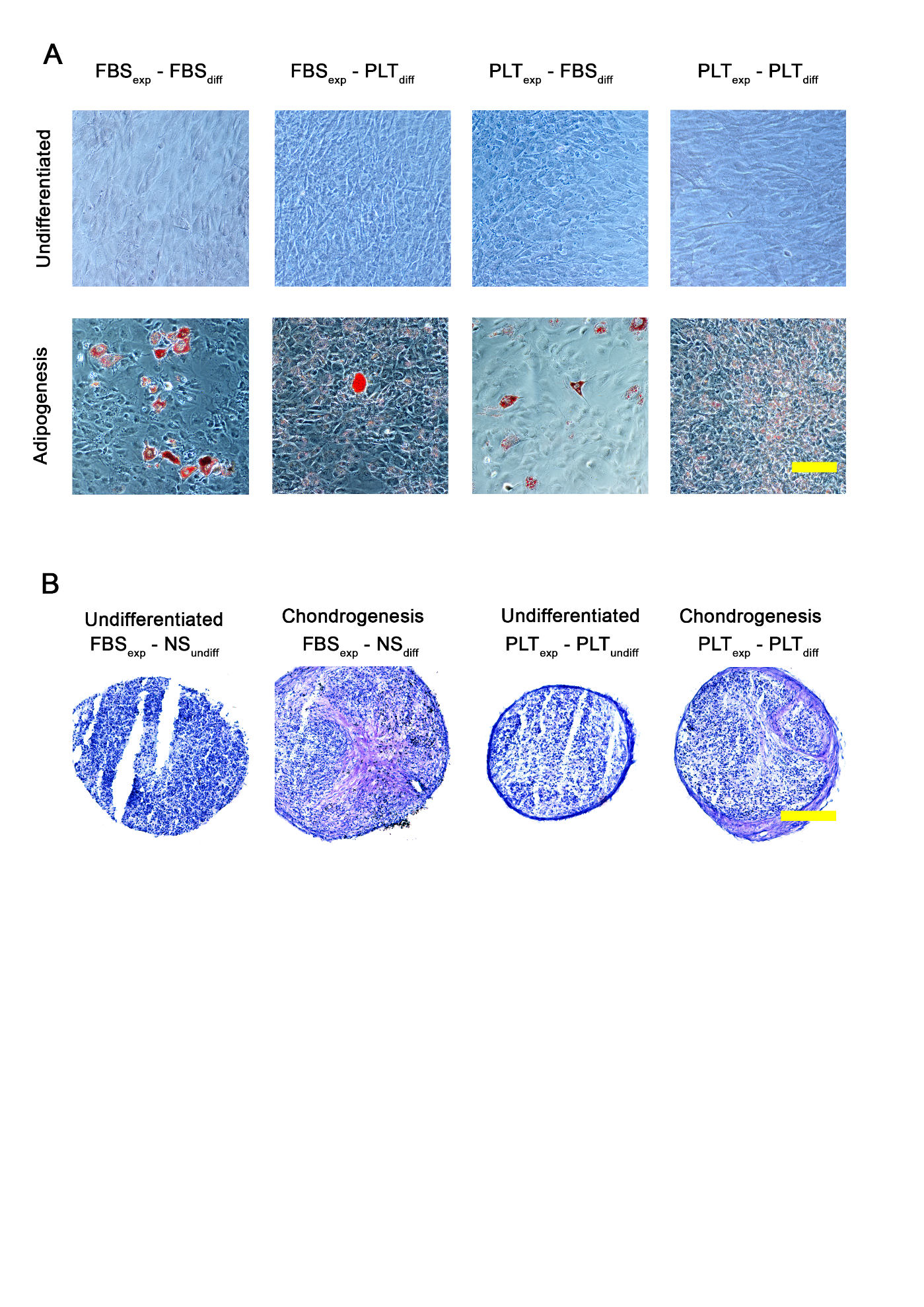


**Supplementary Fig. 1:** **Effects of FBS and PLT supplementation on expansion and differentiation of BMSCs towards adipogenic and chondrogenic phenotypes.** **A)** Oil Red O staining of 3-week differentiated 2D cultures. **B)** Toluidine blue staining of chondrogenic and undifferentiated BMSC pellets after 4 weeks of incubation. Scale bar = 200 μm. FBS_exp_ and PLT_exp_ indicate those cultures expanded in 10% FBS or 5% PLT respectively. FBS_undiff_, PLT_undiff_, FBS_diff_, PLT_diff_, NS_undiff_, NS_diff_ indicate whether the cultures were differentiated (diff) or used as a control (undiff) in FBS, PLT or No Serum (NS) conditions. Sections from a representative donor are shown.
